# In vivo self-assembled small RNAs as a new generation of RNAi therapeutics

**DOI:** 10.1038/s41422-021-00491-z

**Published:** 2021-03-29

**Authors:** Zheng Fu, Xiang Zhang, Xinyan Zhou, Uzair Ur-Rehman, Mengchao Yu, Hongwei Liang, Hongyuan Guo, Xu Guo, Yan Kong, Yuanyuan Su, Yangyang Ye, Xiuting Hu, Wei Cheng, Jinrong Wu, Yanbo Wang, Yayun Gu, Sheng-feng Lu, Dianqing Wu, Ke Zen, Jing Li, Chao Yan, Chen-Yu Zhang, Xi Chen

**Affiliations:** 1 Nanjing Drum Tower Hospital Center of Molecular Diagnostic and Therapy, Chinese Academy of Medical Sciences Research Unit of Extracellular RNA, State Key Laboratory of Pharmaceutical Biotechnology, Jiangsu Engineering Research Center for MicroRNA Biology and Biotechnology, NJU Advanced Institute of Life Sciences (NAILS), Institute of Artificial Intelligence Biomedicine, School of Life Sciences, Nanjing University, Nanjing, Jiangsu China; 2 Chemistry and Biomedicine Innovation Center, Nanjing University, Nanjing, Jiangsu, China; 3 State Key Laboratory of Reproductive Medicine, Center for Global Health, School of Public Health, Nanjing Medical University, Nanjing, Jiangsu, China; 4 Cancer Institute, The Affiliated Hospital of Qingdao University, Qingdao, Shandong, China; 5 Department of General Surgery, Jiangsu Province Hospital of Chinese Medicine, Affiliated Hospital of Nanjing University of Chinese Medicine, Nanjing, Jiangsu, China; 6 Department of Pathology, Jinling Hospital, Nanjing University School of Medicine, Nanjing, Jiangsu, China; 7 Key Laboratory of Acupuncture and Medicine Research of Ministry of Education, Nanjing University of Chinese Medicine, Nanjing, Jiangsu, China; 8 Department of Pharmacology, Vascular Biology and Therapeutic Program, Yale University School of Medicine, New Haven, CT USA

**Keywords:** RNAi, siRNAs

## Abstract

RNAi therapy has undergone two stages of development, direct injection of synthetic siRNAs and delivery with artificial vehicles or conjugated ligands; both have not solved the problem of efficient in vivo siRNA delivery. Here, we present a proof-of-principle strategy that reprogrammes host liver with genetic circuits to direct the synthesis and self-assembly of siRNAs into secretory exosomes and facilitate the in vivo delivery of siRNAs through circulating exosomes. By combination of different genetic circuit modules, in vivo assembled siRNAs are systematically distributed to multiple tissues or targeted to specific tissues (e.g., brain), inducing potent target gene silencing in these tissues. The therapeutic value of our strategy is demonstrated by programmed silencing of critical targets associated with various diseases, including EGFR/KRAS in lung cancer, EGFR/TNC in glioblastoma and PTP1B in obesity. Overall, our strategy represents a next generation RNAi therapeutics, which makes RNAi therapy feasible.

## Introduction

RNA interference (RNAi) offers an opportunity to specifically target mRNAs and modulate the expression of corresponding proteins before their biogenesis, and has therefore been proposed as a promising therapeutic tool to manipulate the expression of disease-related genes, especially the genes that are considered undruggable using traditional approaches.^1–3^ However, RNAi therapy has encountered many problems and falls way behind expectation during clinical translation. The development of RNAi therapy has undergone two major stages. In the first stage, naked small interfering RNAs (siRNAs) or chemically modified stable siRNAs were synthesized and directly injected for systematic delivery; yet these siRNAs cannot effectively pass biological barriers and reach target genes.^3^ In the second stage, various delivery vehicles (e.g., lipid nanoparticles, cationic polymers and viruses) or conjugated ligands (e.g., triantennary N-acetylgalactosamine (GalNAc)) were invented to increase the efficiency of siRNA delivery in vivo.^4,5^ The recent FDA approval of the first (Patisiran, siRNA is formulated as a lipid complex for the delivery to hepatocytes^6^) and second (Givosiran, siRNA is conjugated to a GalNAc ligand that enables asialoglycoprotein receptors-mediated targeted delivery to hepatocytes^7^) siRNA drugs marked the beginning of the era of RNAi therapeutics. Despite some successful cases, in general the translation of siRNA therapeutics to wide clinical use is still hindered by a major hurdle associated with in vivo delivery, especially for extrahepatic delivery. A common feature of the current delivery strategies is that the siRNAs are pre-assembled with vehicles or ligands in vitro. However, these artificial complexes are often plagued by problems like low immunocompatibility, high toxicity, insufficient circulation stability and limited tissue accessibility (mainly liver) when administered in vivo.^8^ Thus, a more concerted effort towards developing a safe, precise and efficient delivery platform for siRNAs is crucial for next-generation RNAi therapeutics.

Recently, our group discovered that endogenous cells could selectively package microRNAs (miRNAs) into exosomes and secrete exosomes to deliver miRNAs into recipient cells, where the secreted miRNAs, at a relatively low concentration, robustly blocked target gene expression.^9–11^ We also provided evidence that cells could be engineered to produce specific exosomes that carry artificial small RNAs (e.g., miRNA mimics).^9^ Because of their intrinsic nature, exosomes are biocompatible with the host immune system and have the innate ability to protect and transport small RNAs across biological barriers in vivo,^12,13^ hence the potential to overcome the problems associated with siRNA delivery. Although several studies have since attempted to use exosomes to deliver therapeutic siRNAs and miRNAs,^14,15^ they all have to harvest huge amounts of exosomes from cell culture before in vivo delivery, making large scale clinical translation unaffordable.^16^ Moreover, the fragility and complicated production/purification protocols of exosomes are not amenable to Good Manufacturing Practice (GMP) standards. Because synthetic biology enables the design of composable, controllable and multifunctional genetic circuits (a genetic circuit is a combination of biological parts that together execute a defined function within a host organism) to reprogramme cells and even allow cells to self-assemble into new, user-defined tissues,^17,18^ we proposed an intriguing idea that host tissues may be engineered and converted to live biogenerators of RNAi therapeutics through the integration of the naturally existing circulating exosome system with artificial genetic circuits. Here, we designed composable and programmable genetic circuits that use the liver as a tissue chassis to direct the self-assembly of exogenous siRNAs into secretory exosomes and facilitate the systematic/targeted delivery of self-assembled siRNAs in vivo.

## Results

### Design and construction of the core genetic circuits to direct the self-assembly and release of siRNAs in vitro

**Fig. 1:**
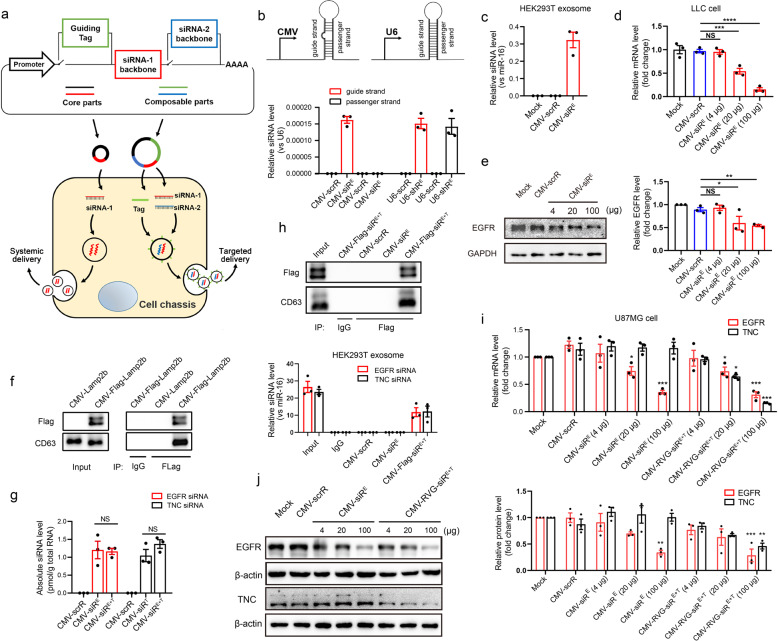
Construction and characterization of the genetic circuits. **a** Schematic description of the architecture of the genetic circuit. The core circuit contains a promoter part (black) and an siRNA-expressing part (red). When the core circuit is placed in a cell/tissue chassis, the promoter drives the transcription of the siRNA and directs the siRNA payload into exosomes. Two composable, plug-in parts are driven by the same promoter of the core circuit and incorporated into the core circuit structure: one encodes a guidance tag (green) that is anchored on the exosome surface to enable targeting of exosomes to cells and tissues, and the other part (blue) expresses a different tandem siRNA. The core circuit design facilitates the systematic distribution of siRNAs to multiple tissues, while the composable-core circuit increases the simultaneous accumulation of two siRNAs in specific tissues. **b** Quantitative RT-PCR analysis of the levels of the desired guide strand and undesired passenger strand generated using the pre-miRNA strategy (EGFR siRNA is embedded in pre-miR-155 and is controlled by a CMV promoter, named CMV-siR^E^) or the shRNA strategy (EGFR siRNA is placed in an shRNA and is controlled by a U6 promoter, named U6-shR^E^) in HEK293T cells. A CMV- or U6-directed scrambled RNA (CMV-scrR or U6-scrR) was used as a control. RNU6A (*U6*) was selected as a reference gene for normalization of cellular siRNA levels (*n* = 3 in each group). **c** Quantitative RT-PCR analysis of EGFR siRNA levels in exosomes derived from HEK293T cells transfected with the CMV-scrR or CMV-siR^E^ circuit. miR-16, a universally expressed miRNA, was selected as a reference gene for the normalization of siRNA levels in exosomes (*n* = 3 in each group). **d** Quantitative RT-PCR analysis of *EGFR* mRNA levels in LLC cells treated with exosomes derived from HEK293T cells transfected with CMV-scrR or CMV-siR^E^ (*n* = 3 in each group). Different doses (total protein contents of exosomes were 4, 20 and 100 μg, respectively) of exosomes were added to reveal the dose-dependent effect. **e** Western blot analysis of EGFR protein levels in LLC cells treated with exosomes derived from HEK293T cells transfected with CMV-scrR or CMV-siR^E^. Different doses of exosomes were added to reveal the dose-dependent effect. Left panel, representative western blots. Right panel, quantitative analysis (*n* = 3 in each group). **f** A CMV-directed Flag-Lamp2b fusion construct (CMV-Flag-Lamp2b) or a CMV-directed Lamp2b construct (CMV-Lamp2b) was transfected into HEK293T cells. Exosomes were then isolated and either directly loaded for western blotting with anti-Flag and anti-CD63 antibodies (equal CD63 band densities indicate similar exosome levels) or immunoprecipitated with IgG or anti-Flag beads before western blotting. **g** The CMV-siR^E^, CMV-siR^T^ or CMV-siR^E+T^ circuits were transfected into HEK293T cells. A quantitative RT-PCR assay was performed to assess the levels of EGFR and TNC siRNAs in transfected HEK293T cells (*n* = 3 in each group). **h** CMV-scrR, CMV-siR^E^ or CMV-Flag-siR^E+T^ circuits were transfected into HEK293T cells. Exosomes were then isolated and either directly loaded for western blotting with anti-Flag and anti-CD63 antibodies or immunoprecipitated with IgG or anti-Flag beads before western blotting. A quantitative RT-PCR assay was performed to assess the levels of EGFR and TNC siRNAs in immunoprecipitated exosomes (*n* = 3 in each group). **i** Quantitative RT-PCR analysis of *EGFR* and *TNC* mRNA levels in U87MG cells treated with exosomes derived from HEK293T cells transfected with the CMV-scrR, CMV-siR^E^ or CMV-RVG-siR^E+T^ circuit (*n* = 3 in each group). Different doses of exosomes were added to reveal the dose-dependent effect. **j** Western blot analysis of EGFR and TNC protein levels in U87MG cells treated with exosomes derived from HEK293T cells transfected with the CMV-scrR, CMV-siR^E^ or CMV-RVG-siR^E+T^ circuit. Different doses of exosomes were added to reveal the dose-dependent effect. Left panel, representative western blots. Right panel, quantitative analysis (*n* = 3 in each group). Values are presented as the means ± SEM. Significance was determined using one-way ANOVA followed by Dunnett’s multiple comparison in **d**, **e**, **g**, **i**, **j**. *P < 0.05; **P < 0.01; ***P < 0.005; ns, not significant.

First, we rationally designed a hierarchical genetic circuit architecture that allows the free combination of different functional modules (Fig. 1a). The core circuit, consisting of a promoter part and an siRNA-expressing part, was designed to produce and organize siRNAs as the payload for exosomes (Fig. 1a). Other composable parts (plug-ins) could then be integrated into the framework of the core circuit to achieve plug-and-play functionality. As an example, two types of composable parts were incorporated to optimize the siRNA effects: one modifying the membrane-anchored proteins of exosomes to enable tissue selectivity; the other enabling the co-expression of a second siRNA for the simultaneous downregulation of two molecular targets (Fig. 1a).

For the core circuit construct, we designed a scheme that encodes an optimized siRNA expression backbone part (to maximize guide strand expression while minimizing undesired passenger strand expression) under the control of a promoter part (Fig. 1a). Epidermal growth factor receptor (*EGFR*), one of the most potent oncogenes that is frequently mutated and highly expressed in a variety of human cancers (e.g., lung cancer and glioblastoma),^19,20^ was selected as the siRNA target. Human embryonic kidney 293T cells (HEK293T) and mouse hepatoma cells (Hepa 1-6) were chosen as the cell chassis for in vitro assembly of siRNA. To optimize the siRNA production efficiency, we compared two classic design strategies: one using the CMV promoter to express an miRNA precursor (pre-miRNA) and substituting the miRNA sequence with siRNA, and the other using the U6 promoter to express a short hairpin RNA (shRNA).^21,22^ We first examined a series of pre-miRNA structures and selected pre-miR-155 as the optimal backbone for the production of siRNA (Supplementary information, Fig. S1). Then, the siRNA production efficiency of a CMV-directed pre-miRNA encoding an EGFR siRNA (CMV-siR^E^) and a U6-directed EGFR shRNA (U6-shR^E^) was compared (Fig. 1b). Both strategies had similar efficiencies in driving the transcription of the guide strand of EGFR siRNA; however, compared with the shRNA approach, the pre-miRNA approach produced less or no passenger strand (the passenger strand is seemingly degraded during the biogenesis of mature guide strand) (Fig. 1b and Supplementary information, Fig. S2a). Therefore, the CMV-driven pre-miRNA design was chosen to avoid off-target effects.

Next, we examined whether the core circuit directs the loading of siRNA into exosomes. HEK293T cells were transfected with the control scrambled circuit (CMV-scrR) or CMV-siR^E^ circuit, and the exosomes in cell culture medium were characterized. Nanoparticle tracking analysis (NTA) revealed that similar amounts of exosomes were secreted in each group with similar size distribution, peaking at 128–131 nm (Supplementary information, Fig. S3a). Transmission electron microscopy (TEM) confirmed that the purified exosomes exhibited a typical round-shaped vesicular morphology and had correct size (Supplementary information, Fig. S3b). Moreover, enrichment of specific exosome markers (CD63, TSG101 and CD9) was only detected in purified exosomes but not in cell culture medium (Supplementary information, Fig. S3c). These results demonstrate that transfection with genetic circuits did not affect the size, structure or number of exosomes generated by HEK293T cells. Finally, a significant amount of EGFR siRNA was detected in exosomes derived from HEK293T and Hepa 1-6 cells transfected with the CMV-siR^E^ circuit (Fig. 1c and Supplementary information, Fig. S2b). When these exosomes were incubated with mouse Lewis lung carcinoma (LLC) cells, a dose-dependent knockdown of EGFR expression was achieved (Fig. 1d, e), suggesting that the exosomal siRNAs were biologically functional.

### Construction of the composable-core circuits to direct the self-assembly and release of siRNAs in vitro

For the design of composable guidance part, a sequence encoding a tissue-targeting tag fused to the N-terminus of Lamp2b (a canonical exosome membrane protein) was inserted downstream of the CMV promoter (Fig. 1a). This tag should be anchored to the exosome surface via Lamp2b, thus guiding the delivery of the exosomal cargo to the desired tissue. Specifically, a central nervous system-targeting RVG peptide was chosen as the tag to direct exosomes to the brain (RVG has been shown to facilitate transcytosis of exosomes across the blood-brain barrier and into neuronal cells).^14,23^ The efficiency of the promoters in initiating the expression of the RVG-Lamp2b fusion protein was first assessed. The CMV promoter was somewhat effective in generating *RVG-Lamp2b* mRNA and marker protein eGFP in HEK293T cells, whereas the U6 promoter was not effective (Supplementary information, Fig. S4), confirming the advantage of the CMV promoter for linking the core circuit and composable part. The correct display of the guidance tag on the exosome surface was then verified using immunoprecipitation. A Flag epitope tag was used to replace RVG because of the lack of an anti-RVG antibody. After transfection of HEK293T and Hepa 1-6 cells with a CMV-directed Flag-Lamp2b circuit, intact exosomes were successfully immunoprecipitated with anti-Flag beads (Fig. 1f and Supplementary information, Fig. S2c), demonstrating the precise localization of the guidance tag. For the design of additional siRNA-expressing parts, tenascin-C (*TNC*), a critical oncogene associated with many cancer types, including glioblastoma,^24^ was selected as the second siRNA target. TNC siRNA was also embedded in the pre-miR-155 backbone and inserted downstream of the EGFR siRNA expression cassette (Fig. 1a). Regardless of the individual (CMV-siR^E^ or CMV-siR^T^) or tandem (CMV-siR^E+T^) transcription, equal amounts of EGFR and TNC siRNA were detected (Fig. 1g and Supplementary information, Fig. S2d). Overall, these results show that all composable parts could be functionally integrated into the core circuit.

Next, we examined whether the composable-core circuit enables the self-assembly of siRNAs into exosomes in vitro. HEK293T cells were transfected with a CMV-directed circuit encoding both EGFR and TNC siRNAs along with an RVG tag (CMV-RVG-siR^E+T^). The exosomes derived from the cell culture medium displayed typical morphology and size distribution (Supplementary information, Fig. S3), indicating that modification with the composable-core circuit did not alter the physical properties of exosomes. Moreover, a complete composable-core circuit encoding both EGFR and TNC siRNAs and a Flag tag (CMV-Flag-siR^E+T^) was constructed. Exosomes generated from the CMV-Flag-siR^E+T^ circuit-transfected HEK293T and Hepa 1-6 cells were successfully immunoprecipitated with anti-Flag beads, and both EGFR and TNC siRNAs were abundantly present in the immunoprecipitated exosomes (Fig. 1h and Supplementary information, Fig. S2e). Furthermore, since siRNA processing is dependent on Argonaute 2 (AGO2) and the proper loading of siRNA into AGO2 is expected to enhance the on-target effects of siRNA,^21^ another immunoprecipitation experiment was performed to assess the association of AGO2 with siRNAs in exosomes. EGFR and TNC siRNAs were readily detected in the exosomes precipitated with anti-AGO2 beads (Supplementary information, Fig. S5), suggesting that our design guarantees the loading of siRNA into the RNA-induced silencing complex (RISC) and facilitates the efficient transport of AGO2-bound siRNA into exosomes. Finally, to investigate whether the in vitro assembled siRNAs are functional, exosomes derived from HEK293T cells transfected with the CMV-RVG-siR^E+T^ circuit were incubated with the U87MG glioblastoma cells. A dose-dependent downregulation of EGFR and TNC expression in U87MG cells was achieved (Fig. 1i, j). Moreover, the RVG tag on exosome surface did not affect the silencing efficacy of EGFR and TNC siRNAs on their targets (Supplementary information, Fig. S6). These results establish the genetic circuit as an organic combination of multiple composable parts that direct the self-assembly and release of siRNAs in vitro.

### Genetic circuits use the liver as a tissue chassis and direct the systematic delivery of siRNAs to multiple tissues in vivo

**Fig. 2:**
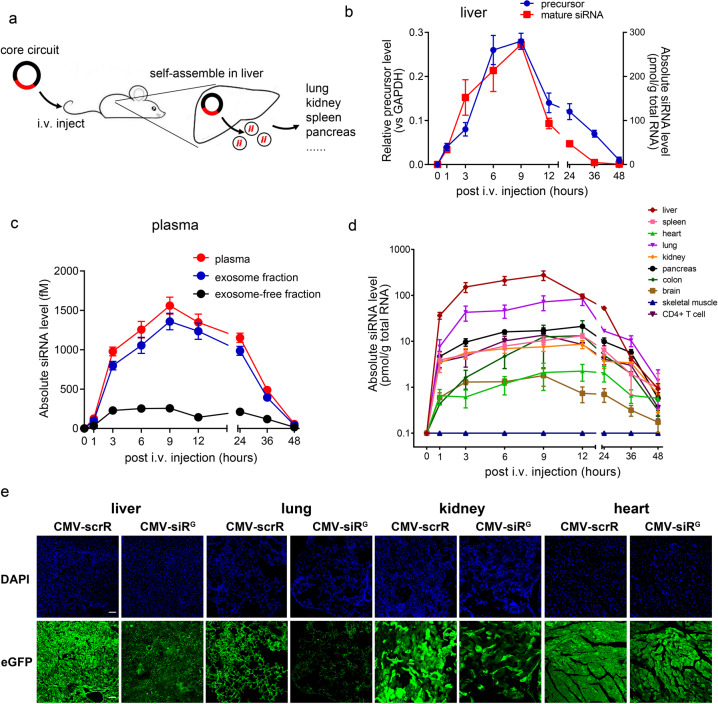
Delivery of EGFR siRNA to various mouse tissues following the intravenous injection of the CMV-siR^E^ circuit. **a** Schematic presentation of the in vivo assembly and delivery of siRNAs by intravenous injection of the core circuit. **b** Kinetics of the precursor and mature form of EGFR siRNA in mouse liver following tail vein injection of 5 mg/kg CMV-siR^E^ circuit (*n* = 3 in each group). **c** Kinetics of the EGFR siRNA in the mouse plasma, exosome pellets and exosome-free plasma following tail vein injection of 5 mg/kg CMV-siR^E^ circuit (*n* = 3 in each group). **d** Tissue distribution kinetics of the EGFR siRNA in various mouse tissues following tail vein injection of 5 mg/kg CMV-siR^E^ circuit (*n* = 3 in each group). **e** Representative fluorescence microscopy images showing eGFP levels in frozen sections of liver, lung, kidney and heart obtained from eGFP-transgenic mice after intravenous injection of 5 mg/kg CMV-scrR or CMV-siR^G^ circuit for seven times. Positive eGFP signals are shown in green, and DAPI-stained nuclei are shown in blue. Scale bar, 100 μm. Values are presented as the means ± SEM.

To ensure that the genetic circuits developed in vitro also work well in the complex in vivo environment, a stable tissue chassis that is exploited to express the siRNA payload, to coordinate siRNA assembly into exosomes and to direct exosome in vivo delivery, should be developed. Because the liver can express transgenes introduced by intravenously injected naked DNA plasmids,^25,26^ we asked whether the liver can be reprogrammed to direct the formation of exosome-encapsulated siRNA after taking up the genetic circuits (Fig. 2a). To test this hypothesis, we determined the tissue distribution of the injected circuits and the desired siRNA “product” in mice after intravenous injection with the CMV-siR^E^ core circuit. First, the in vivo accumulation and clearance of the original circuit was assessed using the clone formation unit (CFU) approach. Circuits purified from tissue or plasma samples were used to transform bacteria and the number of antibiotics-resistant colonies formed in plates could represent the relative abundance of intact genetic circuits present in each sample (Supplementary information, Fig. S7a). The CMV-siR^E^ circuit was found to rapidly accumulate in the liver and then gradually decrease to background level at 168 h (7 days) and was completely cleared from the liver by 720 h (30 days); in plasma, the CMV-siR^E^ circuit gradually decreased to the background until 168 h (7 days); no CMV-siR^E^ circuit was detected in other tissues (Supplementary information, Fig. S7b, c). Moreover, when the absolute level of CMV-siR^E^ circuit in the liver was determined by a quantitative RT-PCR assay, a similar kinetics and distribution pattern was observed (Supplementary information, Fig. S7d, e). Similarly, the precursor of EGFR siRNA was solely detected in the liver but not in other tissues (Fig. 2b and Supplementary information, Fig. S8). Furthermore, when a CMV-eGFP-siR^E^ circuit (coexpressing an eGFP protein and an EGFR siRNA) was used, eGFP fluorescence was also restricted to the liver (Supplementary information, Fig. S9). These results are consistent with previous reports^27,28^ and support the idea that the liver is the major organ taking up exogenous circuits. Second, to confirm that hepatocytes are the site of in vivo siRNA assembly and release, we established an ex vivo model using primary mouse hepatocytes isolated from mice injected with the CMV-siR^E^ circuit. The abundance of EGFR siRNA in mouse hepatocyte exosomes and its association with AGO2 were confirmed (Supplementary information, Fig. S10). Third, we investigated whether self-assembled siRNA enters the circulation by measuring the concentration of EGFR siRNA in the whole plasma, exosome pellets and exosome-free plasma. Exosomes derived from mouse plasma exhibited normal morphology and size distribution, enrichment of exosomal markers (CD63, TSG101 and CD9) and depletion of the plasma marker albumin (Supplementary information, Fig. S11). A time-dependent change in EGFR siRNA levels (peaking at approximately 9 h and decreasing to background levels at 48 h) was observed in the plasma and plasma exosome pellets, but EGFR siRNA was almost completely absent in the exosome-free plasma (Fig. 2c). Fourth, direct measurement of single-stranded mature siRNA in mouse tissues revealed the accumulation of EGFR siRNA in the liver, lung, pancreas, spleen and kidney in a time-dependent manner (peaking after 9–12 h and decreasing to background levels at 48 h); however, only a slight increase in single-stranded mature siRNA was observed in the heart and brain, and none was observed in skeletal muscle (Fig. 2b, d and Supplementary information, Fig. S12). The tissue distribution of EGFR siRNA was also dose-dependent (Supplementary information, Fig. S13) and further confirmed by fluorescence in situ hybridization (FISH) (Supplementary information, Fig. S14). After conversion to absolute levels, approximately 2400 and 120 copies per cell of EGFR siRNA were present in the liver and lung 9 h after a single injection of 5 mg/kg circuits, which reached the theoretical threshold functional level of siRNA in vivo (100 copies per cell).^29^ Fifth, to confirm that the liver is the original site for the self-assembly and release of siRNA, the CMV-siR^E^ circuit was directly injected into the mouse liver via the common bile duct. Approximately the same kinetics and distribution pattern (e.g., restriction of the siRNA precursor to the liver, enrichment of EGFR siRNA in the plasma exosomes and accumulation of EGFR siRNA in various tissues) were observed, except that the peak shifted to an earlier time point (Supplementary information, Fig. S15). Since only the liver has direct access to injected circuits under this condition, the increase in EGFR siRNA in the plasma and tissues again demonstrates the existence of a fully functional hepatic system for in vivo siRNA assembly and delivery. Furthermore, when the CMV promoter was replaced with a liver-specific albumin promoter to construct an albumin-directed circuit encoding EGFR siRNA (Alb-siR^E^), a similar dynamics of siRNA distribution in the plasma was observed compared with the CMV-siR^E^ circuit, despite a lower peak level of plasma siRNA in Alb-siR^E^ circuit-treated mice which might be due to the lower transcription efficiency of the albumin promoter (Supplementary information, Fig. S16). These results validate again that the genetic circuit is indeed transcribed in the liver. Finally, to directly visualize and track the tissues that take up in vivo assembled siRNA, a CMV-directed circuit expressing an eGFP-silencing siRNA (CMV-siR^G^) was intravenously injected into transgenic mice ubiquitously expressing eGFP. Fluorescence levels were significantly reduced in the liver, lung and kidney but not in the heart of the eGFP-transgenic mice (Fig. 2e and Supplementary information, Fig. S17). Taken together, these results suggest that the liver, through spontaneous organization and release of siRNA-containing exosomes, enables the efficient delivery of functional siRNAs to distant tissues.

### Design of the core circuits targeting EGFR-driven lung cancer

**Fig. 3:**
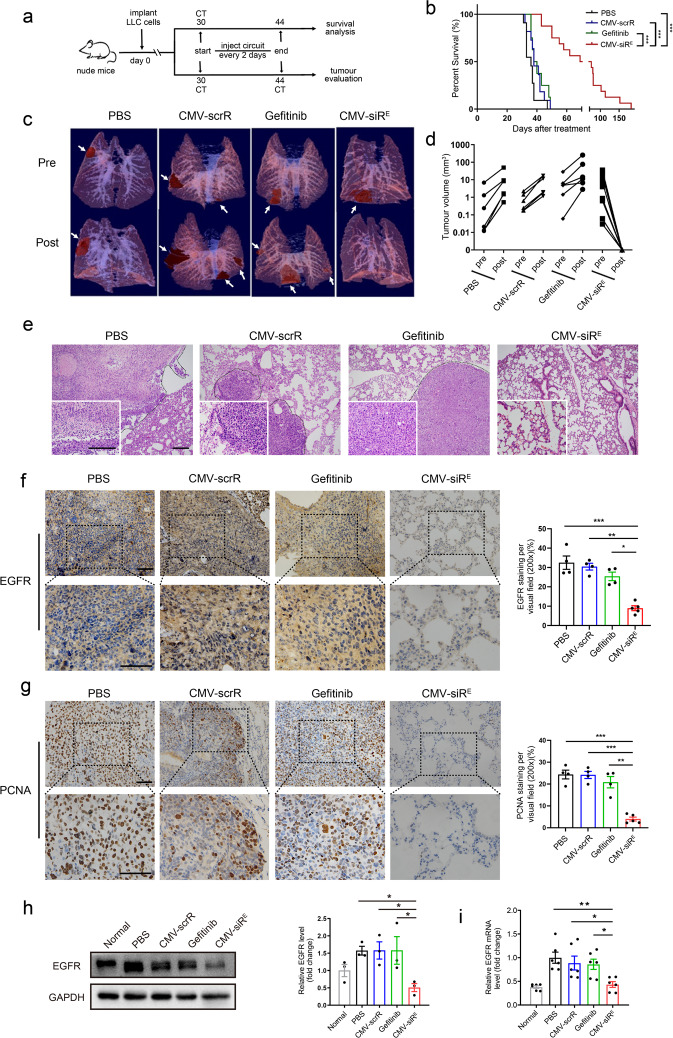
Intravenous injection of the CMV-siR^E^ circuit diminishes tumor formation in orthotopic lung cancer models. **a** Flow chart of the experimental design. Nude mice were intravenously injected with LLC cells and analyzed using micro-CT at 30 days post-inoculation to ensure the formation of lung tumors. Next, mice were intravenously injected with PBS or 5 mg/kg CMV-scrR or CMV-siR^E^ circuit or intragastrically administered gefitinib every 2 days for a total of seven injections. Mice were then monitored to evaluate survival time or tumor growth. **b** Kaplan–Meier survival curves (PBS, *n* = 11; CMV-scrR, *n* = 11; gefitinib, *n* = 8; CMV-siR^E^, *n* = 16). **c** Representative 3-D reconstructions of mouse lungs pre- and post-treatment with genetic circuits or gefitinib. Tumors are shown in maroon to demonstrate their location in the 3-D reconstructions. The entire 3-D reconstructions are shown in Supplementary information, Fig. S19. **d** A semiautomated quantitative image analysis was performed using 3-D reconstructions of the thoracic cavity to assess the tumor volume pre- and post-treatment with genetic circuits or gefitinib (PBS, CMV-scrR and gefitinib, *n* = 6; CMV-siR^E^, *n* = 12). **e** Representative H&E-stained lung sections. Scale bar, 200 μm. **f** Representative EGFR-stained lung sections and quantitative analysis of EGFR levels in lung sections (PBS, CMV-scrR and gefitinib, *n* = 4; CMV-siR^E^, *n* = 5). Scale bar, 75 μm. **g** Representative proliferating cell nuclearantigen (PCNA)-stained lung sections and quantitative analysis of PCNA levels in lung sections (PBS, CMV-scrR and gefitinib, *n* = 4; CMV-siR^E^, *n* = 5). The tumor cell proliferation rate is indicated by the percentage of PCNA-positive cells. Scale bar, 75 μm. **h** Western blot analysis of EGFR protein levels in lung tumor samples. Normal mice without tumors were included as negative controls. Left panel, representative western blots. Right panel, quantitative analysis (*n* = 3 in each group). **i** Quantitative RT-PCR analysis of *EGFR* mRNA levels in lung tumor samples (*n* = 6 in each group). Normal mice without tumors were included as negative controls. Values are presented as the means ± SEM. Significance was determined using one-way ANOVA followed by Dunnett’s multiple comparison in **f**–**i**. Kaplan–Meier survival analyses were performed in **b**, and statistical significance was assessed with the log-rank test. *P < 0.05; **P < 0.01; ***P < 0.005; ns, not significant.

**Fig. 4:**
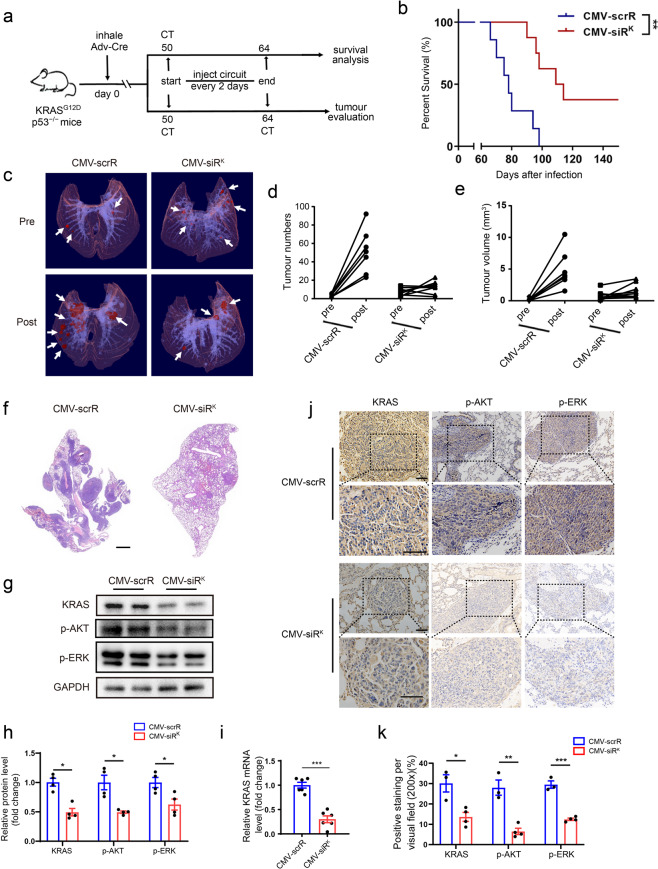
Intravenous injection of the CMV-siR^K^ circuit diminishes tumor formation in spontaneous lung cancer models. **a** Flow chart of the experimental design. The *KRAS*^*LSL-G12D*^*;p53*^*flfl*^ mice were administered Adeno-Cre and analyzed using micro-CT 50 days post-inhalation to ensure spontaneous tumor formation in the lungs. Mice were then intravenously injected with 5 mg/kg CMV-scrR or CMV-siR^K^ circuit every 2 days for a total of seven injections. Mice were then monitored to evaluate survival time or tumor growth. **b** Kaplan–Meier survival curves (CMV-scrR, *n* = 7; CMV-siR^K^, *n* = 8). **c** Representative 3-D reconstructions of mouse lungs pre- and post-treatment with genetic circuits. The entire 3-D reconstructions are shown in Supplementary information, Fig. S22. **d**, **e** A semiautomated quantitative image analysis was performed using 3-D reconstructions of the thoracic cavity to assess the tumor number and volume pre- and post-treatment with genetic circuits (CMV-scrR, *n* = 7; CMV-siR^K^, *n* = 8). **f** Representative H&E-stained lung sections. Scale bar, 1000 μm. **g** Western blot analysis of KRAS, p-AKT and p-ERK protein levels in the lung tumor samples (*n* = 4 in each group). Shown are representative western blots. **h** Quantitation of KRAS, p-AKT and p-ERK protein levels in the lung tumor samples (*n* = 4 in each group). **i** Quantitative RT-PCR analysis of *KRAS* mRNA levels in the lung tumor samples (*n* = 6 in each group). **j** Representative images of immunohistochemical staining of KRAS, p-AKT and p-ERK proteins in lung sections. Scale bar, 75 μm. **k** Quantitative analysis of immunohistochemical staining of KRAS, p-AKT and p-ERK proteins in lung sections (CMV-scrR, *n* = 3; CMV-siR^K^, *n* = 4). Values are presented as the means ± SEM. Significance was determined using two-sided *t*-test in **h**, **i**, **k**. Kaplan–Meier survival analyses were performed in **b**, and statistical significance was assessed with the log-rank test. *P < 0.05; **P < 0.01; ***P < 0.005; ns, not significant.

To evaluate the therapeutic potential of our designed genetic circuits, we first assessed the anticancer effect of the CMV-siR^E^ core circuit in a mouse orthotopic lung cancer model induced by LLC cells (Fig. 3a). Gefitinib, an EGFR tyrosine kinase inhibitor to which LLC cells are insensitive,^30^ was used as a negative control. Compared to mice treated with PBS, gefitinib or CMV-scrR, mice treated with the CMV-siR^E^ circuit showed (1) a markedly increased overall survival rate (Fig. 3b); (2) an almost completely diminished lung tumor burden (Fig. 3c, d and Supplementary information, Figs. S18, S19); (3) largely reversed lung tumor histology (Fig. 3e); (4) a significant decrease in EGFR protein and mRNA to nearly normal levels in lung tumors (Fig. 3f, h, i); and (5) a significantly decreased tumor cell proliferation rate (Fig. 3g). Potential side effects and tissue toxicity of the circuits were also evaluated. Repeated injection of the CMV-siR^E^ circuit posed negligible hepatic toxicity or renal toxicity, no noticeable irregularities and overt tissue damages, no significant reduction of lymphoid organ weights, no alteration of peripheral immune cell counts and no significant alteration of inflammatory cytokines (Supplementary information, Fig. S20). These results indicate that the genetic circuits are biocompatible, non-toxic and non-immunogenic.

### Design of the core circuits targeting KRAS-driven lung cancer

To further validate the universality and flexibility of the core circuit design, we replaced the EGFR siRNA “payload” with an siRNA sequence targeting Kirsten rat sarcoma viral oncogene homologue (*KRAS*), an oncogene that is notoriously difficult for targeting pharmacologically in the clinic.^31^ We constructed a CMV-siR^K^ circuit and investigated its therapeutic effect in a *KRAS*^*LSL-G12D*^*; p53*^*fl/fl*^ transgenic mouse model that develops spontaneous, sporadic focal pulmonary adenocarcinomas following intranasal inoculation of Cre-containing adenovirus (Adeno-Cre) to activate the latent oncogenic *Kras*^*G12D*^ allele. Approximately 50 days after Adeno-Cre administration, pulmonary adenocarcinoma formation was verified by micro-CT scanning. Tumor-bearing mice were then intravenously injected 7 times with the control CMV-scrR circuit or the CMV-siR^K^ circuit (Fig. 4a). A significant survival advantage was observed in the CMV-siR^K^-treated group (Fig. 4b). Micro-CT scans and 3-D reconstruction showed a significant reduction in both tumor number and volume in the CMV-siR^K^-treated group (Fig. 4c–e and Supplementary information, Figs. S21, S22), which was also confirmed by histology (Fig. 4f). At the molecular level, significant amounts of KRAS siRNA were detected in the plasma and plasma exosome pellets 9 h after the final injection (Supplementary information, Fig. S23a). Consequently, a decrease in KRAS protein and mRNA levels and a concomitant decrease in AKT and ERK phosphorylation levels (p-AKT and p-ERK are direct downstream effectors of the KRAS signal pathway) were observed in lung tumors from mice injected with the CMV-siR^K^ circuit (Fig. 4g–i), which was also confirmed by immunohistochemical staining (Fig. 4j, k). Likewise, suppressed cell proliferation (fewer PCNA-positive cells) and enhanced cell apoptosis (more TUNEL-positive cells) were noted in lung tumors from the CMV-siR^K^-treated group (Supplementary information, Fig. S23b). Overall, these results indicate that intravenous injection of the core circuit effectively induced functional siRNA in vivo, and this approach may serve as a potential therapeutic regimen for lung cancer.

### Design of the brain-directed composable-core circuits targeting glioblastoma

**Fig. 5:**
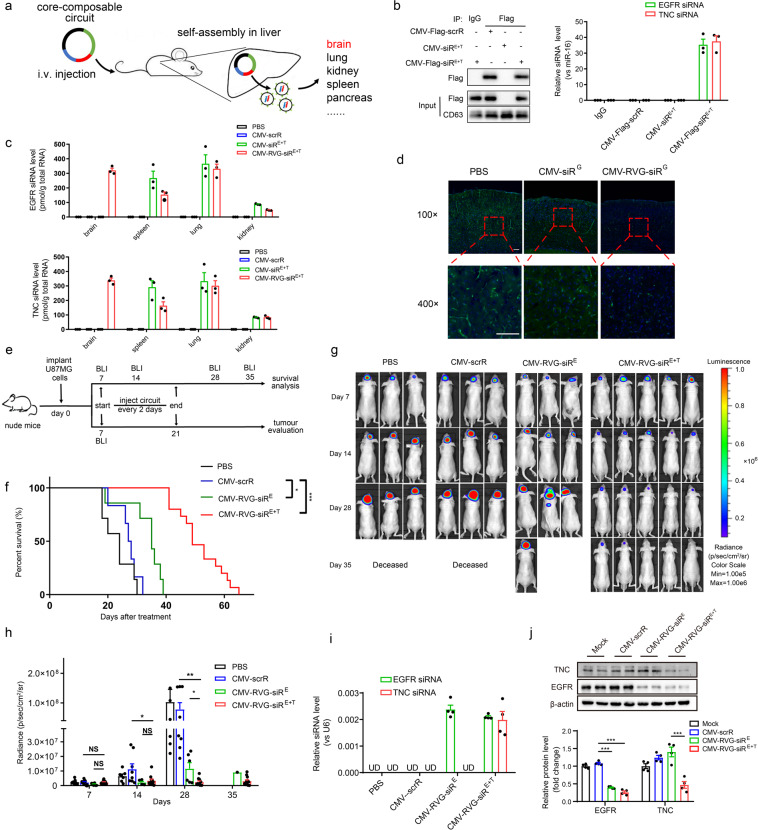
Intravenous injection of the CMV-RVG-siR^E+T^ circuit delivers siRNAs to the brain and inhibits tumor growth in a glioblastoma mouse model. **a** Schematic presentation of the in vivo assembly and delivery of siRNA by intravenous injection of the composable-core circuit. **b** The CMV-Flag-scrR, CMV-siR^E+T^ or CMV-Flag-siR^E+T^ circuits were intravenously injected into mice. Plasma exosomes were then either directly loaded for western blotting with anti-Flag and anti-CD63 antibodies (middle and bottom lanes) or immunoprecipitated with IgG or anti-Flag beads before western blotting (upper lane). A quantitative RT-PCR assay was performed to assess the levels of EGFR and TNC siRNAs in immunoprecipitated exosomes (*n* = 3 in each group). **c** The levels of EGFR and TNC siRNAs in mouse brain, spleen, lung and kidney tissues after intravenous injection of PBS or 5 mg/kg CMV-scrR, CMV-siR^E+T^ or CMV-RVG-siR^E+T^ circuit (*n* = 3 in each group). **d** Representative fluorescence microscopy images showing eGFP levels in frozen sections of brain obtained from eGFP-transgenic mice after intravenous injection of PBS, 5 mg/kg CMV-siR^G^ circuit or CMV-RVG-siR^G^ circuit for seven times. Positive eGFP signals are shown in green, and DAPI-stained nuclei are shown in blue. Scale bar, 100 μm. **e** Flow chart of the experimental design. Nude mice were intracranially implanted with bioluminescent U87MG-Luc cells and analyzed using BLI on day 7 post-implantation to ensure glioblastoma formation in the brain. Mice were then intravenously injected with PBS or 5 mg/kg CMV-scrR, CMV-RVG-siR^E^ or CMV-RVG-siR^E+T^ circuit for a total of seven times over 2 weeks. Mice were then either monitored for survival analysis or sacrificed for evaluation of tumor growth. **f** Kaplan–Meier survival curves (PBS, *n* = 7; CMV-scrR, *n* = 6; CMV-RVG-siR^E^, *n* = 7; CMV-RVG-siR^E+T^, *n* = 15). **g** BLI images of glioblastoma sizes in representative mice. **h** Quantification of glioblastoma sizes (PBS, *n* = 7; CMV-scrR, *n* = 8; CMV-RVG-siR^E^, *n* = 6; CMV-RVG-siR^E+T^, *n* = 11). **i** The levels of EGFR and TNC siRNAs in glioblastoma samples (*n* = 4 in each group). **j** Western blot analysis of EGFR and TNC protein levels in glioblastoma samples. Upper panel, representative western blots. lower panel, quantitative analysis (*n* = 4 in each group). Values are presented as the means ± SEM. Significance was determined using one-way ANOVA followed by Dunnett’s multiple comparison in **h**, **j**. Kaplan–Meier survival analyses were performed in **f**, and statistical significance was assessed with the log-rank test. *P < 0.05; **P < 0.01; ***P < 0.005; ns, not significant.

To validate the tissue-targeting ability of our designed composable-core circuits, we investigated whether incorporating brain-specific guidance tags into the core circuit could facilitate siRNA delivery to the brain (Fig. 5a). When the CMV-Flag-siR^E+T^ circuit was intravenously injected into mice, plasma exosomes were effectively immunoprecipitated with anti-Flag beads, and the enrichment of EGFR and TNC siRNAs in the immunoprecipitated exosomes was also observed (Fig. 5b). When the RVG tag was used (CMV-RVG-siR^E+T^), EGFR and TNC siRNAs accumulated in the brain, whereas no signal was detected in the brains of mice injected with the control CMV-siR^E+T^ circuit (Fig. 5c). To directly visualize the distribution of RVG-guided siRNA, we intravenously injected a CMV-RVG-siR^G^ circuit into eGFP-transgenic mice. Efficient reduction in eGFP levels was observed in the brain (Fig. 5d and Supplementary information, Fig. S24). Likewise, when C57BL/6J mice were injected with a circuit expressing an RVG-Lamp2b-eGFP triple fusion protein (RVG and eGFP were fused to the N- and C-termini of Lamp2b, respectively) to generate eGFP-positive, brain-directed exosomes, the eGFP signal was readily detected in the mouse brain (Supplementary information, Fig. S25). The above results demonstrate that the composable-core circuit enables assembly of siRNAs and delivery of them to the brain, successfully bypassing the blood-brain barrier.

Next, we established an orthotopic mouse model of glioblastoma by intracranially implanting bioluminescent U87MG-Luc cells into nude mice. After intravenously injecting PBS or the CMV-scrR, CMV-RVG-siR^E^ or CMV-RVG-siR^E+T^ circuit into glioblastoma-bearing nude mice for 7 times, mice were either followed-up for survival analysis or scanned using bioluminescence imaging (BLI) to evaluate tumor growth (Fig. 5e). Overall survival was modestly improved in mice treated with the CMV-RVG-siR^E^ circuit, while a dramatic survival advantage was observed in mice treated with the CMV-RVG-siR^E+T^ circuit (Fig. 5f). Extensive brain tumor burden and accelerated tumor growth were observed in mice treated with PBS or the control circuit, whereas tumor growth was significantly decelerated in the CMV-RVG-siR^E^-treated mice (Fig. 5g, h). Strikingly, tumors in the CMV-RVG-siR^E+T^ group were reduced to nearly undetectable levels, and this effect persisted throughout the experimental period (Fig. 5g, h). At the molecular level, EGFR siRNA was abundantly present in glioblastomas of mice treated with the CMV-RVG-siR^E^ or CMV-RVG-siR^E+T^ circuit (Fig. 5i), whereas EGFR protein levels were significantly reduced (Fig. 5j and Supplementary information, Fig. S26). Likewise, TNC siRNA levels were increased, and protein levels were reduced in glioblastomas of mice treated with the CMV-RVG-siR^E+T^ circuit (Fig. 5i, j and Supplementary information, Fig. S26). As a result, suppressed cell proliferation (fewer PCNA-positive cells) was observed in glioblastomas of mice treated with the CMV-RVG-siR^E^ or CMV-RVG-siR^E+T^ circuit (Supplementary information, Fig. S26). Collectively, these results prove that composable-core circuits containing tissue-targeting parts and multiple siRNA expression parts are functional in vivo and may be useful as an organ-directed delivery platform for siRNA therapeutics.

### Design of the brain-directed composable-core circuits targeting obesity

**Fig. 6:**
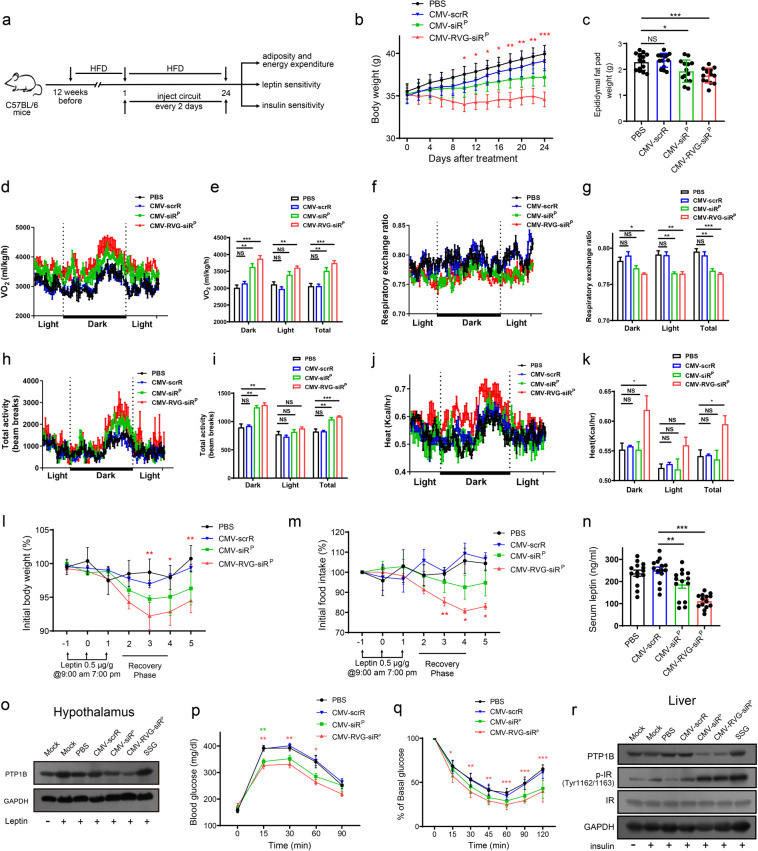
Intravenous injection of the CMV-RVG-siR^P^ circuit restores leptin and insulin sensitivity, decreases adiposity and increases energy expenditure in a mouse model of obesity. **a** Flow chart of the experimental design. Male C57BL/6J mice at 3 weeks of age were placed on a HFD for 12 weeks. Mice rapidly gained weight and became obese. Mice were then maintained on a HFD and treated with PBS or 5 mg/kg CMV-scrR, CMV-siR^P^ or CMV-RVG-siR^P^ circuit through tail vein injection for a total of 12 times over 24 days. Body weights were monitored during treatment. After treatment, mice were divided into several groups and subjected to the evaluation of fat mass, energy expenditure, leptin sensitivity and glucose homoeostasis. **b** Body weight curves (*n* = 14 in each group). **c** Weights of epididymal fat pads (*n* = 14 in each group). **d**–**k** Energy expenditure parameters, including oxygen consumption (VO_2_), respiratory exchange ratio (RER), total activity and heat production, were monitored (*n* = 3 in each group). **l**, **m** Weight loss and food intake inhibition in response to leptin. Male mice were injected with leptin (0.5 μg/g body weight every 12 h) for the indicated periods. Food intake and body weight were monitored 2 days prior to the start of injection and normalized to 100% for day 0 values (*n* = 6 in each group). **n** Basal serum leptin levels (*n* = 6 in each group). **o** Western blot analysis of PTP1B in the hypothalamus. Mice were injected with or without leptin (to stimulate leptin signalling), and hypothalamus was collected for immunoblot analysis of PTP1B (*n* = 3 in each group). SSG (200 mg/kg i.p. dose) serves as a control. Shown are representative western blots. **p** Mouse GTT results (*n* = 8 in each group). **q** Mouse ITT results. Blood glucose values are expressed as the percentage of the initial concentration (*n* = 8 in each group). **r** Western blot analysis of PTP1B and tyrosine phosphorylation of insulin receptors in the liver. Mice were injected with or without insulin (to stimulate insulin signalling), and liver was collected for immunoblot analysis with antibodies against PTP1B, p-IR (Tyr1162/Tyr1163) or total IR (*n* = 3 in each group). SSG (200 mg/kg i.p. dose) serves as a control. Shown are representative western blots. Values are presented as the means ± SEM. Significance was determined using one-way ANOVA followed by Dunnett’s multiple comparison in **c**, **e**, **g**, **i**, **k**, **n**, and using two-way ANOVA followed by Dunnett’s multiple comparison in **b**, **l**, **m**, **p**, **q**. *P < 0.05; **P < 0.01; ***P < 0.005; ns, not significant.

The brain-directed composable-core circuits may also provide a therapeutic option for obesity because the central nervous system, especially the hypothalamus, plays a key role in controlling energy homoeostasis.^32^ Leptin, an adipocyte-secreted hormone, acts on the hypothalamus to suppress food intake and increase energy expenditure,^33,34^ and insulin, a key regulator of glucose homoeostasis, acts on insulin receptors in the hypothalamic arcuate nucleus to regulate hepatic glucose production via the vagus nerve.^35^ Since most obese individuals develop leptin and insulin resistance,^36^ targeting genes involved in the development of leptin and insulin resistance may represent an ideal strategy to overcome the obesity epidemic. One promising target is protein tyrosine phosphatase 1B (PTP1B), a critical negative regulator of leptin and insulin signalling in hypothalamic neurons.^37–39^ Neuronal PTP1B-deficient mice are hypersensitive to leptin and insulin, have reduced body weight and adiposity, and exhibit improved glucose homoeostasis and increased energy expenditure.^40,41^ Since hypothalamic PTP1B lies at the heart of leptin and insulin signalling, we asked whether brain-directed circuits targeting PTP1B could function as a two-pronged approach to overcome both leptin and insulin resistance in obese individuals. First, we assessed whether PTP1B siRNA is delivered to the hypothalamus. Circuits encoding PTP1B siRNA with or without an RVG tag (CMV-RVG-siR^P^ or CMV-siR^P^) were intravenously injected into mice. While FISH assay revealed hybridization signals of PTP1B siRNA in the liver of mice injected with either CMV-RVG-siR^P^ or CMV-siR^P^ circuit, accumulation of PTP1B siRNA was only detected in the hypothalamus of mice injected with the CMV-RVG-siR^P^ circuit (Supplementary information, Fig. S27a, b), confirming the importance of the RVG tag for the delivery of siRNA to the hypothalamus. Accordingly, using immunofluorescence staining, we detected a significant decrease in PTP1B protein signal only in the hypothalamus of mice injected with the CMV-RVG-siR^P^ circuit (Supplementary information, Fig. S27c). Next, we investigated whether the circuits reduce adiposity in a high-fat diet (HFD)-induced mouse model of obesity. Obese mice maintained on an HFD were intravenously injected 12 times with PBS or CMV-scrR, CMV-siR^P^ or CMV-RVG-siR^P^ circuit (Fig. 6a). Compared with the PBS, CMV-scrR and CMV-siR^P^ groups, the CMV-RVG-siR^P^ group was substantially protected from HFD-induced weight gain (Fig. 6b). Adiposity was also decreased in the CMV-RVG-siR^P^ group, as evidenced by reduced epididymal fat pad weight (Fig. 6c) and reduced serum triglyceride, total cholesterol and low-density lipoprotein levels (Supplementary information, Fig. S28a–c). Since no significant difference was observed in body length and food intake among all groups (Supplementary information, Fig. S28d, e), we assessed the difference in energy expenditure by an open-circuit indirect calorimetry system. Compared with the PBS and CMV-scrR groups, the CMV-RVG-siR^P^ group showed an increase in oxygen consumption (Fig. 6d, e) and a decrease in respiratory exchange ratio (Fig. 6f, g) during both the light and dark cycles, indicating preferential consumption of fat rather than carbohydrate as an energy source. Moreover, the CMV-RVG-siR^P^ group had significantly elevated locomotor activity (Fig. 6h, i) and heat production (Fig. 6j, k) levels, particularly during the dark cycle, suggesting that the CMV-RVG-siR^P^ circuit causes an increase in energy expenditure in obese mice, promoting the dissipation of excess energy as heat rather than the storage of it as fat. To examine whether the increased energy expenditure is due to enhanced leptin sensitivity, we administered leptin peripherally into obese mice and monitored both weight change and food intake. While the PBS, CMV-scrR and CMV-siR^P^ groups did not respond to leptin, the CMV-RVG-siR^P^ group showed a clear decrease in body weight and suppression of food intake in response to leptin (Fig. 6l, m). Accordingly, the CMV-RVG-siR^P^ group had significantly reduced serum leptin levels (Fig. 6n). At the molecular level, while liver PTP1B protein levels were reduced in both CMV-siR^P^ and CMV-RVG-siR^P^ group, hypothalamic PTP1B protein level was only reduced in CMV-RVG-siR^P^ group (Fig. 6o, r and Supplementary information, Fig. S28f). Moreover, the CMV-RVG-siR^P^ circuit was also more potent in reducing hypothalamic PTP1B levels than sodium stibogluconate (SSG), a PTP1B inhibitor that has low permeability of the blood-brain barrier and can only reduce body weight at a high dose (e.g., 200 mg/kg i.p. dose), further demonstrating the advantage of in vivo self-assembled siRNA.

Next, we evaluated the effect of the genetic circuits on insulin signalling. Insulin controls glucose homoeostasis through both a direct action on insulin receptors of peripheral tissues (e.g., liver and muscle) and an indirect action on insulin receptors in the central nervous system.^35^ While neuronal PTP1B-deficient mice are lean and insulin hypersensitive, deficiency of PTP1B in liver only improves insulin sensitivity but has little effect on body weight and adiposity.^42,43^ Consistent with this notion, the CMV-siR^P^ group displayed significantly enhanced glucose clearance from peripheral circulation in the glucose tolerance test (GTT; Fig. 6p), a decrease in blood glucose in the insulin tolerance test (ITT; Fig. 6q) and reduced basal serum insulin concentration (Supplementary information, Fig. S29a), but had only a slight loss of body weight and fat (Fig. 6b, c). In contrast, since the CMV-RVG-siR^P^ circuit delivered a significant amount of PTP1B siRNA to both the liver and hypothalamus, which may generate a synergistic effect on central and peripheral insulin signalling, insulin sensitivity and glucose tolerance were improved to the greatest degree in the CMV-RVG-siR^P^ group (Fig. 6p, q and Supplementary information, Fig. S29a). Likewise, both CMV-siR^P^ and CMV-RVG-siR^P^ groups displayed decreased PTP1B expression in the liver and increased phosphorylation of insulin receptors in the liver (Fig. 6r and Supplementary information, Fig. S28f), which was accompanied by a reduction in glucose and lipid synthesis genes in the liver (Supplementary information, Fig. S29b) and an alleviation of fatty liver as indicated by a remarkable decline in lipid droplet accumulation in the liver (Supplementary information, Fig. S29c). To further clarify the effect of circuit-induced PTP1B knockdown on insulin sensitivity, leptin-deficient *ob/ob* mice were intravenously injected 12 times with PBS or CMV-scrR, CMV-siR^P^ or CMV-RVG-siR^P^ circuit. While body weight and fat weight were similar among the 4 groups (Supplementary information, Fig. S30a, b), insulin sensitivity and glucose homoeostasis were significantly improved in the CMV-siR^P^ and CMV-RVG-siR^P^ groups, especially the latter (Supplementary information, Fig. S30c, d). Accordingly, fatty liver was also improved to the greatest degree in the CMV-RVG-siR^P^ group (Supplementary information, Fig. S30e). Overall, these results suggest that the inhibition of central and peripheral PTP1B using genetic circuits may overcome leptin and insulin resistance and provide an attractive strategy to combat obesity.

## Discussion

Most human diseases are driven by the mutation and/or dysfunction of a certain set of genes/proteins. Although numerous small molecules and antibodies have been developed to specifically target these proteins, the applicability of current targeted therapies is still limited to a small fraction (< 1%) of the human proteome.^44^ siRNAs offer an opportunity to specifically target mRNAs and modulate the expression of corresponding proteins before their biogenesis,^3,45^ and have therefore greatly enlarged the proportion of human proteins that can be therapeutically manipulated.^46^ The common consensus that the major challenge for RNAi-based therapy is the lack of an efficient in vivo delivery system,^47^ had elicited tremendous efforts to overcome this hurdle, including using delivery vehicles and conjugated ligands. These techniques have yielded considerable advances in pre-clinical studies and in clinical trials, with some recent approval by the FDA. In this study, we reprogrammed the native circulating exosome system of mammals with artificial genetic circuits to facilitate the transfer of siRNA in vivo. This strategy has several inherent advantages over conventional siRNA delivery systems. (1) Safety. We re-conceptualized delivery vehicles as “medicines” instead of “agents”, thus avoiding the safety concerns associated with conventional delivery techniques. The first human clinical trial of a nanoparticle-delivered siRNA (CALAA-01) to treat solid tumors produced serious adverse effects caused by components of the delivery system rather than by the siRNA itself;^48^ similarly, a fatality case reported in one clinical trial has caused a significant roadblock to the implementation of viral vehicles.^49^ A recent study also reported fatal cardiotoxicity associated with the otherwise successful AAV-mediated miRNA therapy for myocardial infarction,^50^ emphasizing the vital importance of tightly controlling the delivery vehicle and dosage of RNAi therapies. In our study, siRNAs are produced and assembled by the host liver and are delivered by the endogenous exosome transport machinery; therefore, our strategy is biocompatible, non-toxic and non-immunogenic. Furthermore, the pre-miRNA backbone was optimized to eliminate the passenger strand, thus preventing the toxicity and unintended off-target effects caused by the improper selection of the passenger strand into the RNAi pathway. Additionally, our system produces siRNAs in a time- and dose-dependent manner; therefore, the efficacy of the system is predictable and well controlled. (2) Efficiency. Our strategy induced efficient siRNA accumulation and potent target gene silencing, despite the fact that in vivo self-assembled siRNAs were present at a much lower concentration in tissues and blood than exogenously introduced siRNAs that are pre-assembled in vitro.^9,10,14^ In the case of exogenous siRNAs that are loaded into artificial nanoparticles or cell-derived exosomes in vitro, these siRNAs have not undergone the natural processes including maturation of siRNA by strand separation, loading of guide siRNA onto AGO2 and forming the functional center of an RISC, which could significantly diminish the bioactivity of the siRNAs. Previous studies have also pointed out that in vitro loading of naked siRNAs into exosomes by electroporation could cause extensive siRNA aggregation and significantly reduce the amount of bioactive siRNAs.^51^ Unlike in vitro pre-assembled siRNAs that are usually not effectively integrated into the endogenous RNAi system, in vivo self-assembled siRNAs are bound tightly to AGO2 and enriched in exosomes, which not only protect them from premature degradation and maintain their stability in circulation, but also facilitate their cellular uptake, intra-cytoplasmic release and lysosomal escape in recipient cells, thereby ensuring maximal incorporation of intact siRNAs into the functional RNAi pathway of target cells. This could explain why in vivo self-assembled siRNAs present at low plasma concentration can still have sufficient biological effects. In fact, according to our calculations, in vivo self-assembled siRNAs could reach a plasma concentration of approximately 1500 fM, which is similar to previously reported concentration of endogenous exosomal miRNAs that were biologically functional in vivo;^10,52^ when converted to copies per cell, siRNA levels in the liver and lung reached approximately 2400 and 120 copies per cell, respectively, providing a conceptually functional level of endogenous siRNA in vivo (> 100 copies per cell).^29^ (3) Convenience. Our design borrows the body’s workshop and reprogrammes host systems to perform novel and programmable behaviours. A simple injection of the genetic circuits leads to the automatic production and self-assembly of siRNAs into circulating exosomes, which are spontaneously transferred through the naturally existing small RNA transportation machinery to the target cells. Our strategy avoids the complicated procedures, high cost and labour intensity associated with current siRNA delivery techniques. Our strategy (200 μL tail vein injection in 3 s for each mouse) also avoids the invasive hydrodynamics-based injection procedure that was operated by rapidly injecting a large volume of plasmid (1.6 mL in 5 s for each mouse).^53^ (4) Immunocompatability. Since our system utilized the host liver as the biogenerator for in vivo siRNA self-assembly, we expect little to none interaction with the immune system. To prove this, we compared the target silencing efficiency of the genetic circuits in both immunodeficient nude mice and immunocompetent wide-type mice. The CMV-siR^E^ and CMV-RVG-siR^E+T^ circuits effectively inhibited their target *EGFR* gene in both nude and wide-type mice to a similar extent (Supplementary information, Fig. S31), indicating that the immune system has little impact on the generation of self-assembled siRNAs in vivo.

We designed two types of genetic circuits, the core circuit for systematic delivery of siRNA and the composable-core circuit for targeted delivery of siRNA to a specific tissue. The former is best suited for treatment of systematic diseases such as cancers and infectious diseases, since tumor cells and viruses usually circulate in the whole body and systematic clearance is desirable. However, the lack of tissue specificity of the systemically delivered exosomal siRNAs might also elicit safety concerns, which may have a lower impact when targeting the classical oncogenes but might have a side effect on the housekeeping genes involved in regulation of basic cellular functions. According to the “oncogene addiction” theory, although tumor cells have many genetic and epigenetic abnormalities, they usually exhibit dependence on a single oncogenic pathway or protein (e.g., EGFR and KRAS) for their sustained proliferation and survival.^54,55^ Therefore, blockage of only one or a few of these oncogenic proteins can profoundly inhibit the growth of tumor cells and lead to improvements in patient survival, whereas inactivating the normal counterparts of such oncoproteins in normal tissues is often tolerated without obvious consequence.^54^ Consistent with this concept, in the tumor models driven by EGFR or KRAS mutations, while sustained survival and proliferative capacity of tumor cells was dramatically restrained by the CMV-siR^E^ and CMV-siR^K^ circuits, little to none side effects were observed in normal tissues including the liver, lung, kidney pancreas and spleen, where high concentrations of siRNAs were present and significant target gene knockdown was observed (Supplementary information, Fig. S32). Overall, the proper choice of target gene is critical for the efficacy and safety of systemically delivered exosomal siRNAs. The design of core circuits should be optimized to produce siRNAs that target the genes upon which malignant cells have become dependent, therefore inducing devastating effects on malignant cells while sparing normal cells. On the other hand, the composable-core circuits were designed to deliver therapeutic siRNA to the particular site of interest. This concept was validated by the successful silencing of disease genes in tissues not accessible to conventional drugs (e.g., *EGFR*, *TNC* and *PTP1B* in the brain). However, the tissue specificity of the endogenous exosomes is limited by the specificity of the tissue targeting peptides on the outer membrane of the exosomes. Apparently, the currently available tissue-guiding peptides are far from ideal. Moreover, since the host liver is the site of exosomal siRNA production and assembly, accumulation of siRNA in the liver is inevitable, which was observed in both the glioblastoma and obesity models. Under certain circumstances like obesity this could be an advantage, since PTP1B is known to regulate insulin signalling both in the central and peripheral systems, the CMV-RVG-siR^P^ circuit could therefore restore insulin sensitivity to the maximum extent. Nevertheless, because the design of genetic circuits is composable and allows the free combination of different functional modules (e.g., switchable siRNA sequences and plug-and-play parts), our toolbox would enable self-assembled siRNAs to synergistically target multiple genes, cell populations and tissues, even if these genes and tissues are difficult to target with current techniques. Thus, our system may provide a personalized treatment strategy that can address a broad range of problems in biomedicine.

In summary, our strategy induces controllable and predictable self-assembly and delivery of siRNAs in the heterogeneous, dynamic in vivo environment and allows precise control of gene expression in a purpose-driven mode. This state-of-the-art technology has major theoretical significance and translational value because it provides a feasible strategy to overcome the paramount barrier to the therapeutic application of RNAi in vivo, thus representing a new generation of RNAi therapeutics for a variety of diseases ranging from cancers to metabolic diseases.

## Materials and Methods

### Design and construction of genetic circuits

The CMV-siR^E^ circuit was generated by inserting an EGFR siRNA sequence (5′-TGTGGCTTCTCTTAACTCCT-3′) into a 166-bp pre-miR-155 backbone (5′-GGATCCTGGAGGCTTGCTGAAGGCTGTATGCTGAATTCTGTGGCTTCTCTTAACTCCTGTTTTGGCCACTGACTGACAGGAGTTAAGAAGCCACAACCGGTCAGGACACAAGGCCTGTTACTAGCACTCACATGGAACAAATGGCCCAGATCTGGCCGCACTCGAG-3′). The CMV-siR^K^ circuit was generated by inserting a KRAS siRNA sequence (5′-GCAAATACACAAAGAAAGCCC-3′) using the same approach. The CMV-RVG-siR^E+T^ and CMV-Flag-siR^E+T^ circuits that coexpress EGFR and TNC siRNAs along with a guidance tag (RVG or Flag) were generated by the fusion of RVG or Flag tags to the extra-exosomal N-terminus of Lamp2b.^14^ The fused tag-Lamp2b and two tandem pre-miR-155 backbones carrying the EGFR siRNA sequence (5′-TGTGGCTTCTCTTAACTCCT-3′) and the TNC siRNA sequence (5′-CACACAAGCCATCTACACATG-3′) were then cloned downstream of the CMV promoter, resulting in the generation of a circuit that simultaneously encodes EGFR and TNC siRNAs and the tag-Lamp2b fusion protein. The CMV-RVG-siR^P^ circuit that coexpresses a PTP1B siRNA along with a guiding RVG tag was generated by inserting the PTP1B siRNA sequence (5′-CTAACTTCAGTGTCTGGACTC-3′) using the same approach. A circuit designed to express a scrambled RNA was used as the negative control.

Genetic circuits were present in the form of DNA plasmids and were dissolved in PBS without additional formulations. For in vitro cell transfection, genetic circuits (plasmids) were transfected into cultured cells using the Lipofectamine 2000 reagent (Invitrogen, 11668019, MA, USA) according to the manufacturer’s instructions. For in vivo animal experiments, genetic circuits (plasmids) were formed as naked DNA plasmids and administered into mice through regular tail vein injection (200 μL of solution was injected in 3 s) rather than hydrodynamic injection (~1.6 mL of solution was injected in 5 s).

The plasmids used to express genetic circuits were synthesized and constructed by Realgene Biotech Company (Nanjing, China). Maps and scaffolds of the plasmids are shown in Supplementary information, Fig. S33. The plasmids were transformed into *E.coli* DH5α competent cells (Tsingke, TSC01, Beijing, China), cultured with LB medium (with 50 µg/mL spectinomycin) for 14 h in a 37 °C shaking incubator and extracted and purified with EndoFree Maxi Plasmid Kit V2 (Tiangen, DP120, Beijing, China) according to the manufacturer’s instructions. The purified plasmids were sequenced to ensure that the sequences of inserted genetic circuits were correct.

### Cell culture

The mouse lung cancer cell line LLC, human glioblastoma cell line U87MG and human embryonic kidney cell line HEK293T were purchased from the Shanghai Institute of Cell Biology, Chinese Academy of Sciences (Shanghai, China). Cells were cultured in high-glucose (4.5 g/L) DMEM (Gibco, 10564011, MA, USA) supplemented with 10% foetal bovine serum (FBS, Gibco, 10099141C, MA, USA), penicillin and streptomycin in a 5% CO_2_, water-saturated atmosphere.

### Quantitative RT-PCR assay

Total RNA was extracted from cultured cells or mouse tissues using TRIzol Reagent (Invitrogen, Carlsbad, CA) according to the manufacturer’s instructions. Mature siRNAs were quantified by assays using customized TaqMan miRNA probes (Applied Biosystems, CA, USA) according to the manufacturer’s instructions. Briefly, 0.5 µg of total RNA was reverse-transcribed to cDNA using TaqMan MicroRNA Reverse Transcription Kit (Applied Biosystems, 4366596, CA, USA) and a customized stem-loop RT primer (Applied Biosystems, CA, USA). The following reaction conditions were used: 16 °C for 30 min, 42 °C for 30 min, and 85 °C for 5 min. Real-time PCR was performed using a TaqMan™ MicroRNA Assay (Applied Biosystems, 4440887, CA, USA) and a LightCycler96 System (Roche, IN, USA). The reactions were incubated in a 96-well optical plate at 95 °C for 10 min followed by 40 cycles at 95 °C for 15 s and 60 °C for 1 min. All reactions were run in triplicate. After the reactions were complete, the cycle threshold (C_T_) values were determined with the LightCycler96 software, and the mean C_T_ was determined from PCRs performed in triplicate.

For quantification of the absolute levels of siRNAs (EGFR siRNA as an example), synthetic single-stranded EGFR siRNA was serially diluted and assessed using quantitative RT-PCR to generate a standard curve, and a no-template control was assessed simultaneously to determine the specificity of the primer set. Synthetic EGFR siRNA was consistently and efficiently amplified at C_T_ values ranging from 10.59 to 36.90, while the no-template control was not adequately amplified (C_T_ value > 40). According to the dynamic quantification range of the standard curve, the lower boundary of the detection spectrum for EGFR siRNA is 0.1 attomole (corresponding to the C_T_ value of 40). By referring to the standard curve, the concentration of EGFR siRNA in various tissues was calculated and present as the absolute amounts of EGFR siRNA in 1 g of total RNA isolated from each mouse tissue (pmol/g total RNA). For quantification of the relative amounts of siRNA, the siRNA levels in the cells and tissues were normalized to U6 snRNA, while in plasma and exosomes the siRNA levels were normalized to endogenous miR-16.

mRNA was reverse transcribed with AMV Reverse Transcriptase (TaKaRa, 2621, Dalian, China) and oligo-d(T) primers (TaKaRa, 3806, Dalian, China) following total RNA purification with TRIzol (Invitrogen, 15596018), according to the manufacturer’s instructions. Briefly, 1 µg of total RNA was reverse transcribed into cDNA using oligo-d(T) and the AMV reverse transcriptase with the following conditions: 16 °C for 15 min, 42 °C for 60 min, and 85 °C for 5 min. Next, real-time PCR was performed with the RT product, Evagreen Dye (Biotium, 31000, CA, USA) and specific primers for EGFR (forward: 5′-GCATCATGGGAGAGAACAACA-3′; reverse: 5′-CTGCCATTGAACGTACCCAGA-3′), KRAS (forward: 5′-ACTTGTGGTGGTTGGAGCAGC-3′; reverse: 5′-TAGGGTCATACTCATCCACAA-3′), TNC (forward: 5′-ACGAGGGTGGTCTGGAAATG-3′; reverse: 5′-GGATGGCAAATACACGGATAAAG-3′), GAPDH (forward: 5′-AGGTCGGTGTGAACGGATTTG-3′; reverse: 5′-TGTAGACCATGTAGTTGAGGTCA-3′) and 18S rRNA (forward: 5′-GTAACCCGTTGAACCCCATT-3′; reverse: 5′-CCATCCAATCGGTAGTAGCG-3′) genes. The reactions were incubated at 95 °C for 5 min, followed by 40 cycles of 95 °C for 30 s, 60 °C for 30 s and 72 °C for 1 min. After the reactions were completed, the C_T_ values were determined, and the relative level of mRNA was normalized to GAPDH or 18S rRNA and was calculated by the 2^−ΔΔCT^ method.

### Calculation of the absolute levels of siRNAs in tissues

A literature screen revealed that mice are estimated to contain 157.3 million, 515.6 million and 350.6 million cells per g of liver, lung and kidney, respectively.^56^ In the quantitative RT-PCR assays, the absolute amounts of EGFR siRNA in 1 μg of total RNA isolated from each mouse tissue sample were measured. EGFR siRNA was calculated to be present at 0.272 fmol, 0.083 fmol and 0.013 fmol per μg of total RNA in the liver, lung and kidney cells, respectively. Normally, 2000, 1000 and 1500 μg of total RNA can be isolated from 1 g of mouse liver, lung and kidney tissue, respectively. Accordingly, the final concentrations (copies per cell) of EGFR siRNA in these tissues were calculated.

### In situ hybridizations

In situ hybridizations were performed in 15 μm cryosections from formaldehyde-fixed mouse tissues. Sections were then pre-hybridized in hybridization solution (50% formamide, 5× SSC, 0.5 mg/mL yeast tRNA, 1× Denhardt’s solution) at 25 °C below the predicted Tm value of the LNA probe for 30 min. Probes (3 pmol) (miRCURY LNA miRNA detection probe; Qiagen) were DIG-labelled and hybridized to the sections for 10 h at the same temperature as pre-hybridization. The slides were washed in 0.1× SSC three times at 60 °C, once for 5 min in 2× SSC at room temperature with agitation. After blocking, anti-Digoxigenin (Anti-Digoxigenin (mouse-IgG); Roche, IN, USA) was added to the sections slides. The slides were then washed with TNT buffer and incubated with fluorescence secondary antibody (Donkey anti-Mouse IgG (H + L) ReadyProbes Secondary Antibody, Alexa Fluor 594; Invitrogen). After washing again, slides were mounted in Prolong Gold containing DAPI (ProLong Gold Antifade Mountant with DAPI; Invitrogen, P36935, MA, USA).

### Western blotting

Cells were rinsed with PBS (pH 7.4) and then lysed in RIPA lysis buffer (Beyotime, Shanghai, China) supplemented with a protease and phosphatase inhibitor cocktail (Thermo Scientific, Rockford, IL) on ice for 30 min. The tissue samples were frozen solid in liquid nitrogen, ground into a powder and lysed in RIPA lysis buffer containing a protease and phosphatase inhibitor cocktail on ice for 30 min. When necessary, the samples were sonicated in an ice bath. Cell lysates and tissue homogenates were centrifuged for 10 min (12,000× *g* at 4 °C). The supernatant was collected, and the protein concentration was determined using a Pierce BCA Protein Assay kit (Thermo Scientific, 23225, CA, USA). Subsequently, protein lysates (20 μg) were loaded onto acrylamide gels for electrophoretic separation of proteins under denaturing conditions and transferred onto PVDF membranes. Total protein was stained with Ponceau (Solarbio, P0012, Beijing, China) to ensure equal loading. The membranes were then blocked for 1 h at room temperature with 5% nonfat dry milk in TBS with 0.1% Tween-20 (for phosphorylated protein, the membranes were blocked for 1 h with 5% BSA in TBS with 0.1% Tween-20) and incubated overnight at 4 °C with the primary antibodies. The membrane was washed 3 times with 1× TBST for 20 min each. Secondary antibodies were incubated for 1 h at room temperature followed by another 3 times of 10-min wash with 1× TBST. The membrane was incubated with ECL substrate (Thermo Fisher, 46640, CA, USA) according to the manufacturer’s instructions and detected with auto radiography film in a darkroom or read using a chemiluminescence imaging system. The protein bands were analyzed using ImageJ.

Antibodies used were listed as below: anti-EGFR (A2B1) (18986-1-AP), anti-KRAS (12063-1-AP) and anti-p-AKT (Ser473) (66444-1-Ig) antibodies were purchased from Proteintech (IL, USA), and the anti-KRAS antibody (12063-1-AP, Proteintech) recognized total KRAS (wild-type KRAS and mutant KRAS) rather than KRAS^G12D^; anti-GAPDH (G-9) (sc-365062) antibody was purchased from Santa Cruz Biotechnology (Santa Cruz, CA, USA); anti-TNC (ab108930) and anti-IR (ab131238) antibodies were purchased from Abcam (Cambridge, MA, USA); anti-Flag antibody (MA1-91878) was purchased from Thermo Fisher (San Jose, CA, USA); anti-PTP1B (A1590) antibody was purchased from ABclonal (Cambridge, MA, USA); anti-p-Erk1/2 (Thr202/Tyr204) (4376) antibody was purchased from Cell Signalling Technology (Danvers, MA, USA); and anti-p-IR/IGF1R (Tyr1158/Tyr1162/Tyr1163) (P06213) antibody was purchased from Millipore (Bedford, MA, USA).

### Histopathology and immunohistochemistry

Histopathological examination was performed as previously reported.^57^ Briefly, tissues were fixed in 4% paraformaldehyde overnight and embedded in paraffin. Then sections obtained using a microtome were collected on glass slides, stained with haematoxylin and eosin and mounted. Tumor burden was confirmed by a pathologist in a blinded fashion.

Immunohistochemistry was performed according to standard protocols. Prior to staining, sections were baked at 60 °C for 1 h, de-paraffinized in xylene and rehydrated through graded ethanol. Antigen retrieval was performed by heating the sections under high pressure in citrate antigen retrieval solution for about 5 min. Sections were incubated with primary antibodies against EGFR, PCNA, KRAS, p-AKT, p-ERK or TNC for 60 min at room temperature. The same primary antibodies were used as in western blot assay. The immunoreaction was detected by treatment with diaminobenzidine chromogen for 3 min. TUNEL assay was conducted using the Apoptosis Detection Kit (Servicebio, GDP1041, Wuhan, China). Protein expression was assessed by an experienced pathologist blinded to experimental conditions and calculated with Image Pro Plus software.

### Exosome isolation and analyses

#### Exosomes isolated from cell culture medium

HEK293T cells transfected with or without the genetic circuits (plasmids) were cultured in serum-free Opti-MEM for 36 h. The cell culture medium was then harvested from HEK293T cells and centrifuged at 2000× *g* for 30 min. The supernatant containing the cell-free culture medium was transferred to a new tube without disturbing the pellet. Subsequently, total exosomes were isolated by Ribo Exosome Isolation Reagent (from cell culture media) (Ribobio, C10130, Guangzhou, China) according to the manufacturer’s instruction. The exosome pellet was resuspended in PBS or RIPA lysis buffer. The exosomes resuspended in PBS were subjected to cell incubation assay, transmission electron microscopy, nanoparticle tracking analysis. The exosomes resuspended in RIPA lysis buffer were subjected to western blotting for specific exosome markers CD63 (Proteintech, 25682-1-AP, IL, USA), TSG101 (Proteintech, 28283-1-AP, IL, USA) and CD9 (Proteintech, 20597-1-AP, IL, USA). Approximately 5.7 × 10^9^ exosomes (20 μg total protein content) could be harvested from 1 × 10^7^ HEK293T cells.

#### Exosomes isolated from plasma

After tail vein injection with genetic circuits (plasmids), plasma was collected from each mouse and centrifuged at 2000× *g* for 20 min and then at 10,000× *g* for 20 min to remove cells and debris. Approximately 200 μL of plasma was obtained from each mouse and was equally divided into two samples, one (~100 μL) for direct isolation of total RNA and quantification of total siRNA levels, and the other for purification of exosomes. Plasma exosomes were isolated using the Total Exosome Isolation Kit (from plasma) (Invitrogen, 4484450, MA, USA) according to manufacturer’s instructions. In detail, equal volume (~100 μL) of PBS was added to the plasma followed by 0.2 total volume (~40 μL) of the Exosome Precipitation Reagent. After incubation of the sample at room temperature for 10 min, exosomes and supernatant can be separated by centrifugation at 10,000× *g* for 5 min at room temperature. The exosome pellet was resuspended in PBS, RIPA lysis buffer or TRIzol reagent. The exosomes resuspended in PBS were subjected to transmission electron microscopy or nanoparticle tracking analysis. The exosomes resuspended in RIPA lysis buffer were subjected to western blotting for specific exosome markers CD63, TSG101 and CD9. Plasma-enriched albumin (Proteintech, 16475-1-AP, IL, USA) was assessed simultaneously as a negative control. The exosomes resuspended in TRIzol reagent were subjected to isolation of total RNA and quantification of exosomal siRNA levels. For exosome-free supernatant (~220 μL), 800 μL TRIzol reagent was added to extract total RNA and quantification of exosome-free siRNA levels. Because plasma exosome pellets and exosome-free plasma are from the same plasma sample, the levels of total siRNA, exosomal siRNA and exosome-free siRNA are comparable in the same plasma sample. Approximately 1.1 × 10^12^ exosomes (625 μg total protein content) could be harvested from 1 mL plasma.

#### Exosomes incubation assay

HEK293T cells were seeded into 10-cm dishes and transfected with 10 μg genetic circuits (plasmids) using Lipofectamine 2000 reagent (Invitrogen). The cell culture medium was then harvested after 36 h and exosomes were isolated as mentioned above. Approximately 5.7 × 10^9^ exosomes (20 μg total protein content) were incubated with 1 × 10^6^ LLC or U87MG cells. Cells were incubated with exosomes for 36 h, after which total RNA or protein were isolated for further analysis.

#### Electron microscopy

Exosomes suspended in PBS were fixed in 2% paraformaldehyde. The fixed sample was absorbed by a copper mesh coated with formaldehyde in a dry environment for 20 min. The sample was fixed in 1% glutaraldehyde for 5 min. After rinsing in distilled water, the sample was dyed with uranyl oxalate for 5 min and then dyed with uranyl acetate for 10 min on ice. Excess liquid is removed from the mesh with a filter paper and the mesh is stored at room temperature until it is imaged. Imaging was performed using a KYKY-EM3200 microscope.

#### Nanosight nanoparticle tracking analysis

Isolated exosomes were analyzed with Nanosight LM10, a system equipped with a blue laser (405 nm). The nanoparticle is illuminated by a laser, and its motion in Brownian motion is captured for 60 s. At least 5 videos were collected from each individual sample to provide representative concentration measurements, and all control samples were run alongside experimental samples. The size distribution curves were evaluated with NTA software and were averaged within each sample in the video repetition, and then averaged between repetitions to provide a representative size distribution.

### Immunoprecipitation assay

An immunoprecipitation assay was performed to detect the exosomes bearing the RVG tag on the exosomal surface in vivo. Briefly, instead of the RVG peptide, a Flag epitope was cloned into Lamp2b. Circuits encoding control scrambled RNA (CMV-scrR), EGFR siRNA (CMV-siR^E^) or EGFR and TNC siRNAs plus Flag-Lamp2b (CMV-Flag-siR^E+T^) were intravenously injected into mice. After 9 h, exosomes were isolated from mouse plasma and used as input samples for Flag and CD63 western blots. CD63 was used as a marker for the presence of exosomes. The codetection of Flag and CD63 bands indicated that Flag was successfully incorporated into the exosomes. To determine whether the Flag epitope was correctly located on the exosomal surface, intact exosomes were immunoprecipitated using beads coated with anti-Flag or anti-IgG antibodies. Western blotting analysis was then performed using anti-Flag antibodies. The enrichment of Flag bands in the anti-Flag-immunoprecipitated products indicated that the exosomes bore the Flag tag on their surface.

### Evaluation of in vivo accumulation of original circuits by CFU approach and quantitative RT-PCR assay

Intact plasmids containing the CMV-siR^E^ circuit and spectinomycin resistance gene (smR) were intravenously injected into C57BL/6J mice at the dose of 5 mg/kg. At 3, 6, 9, 12, 24, 72, 168 and 720 h post-injection, mice were sacrificed, and plasma and tissues (liver, lung, brain, kidney and spleen) were collected. Then, plasmids were extracted from 200 μL plasma or 1 cm^3^ tissues using AxyPrep Plasmid Miniprep Kit (Axygen, AP-MN-P-50, NY, USA) according to the manufacturer’s instructions. Next, purified plasmids were transformed into *E. coli* DH5α competent cells (Tsingke, TSC01, Beijing, China). Briefly, all of the plasmid-eluting solution was added into 100 μL competent *E. coli* and mixed gently and allowed to stabilize for 30 min on ice. The mixed solution was incubated at 42 °C for 90 s and then immediately transferred onto ice for 5 min. Finally, 1 mL LB broth was added into the transformation mixture and allowed to transform for 1 h at 37 °C with shaking. Next, the tube was centrifuged at 1000 rpm for 5 min, and approximately 100 μL resuspended supernatant was plated onto agar-solidified LB broth plates supplemented with spectinomycin. The plates were placed upside down at 37 °C. After 24 h, the bacterial colonies were photographed and counted. Because only the cells that have integrated intact plasmids can survive under spectinomycin selection, the number of colonies formed in plates could represent the relative abundance of original genetic circuits present in plasma and tissues.

In quantitative RT-PCR assay, the dynamic range and sensitivity for measuring the CMV-siR^E^ circuit was first determined. Synthetic CMV-siR^E^ circuits (in the form of DNA plasmid) were serially diluted over several orders of magnitude, corresponding to levels ranging from 10^−1^ μg to 10^−6^ μg and were assessed via quantitative RT-PCR. The resulting C_T_ values were plotted against the amount of input CMV-siR^E^ circuits to generate a standard curve. Then the C_T_ values of the CMV-siR^E^ circuit in the liver (~10 mg) were measured. By referring to the standard curve, the absolute amounts of CMV-siR^E^ circuit at different time points were calculated. Previous study revealed that mice were estimated to contain 157.3 million cells per g of liver.^56^ Accordingly, the final concentration (copies per cell) of CMV-siR^E^ circuit in the liver was calculated.

### Injection of genetic circuits via the tail vein or common bile duct

Six- to eight-week-old male C57BL/6J mice were purchased from the Model Animal Research Centre of Nanjing University (Nanjing, China) and maintained under specific pathogen-free conditions at Nanjing University. The mice were injected with genetic circuits (plasmids) via the tail vein or common bile duct (0.05, 0.5 or 5 mg/kg). At various time points (0, 1, 3, 6, 9, 12, 24, 36 and 48 h) post-injection, 3 mice were sacrificed, blood samples were collected via cardiac puncture, and plasma and CD4^+^ T cells were purified from the blood, and liver, spleen, heart, lung, kidney, pancreas, brain, skeletal muscle and colon tissues were collected. All animal care and handling procedures were performed in accordance with the National Institutes of Health Guidelines for the Care and Use of Laboratory Animals and were approved by the Institutional Review Board of Nanjing University (Nanjing, China).

For common bile duct injection, mice were anesthetized with pentobarbital (70 mg/kg, i.p.). Mice were then placed under a stereomicroscope while in a supine position, and the abdomen was cleaned using 70% ethanol. A laparotomy was performed, and the skin and muscle tissue of the thorax were cut using a V-incision from the pubic region up to the diaphragm to expose the abdominal cavity. The skin was effectively separated from the exposed organs to avoid contamination by the mouse fur. The lobes of the liver were then positioned against the diaphragm to expose the gall bladder and the proximal segment of the common bile duct. Then, the duodenum was gripped along the common bile duct using forceps and clamped at the level of the ampulla of Vater. The ampulla of Vater is a triangle-shaped white area located at the confluence between the common bile duct and the duodenum. Once the common bile duct was clamped, it was cannulated. The standard method for this procedure consists of inserting a needle into the middle of the common bile duct and orienting it towards the hepatic duct. The circuit (plasmid) was then injected into the common bile duct, which extends directly into the liver. Finally, the abdomen was stitched using continuous sutures.

### eGFP fluorescence intensity detection

Tissues were rinsed with PBS (pH 7.4) and then cut into pieces and lysed in RIPA lysis buffer using freshly added PMSF for 30 min on ice; a tissue homogenizer was used to facilitate tissue disruption. After the tissues were centrifuged at 12,000× *g* at 4 °C for 10 min, the supernatants were collected, and protein concentrations were quantified using a BCA protein assay kit. The supernatants were diluted 10 times with double-distilled water, and a FluoroMax-2 spectrofluorometer was used to detect the 509 nm fluorescence value of the samples excited at 395 nm. The results were normalized to the total protein concentrations.

### Evaluation of the therapeutic effects of the genetic circuits targeting EGFR- and KRAS-driven lung cancer

#### Orthotopic lung cancer model

To generate an orthotopic lung cancer model, we intravenously injected 5 × 10^6^ LLC cells into nude mice via the tail vein.^58^ After 30 days, the mice were monitored using noninvasive micro-CT scanning to ensure successful tumor formation in the lungs. Then, the tumor-bearing mice were randomly divided into four groups: three were intravenously injected with PBS or 5 mg/kg CMV-scrR or CMV-siR^E^ circuit, and one was intragastrically administered 5 mg/kg gefitinib for a total of 7 times over the course of 2 weeks. Gefitinib (MCE, ZD1839, NJ, USA) was selected as a negative control because it did not significantly inhibit the growth of LLC cells.^30^ After the circuits were injected and gefitinib was administered, the mice were divided and monitored to evaluate either survival time or tumor growth. For the survival analysis, mice were monitored for survival post circuit injection without any further treatment. For tumor growth analysis, only mice that survived the 2-week circuit injection period were analyzed using micro-CT. After micro-CT scanning, the mice were sacrificed, blood samples were collected via cardiac puncture, and lung tumors were isolated and analyzed by histological analysis (H&E staining). Excised lung tumors were also processed to determine EGFR expression by western blotting and immunohistochemical analysis. Tumor cell proliferation rate was determined by immunohistochemical analysis of PCNA and manifested by the percentage of PCNA-positive tumor cells. Quantification of immunohistochemical staining was performed by Image pro plus.

#### Spontaneous lung cancer model

The *KRAS*^*LSL-G12D*^*;p53*^*flfl*^ mice were housed in a specific pathogen-free environment and treated in strict accordance with protocols approved by the Institutional Review Board of Nanjing University. For *KRAS*^*G12D*^ activation in mouse lungs,^59^ six-week-old *KRAS*^*LSL-G12D*^*;p53*^*flfl*^ mice were first anesthetized with pentobarbital (70 mg/kg, i.p.), and then 5 × 10^6^ plaque-forming units of Adeno-Cre (Obio, Shanghai, China) were diluted with PBS to obtain a final volume of 50 μL and given through intratracheal administration.^60^ Mice were euthanized at different times (30, 40 and 50 days) post-inhalation to ensure tumor formation and progression, and the tumors were found to be approximately evenly distributed in the lung tissue. After 50 days of Adeno-Cre administration, mice were randomly divided into two groups and treated for 2 weeks (7 injections) through the tail vein with 5 mg/kg CMV-scrR or CMV-siR^K^ circuit. Then, the mice were monitored to determine survival time or assess tumor growth. For the survival analysis, mice were monitored for survival post circuit injection without any further treatment. For tumor growth analysis, mice were analyzed using micro-CT after the 2-week circuit injection period. After micro-CT scanning, the mice were sacrificed, blood samples were collected via cardiac puncture, and lung tissues were isolated and analyzed by H&E staining to confirm lung adenocarcinoma formation. Excised lung adenocarcinomas were also processed to determine the expression of KRAS, p-ERK and p-AKT by western blotting and immunohistochemical analysis. Tumor cell proliferation rate was determined by immunohistochemical analysis of PCNA and manifested by the percentage of PCNA-positive tumor cells. Tumor cell apoptosis rate was determined by TUNEL assay and manifested by the percentage of TUNEL-positive cells. Quantification of immunohistochemical staining was performed by Image pro plus.

#### Micro-CT scanning

Micro-CT analysis was performed to assess lung tumor growth because the micro-CT images clearly distinguished the lung tumors from the surrounding tissue even without any contrast agent, and the reconstructed 3-D pulmonary images easily differentiated the tumors from blood vessels.^61,62^ Briefly, micro-CT scans were performed using a SkyScan 1176 micro-CT analyzer, which scanned a 180° area at a resolution of 35 μM with a rotation step of 0.800. The system comprised two metallo-ceramic tubes equipped with a fixed 0.5 mm Al filter and two 1280 × 1024 pixel digital X-ray cameras. Images were acquired at 50 kV and 500 μA. The mice were scanned while in a supine position. The micro-CT data were batch-sorted, processed and reconstructed using the N-Recon programme, according to the manufacturer’s instructions (SkyScan). The reconstructed data were subsequently imaged using DataViewer, and the tumor volumes were calculated using the CTan programme according to the manufacturer’s instructions (SkyScan).

### Evaluation of the therapeutic effects of the brain-directed genetic circuits targeting glioblastoma

#### Intracranial glioblastoma model

The lentiviral vector pLv-Luc encoding luciferase and puromycin was purchased from GenePharma (Shanghai, China). The pLv-Luc lentivirus was transduced into a parent U87MG cell line to generate a bioluminescent U87MG-luciferase-puromycin (U87MG-Luc)-expressing cell line. Luc-expressing cells were selected using 1 μg/mL G418 (Gibco BRL). To generate the glioblastoma mouse model, we intracranially implanted 1 × 10^6^ U87MG-Luc cells into nude mice at the following coordinates with respect to the bregma: 0.5 mm posterior, 2.5 mm lateral and 3.5 mm intraparenchymal.^63^ The tumor cells were allowed to engraft for 7 days, and mice were monitored using noninvasive BLI to ensure successful glioblastoma formation in the brain. Then, the glioblastoma-bearing mice were randomly divided into 4 groups and intravenously injected with PBS or 5 mg/kg CMV-scrR, CMV-RVG-siR^E^ or CMV-RVG-siR^E+T^ circuit for a total of 7 times over 2 weeks. The mice were either followed-up for survival analysis or scanned using BLI on day 7, 14, 28 and 35 post-implantation to evaluate tumor growth. For each mouse, the data were normalized to the level of bioluminescence observed at the initiation of the treatment. After BLI scanning, mice were sacrificed for the collection of blood and tissue samples. Excised glioblastomas were processed to determine the expression of EGFR and TNC by western blotting and immunohistochemical analysis. Tumor cell proliferation rate was determined by immunohistochemical analysis of PCNA. Quantification of immunohistochemical staining was performed by Image pro plus.

#### Bioluminescence imaging

Mice were anesthetized via intraperitoneal pentobarbital injection (70 mg/kg). The bioluminescent signals emitted from the engrafted tumors were recorded using a Perkin IVIS system (PerkinElmer, MA, USA). For quantification of the bioluminescence, identical regions of interest were drawn. The radiance was determined using IVIS software Living Image version 4.2 (Perkin Elmer).

#### Tracking the delivery of fluorescently-labelled exosomes into glioblastoma

To evaluate the targeting of brain-directed exosomes to the U87MG cells in the mouse model of glioblastoma, CMV-scrR or CMV-RVG-siR^E+T^ circuit was intravenously injected into C57BL/6J mice, and the exosomes fractions were isolated from the plasma. The purified exosomes were stained with PKH26 (Sigma Aldrich, PKH26GL, Darmstadt, Germany), a red-fluorescent lipophilic dye that can label lipid bilayers of exosomes, and then the fluorescently-labelled exosomes were injected into glioblastoma-bearing nude mice. Free PKH26 dye was injected as a control. After treatment, mice were sacrificed, and the glioblastoma and peripheral tissues were dissected and frozen sectioned. A Leica TCS SP8 MP confocal microscopy was applied to detect the red fluorescent signal in the frozen sections.

### Evaluation of the therapeutic effects of the brain-directed genetic circuits targeting obesity

#### Obesity model

Male C57BL/6J mice (3 weeks of age) and *ob/ob* mice were obtained from the Model Animal Research Center of Nanjing University. For HFD model, C57BL/6J mice were maintained on a HFD (Research Diets cat. # D12492, New Brunswick, NJ, USA) with a 12-h light cycle for 12 weeks.^64^ D12492 consists of 60% fat, 20% carbohydrate and 20% protein with a total of 5.24 kcal/g. Food intake and body weight were measured twice a week. HFD-induced obese mice and *ob/ob* mice were intravenously injected with PBS or 5 mg/kg CMV-scrR, CMV-siR^P^ or CMV-RVG-siR^P^ circuit every 2 days for a total of 12 times to evaluate the antiobesity effects.

#### Immunofluorescence staining

Mice were fasted overnight (16 h) and injected intraperitoneally with mouse recombinant leptin (5 μg/g, PeproTech, 450-31, NJ, USA).^38^ Forty-five minutes after injection, the animals were deeply anesthetized with sodium pentobarbital (50 mg/kg, intraperitoneal) and perfused via transcardial perfusion with 1× PBS followed by ice-cold 4% PFA. Brains were postfixed for 4 h in 4% PFA and cryoprotected in 20% and 30% sucrose in 1× PBS at 4 °C. For immunofluorescence analysis, sections were postfixed for 10 min in 4% PFA and then washed with 1× PBS prior to blocking with 5% normal horse serum/0.25% Triton X-100 in PBS (1 h). Sections were then incubated with PTP1B (ABclonal, A1590, Cambridge, MA, USA) primary antibodies diluted 1:100 in blocking solution overnight. The following day, sections were washed with 1× PBS and subsequently incubated in blocking solution containing secondary antibody for 1 h. Then, the sections were washed with 1× PBS and placed in DAPI staining solution for 10 min. After washing with 1× PBS, the sections were ready for examination.

#### Energy expenditure measurements

Mice were acclimated to an open-circuit indirect calorimetry cage (TSE PhenoMaster System) for 24 h, and energy expenditure was measured at 18-min intervals for 72 h. Oxygen consumption (VO_2_) and carbon dioxide production (VCO_2_) were measured using electrochemical and spectrophotometric sensors.^40^ The respiratory exchange ratio (RER) was calculated as the ratio of VCO_2_ to VO_2_. Locomotor activity was measured simultaneously by infrared light beam sensor frames. Heat production in individual mice was calculated using the Weir equation.

#### Leptin sensitivity

For leptin sensitivity measurements in vivo, recombinant murine leptin (PeproTech, 450-31, NJ, USA) was administered intraperitoneally to HFD male mice.^65^ Mice were fasted overnight prior to injection. Leptin was given intraperitoneally twice a day (morning and evening; 0.5 μg/g/injection) for the indicated period of time (36 h). Body weight and food intake were monitored daily for 5 days. Body weight and baseline food intake measurements for the 2 days prior to the start of the experiment were averaged and used to calculate the change percentage.

#### Glucose homoeostasis

Blood glucose was measured in tail blood using a glucometer (Abbott Laboratories, USA). ITTs were performed on fasted (6 h) mice. Blood glucose values were measured immediately before and at 15, 30, 45, 60, 90 and 120 min after intraperitoneal injection of human crystalline insulin (1 U/kg of body weight).^42^ GTTs were performed on fasted (12 h) mice. Animals were injected intraperitoneally with d-glucose (20% solution; 1 g/kg of body weight), and blood glucose values were determined at 0, 15, 30, 60 and 90 min post-injection.

#### Other metabolic measurements

The serum insulin concentration was determined by enzyme-linked immunosorbent assay using mouse insulin as a standard (Millipore, EZRMI-13K, MA, USA). Serum leptin was also determined by enzyme-linked immunosorbent assay (MultiSciences, 70-EK297, Shanghai, China).^66^ Triglyceride, total cholesterol, high-density lipoprotein cholesterol and low-density lipoprotein cholesterol values were measured by an enzymatic colorimetric method (Jiancheng, Nanjing, China).

### Analyses of serum biochemical indicators, tissue damage and immunotoxicity

C57BL/6J mice were intravenously injected with PBS or 5 mg/kg CMV-scrR or CMV-siR^E^ circuit every 2 days for a total of 7 times. Twelve hours after the last injection, the mice were euthanized to collect peripheral blood and tissues. The weights of lymphoid organs (thymus and spleen) were measured. The specimen of liver, lung, spleen and kidney were fixed in 4% PFA for H&E staining to evaluate tissue damage. Fresh peripheral blood was divided into two samples. One was subjected to separation of serum and measurement of the representative serum biochemical indicators, including alanine aminotransferase (ALT), aspartate aminotransferase (AST), total bilirubin (TBIL), blood urea nitrogen (BUN), alkaline phosphatase (ALP) and creatinine (CREA). The other was treated with Red Blood Cell Lysis Buffer (Solarbio, R1010, Beijing, China) for lymphocyte analysis. The lymphocyte subsets were then stained for 15 min with the following antibodies for each subset: anti-CD45 and anti-CD3 for T cells; anti-CD45 and anti-NK-1.1 for NK cells; anti-CD45 and anti-CD19 for B cells; anti-CD45 and anti-F4/80 for monocytes; anti-CD45 and anti-CD11c for dendritic cells; anti-CD45 and anti-Ly-6G/Ly-6C for neutrophils. All the primary antibodies were purchased from Biolegend (CA, USA): APC/Cyanine7 anti-mouse CD45 antibody (AB_312980), PerCP/Cyanine5.5 anti-mouse CD3 antibody (100217), PE/Cyanine7 anti-mouse NK-1.1 antibody (108713), FITC anti-mouse CD19 antibody (101505), APC anti-mouse F4/80 antibody (123115), FITC anti-mouse CD11c antibody (117305) and PE/Cyanine7 anti-mouse Ly-6G/Ly-6C (Gr-1) antibody (108415). The cells were washed twice and resuspended with D-hanks Buffer (Solarbio, H1045, Beijing, China) for flow cytometry analysis. The counts of peripheral immune cells (T cell, B cell, NK cell, DC, monocyte and neutrophil) were analyzed with Attune NxT (Thermofisher, CA, USA) and the results was analyzed with Flowjo 10 software.

### Statistical analysis

Details on statistics used can be found in figure legends. All statistical analyses were performed using commercially available software (GraphPad Prism 7). Kaplan–Meier was used for survival representations. Data were first checked for normal distribution, differences among groups were compared by one-way ANOVA, and multiple comparisons were conducted by the Dunnett’s test. *n* represents the number of samples used in the experiments. Data are shown with means with error bars showing the SEM. Significance was assumed with *P < 0.05; **P < 0.01; ***P < 0.005.

## Supplementary information

Fig. S1

Fig. S2

Fig. S3

Fig. S4

Fig. S5

Fig. S6

Fig. S7

Fig. S8

Fig. S9

Fig. S10

Fig. S11

Fig. S12

Fig. S13

Fig. S14

Fig. S15

Fig. S16

Fig. S17

Fig. S18

Fig. S19

Fig. S20

Fig. S21

Fig. S22

Fig. S23

Fig. S24

Fig. S25

Fig. S26

Fig. S27

Fig. S28

Fig. S29

Fig. S30

Fig. S31

Fig. S32

Fig. S33
