# Supplementary material for: In vivo self-assembled small RNAs as a new generation of RNAi therapeutics
Source: Cell Res. 2021 Mar 29;31(6):631–48. doi: 10.1038/s41422-021-00491-z (PMC8169669; doi:10.1038/s41422-021-00491-z)

Fig. S1. Selection of an optimal pre-miRNA backbone to produce siRNA. EGFR siRNA was ligated into constructs carrying an expression cassette for a series of pre-miRNAs. A quantitative RT-PCR assay was performed to assess EGFR siRNA levels in HEK293T cells transfected with these constructs (n = 3 in each group). Values are presented as the means ± SEM.

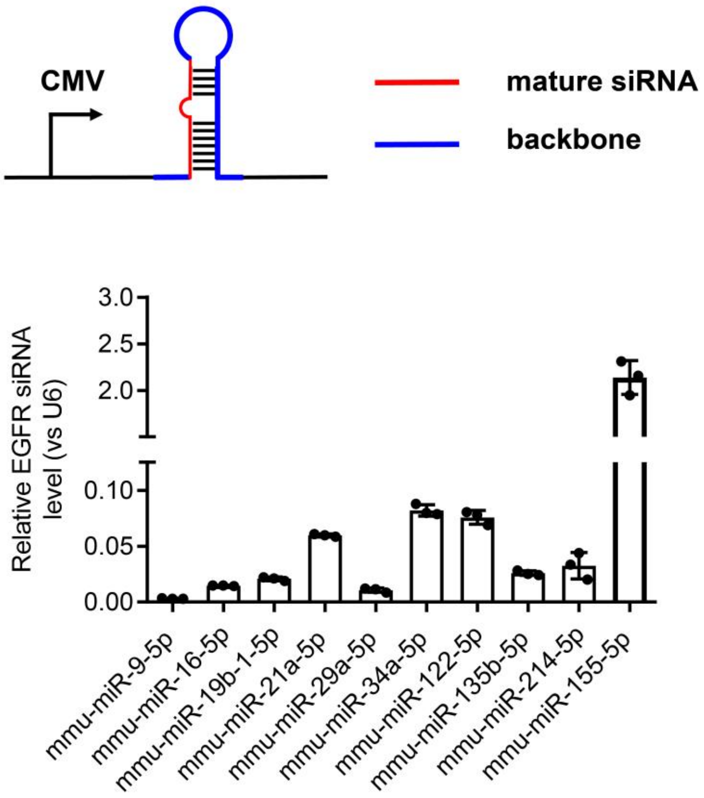

Supplement: Supplementary file 1 — Fig. S1 [file 41422_2021_491_MOESM1_ESM.pdf]
