# Supplementary material for: In vivo self-assembled small RNAs as a new generation of RNAi therapeutics
Source: Cell Res. 2021 Mar 29;31(6):631–48. doi: 10.1038/s41422-021-00491-z (PMC8169669; doi:10.1038/s41422-021-00491-z)

Fig. S2. Characterization of the genetic circuits in Hepa 1-6 cells. (a) Quantitative RT-PCR analysis of the levels of the desired guide strand and undesired passenger strand generated using the pre-miRNA strategy (EGFR siRNA is embedded in pre-miR-155 and is controlled by a CMV promoter, named CMV-siR^{E}) or the shRNA strategy (EGFR siRNA is placed in an shRNA and is controlled by a U6 promoter, named U6-siR^{E}) in Hepa 1-6 cells (n = 3 in each group). **(b)** Quantitative RT-PCR analysis of EGFR siRNA levels in exosomes derived from Hepa 1-6 cells transfected with the CMV-scrR or CMV-siR^{E} circuit (n = 3 in each group). **(c)** A CMV-directed Flag-Lamp2b fusion construct (CMV-Flag-Lamp2b) or a CMV-directed Lamp2b construct (CMV-Lamp2b) was transfected into Hepa 1-6 cells. Exosomes were then isolated and either directly loaded for western blotting with anti-Flag and anti-CD63 antibodies (equal CD63 band densities indicate similar exosome levels) or immunoprecipitated with IgG or anti-Flag beads before western blotting. **(d)** The CMV-siR^{E}, CMV-siR^{T} or CMV-siR^{E+T} circuits were transfected into Hepa 1-6 cells. A quantitative RT-PCR assay was performed to assess the levels of EGFR and TNC siRNAs in transfected Hepa 1-6 cells (n = 3 in each group). **(e)** CMV-scrR, CMV-siR^{E} or CMV-Flag-siR^{E+T} circuits were transfected into Hepa 1-6 cells. Exosomes were then isolated and either directly loaded for western blotting with anti-Flag and anti-CD63 antibodies or immunoprecipitated with IgG or anti-Flag beads before western blotting. A quantitative RT-PCR assay was performed to assess the levels of EGFR and TNC siRNAs in immunoprecipitated exosomes (n = 3 in each group). Significance was determined using two-sided t-test in panel d. NS, not significant.

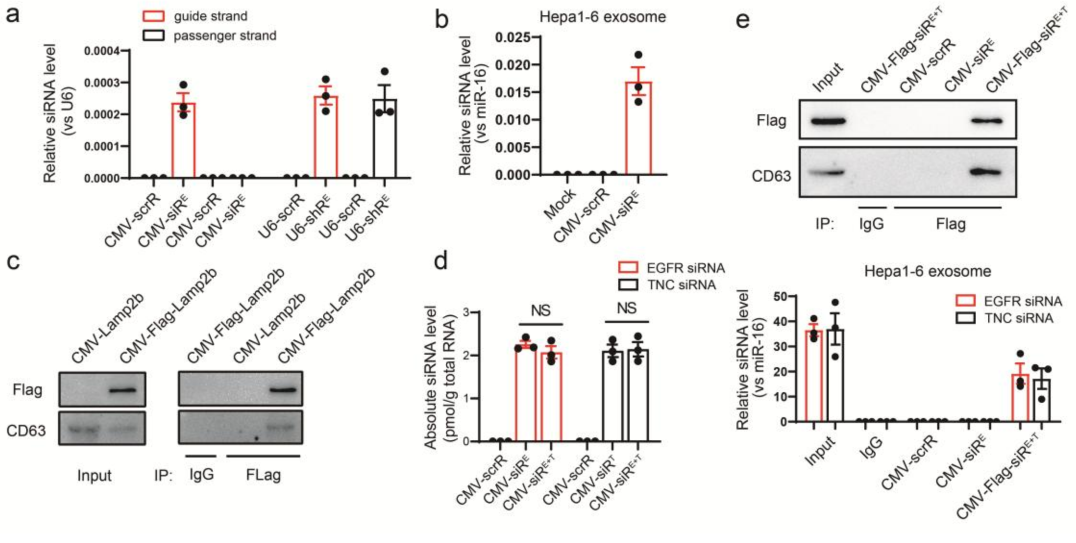

Supplement: Supplementary file 2 — Fig. S2 [file 41422_2021_491_MOESM2_ESM.pdf]
