# Supplementary material for: In vivo self-assembled small RNAs as a new generation of RNAi therapeutics
Source: Cell Res. 2021 Mar 29;31(6):631–48. doi: 10.1038/s41422-021-00491-z (PMC8169669; doi:10.1038/s41422-021-00491-z)

Fig. S4. Selection of an optimal promoter. A CMV- or a U6-driven construct carrying an expression cassette for an EGFR siRNA and an eGFP protein or an RVG-Lamp2b fusion protein was transfected into HEK293T cells. **(a)** The eGFP levels were determined by measuring the eGFP fluorescence signals in microscopy images. Scale bar: 75 μm. **(b)** A quantitative RT-PCR assay was performed to assess the levels of RVG-Lamp2b fusion mRNA in transfected HEK293T cells (n = 6 in each group). Values are presented as the means ± SEM.

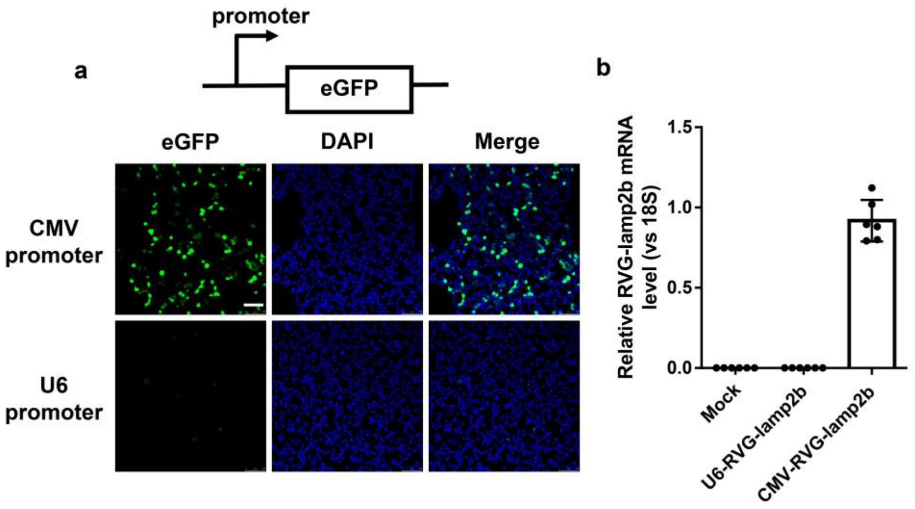

Supplement: Supplementary file 4 — Fig. S4 [file 41422_2021_491_MOESM4_ESM.pdf]
