# Supplementary material for: In vivo self-assembled small RNAs as a new generation of RNAi therapeutics
Source: Cell Res. 2021 Mar 29;31(6):631–48. doi: 10.1038/s41422-021-00491-z (PMC8169669; doi:10.1038/s41422-021-00491-z)

Fig. S5. Association of AGO2 with siRNA in exosomes. CMV-scrR, CMV-siR^{E} or CMV-RVG-siR^{E+T} circuit was transfected into HEK293T cells. Exosomal RNA was then immunoprecipitated using IgG or anti-AGO2 beads before being subjected to western blotting with an anti-AGO2 antibody and quantitative RT-PCR analysis of EGFR siRNA (n = 3 in each group). Values are presented as the means ±SEM.

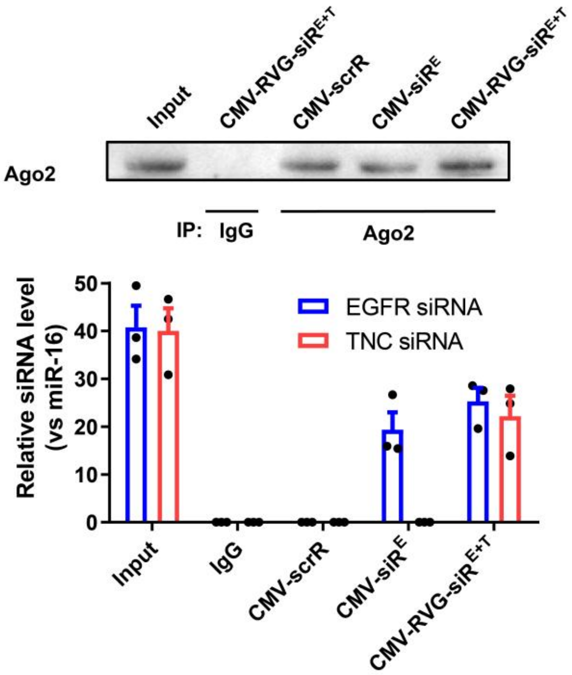

Supplement: Supplementary file 5 — Fig. S5 [file 41422_2021_491_MOESM5_ESM.pdf]
