# Supplementary material for: In vivo self-assembled small RNAs as a new generation of RNAi therapeutics
Source: Cell Res. 2021 Mar 29;31(6):631–48. doi: 10.1038/s41422-021-00491-z (PMC8169669; doi:10.1038/s41422-021-00491-z)

Fig. S6. Western blot analysis of EGFR and TNC protein levels in U87MG cells treated with exosomes with or without the RVG tag on the surface. HEK293T cells were transfected with the CMV-scrR, CMV-siR^{E}, CMV-RVG-siR^{E}, CMV-siR^{T}, CMV-siR^{E+T} or CMV-RVG-siR^{E+T} circuit, and exosomes (100 μg total protein diluted in 100 μl PBS) were purified and incubated with $5×10^{5}$ U87MG cells. Upper panel: representative western blots. Lower panel: quantitative analysis (n = 3 in each group). Values are presented as the means ± SEM. Significance was determined using one-way ANOVA followed by Dunnett's multiple comparison. * $p < 0.05$; ** $p < 0.01$; *** $p < 0.005$; NS, not significant.

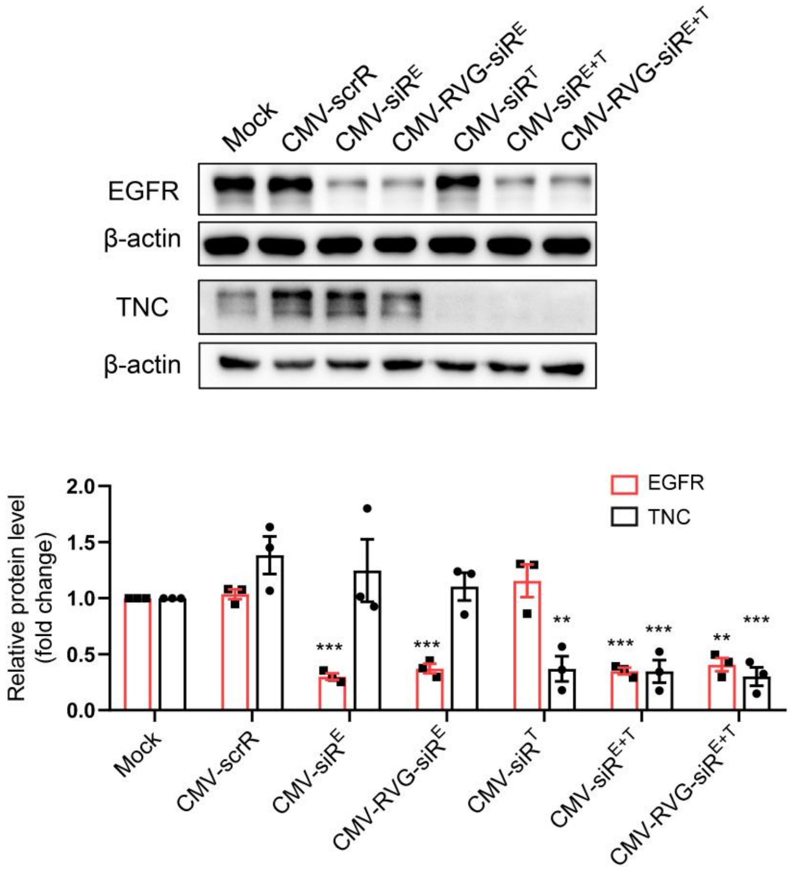

Supplement: Supplementary file 6 — Fig. S6 [file 41422_2021_491_MOESM6_ESM.pdf]
