# Supplementary material for: In vivo self-assembled small RNAs as a new generation of RNAi therapeutics
Source: Cell Res. 2021 Mar 29;31(6):631–48. doi: 10.1038/s41422-021-00491-z (PMC8169669; doi:10.1038/s41422-021-00491-z)
Supplement: Supplementary file 7 — Fig. S7 [file 41422_2021_491_MOESM7_ESM.pdf]

Fig. S7. Evaluation of *in vivo* accumulation of original circuits. (a) Flow chart of the experimental design. Plasmids containing the CMV-siR^E circuit and spectinomycin resistance gene (smR) were intravenously injected into C57BL/6J mice at the dose of 5 mg/kg. Plasma and tissues were collected at various time points post-injection. Plasmids were extracted from plasma and tissues and transformed into *E.coli* DH5α competent cells, and the resultant cells were plated onto agar-solidified LB broth plates supplemented with spectinomycin. Because only the cells that have integrated intact plasmid can survive under spectinomycin selection, the number of colonies formed in plates could represent the relative abundance of original circuit *in vivo*. **(b)** Representative images of colony formation in each plate. **(c)** Statistical analysis of the colonies formed in each plate (n = 3 in each group). **(d)** The dynamic range and sensitivity of the quantitative RT-PCR assay for measuring the CMV-siR^E circuit. Synthetic CMV-siR^E circuits (in the form of DNA plasmid) were serially diluted over several orders of magnitude, corresponding to levels ranging from 10^{-1} μg to 10^{-6} μg and were assessed via quantitative RT-PCR. The resulting C_T values were plotted against the amount of input CMV-siR^E circuits to generate a standard curve. **(e)** The absolute levels of CMV-siR^E circuit in the liver following tail vein injection of 5 mg/kg CMV-siR^E circuit (n = 3 in each group). The C_T values of the CMV-siR^E circuit in the liver (~10 mg) were measured, and then the absolute amounts of CMV-siR^E circuit at different time points were calculated by referring to the standard curve.

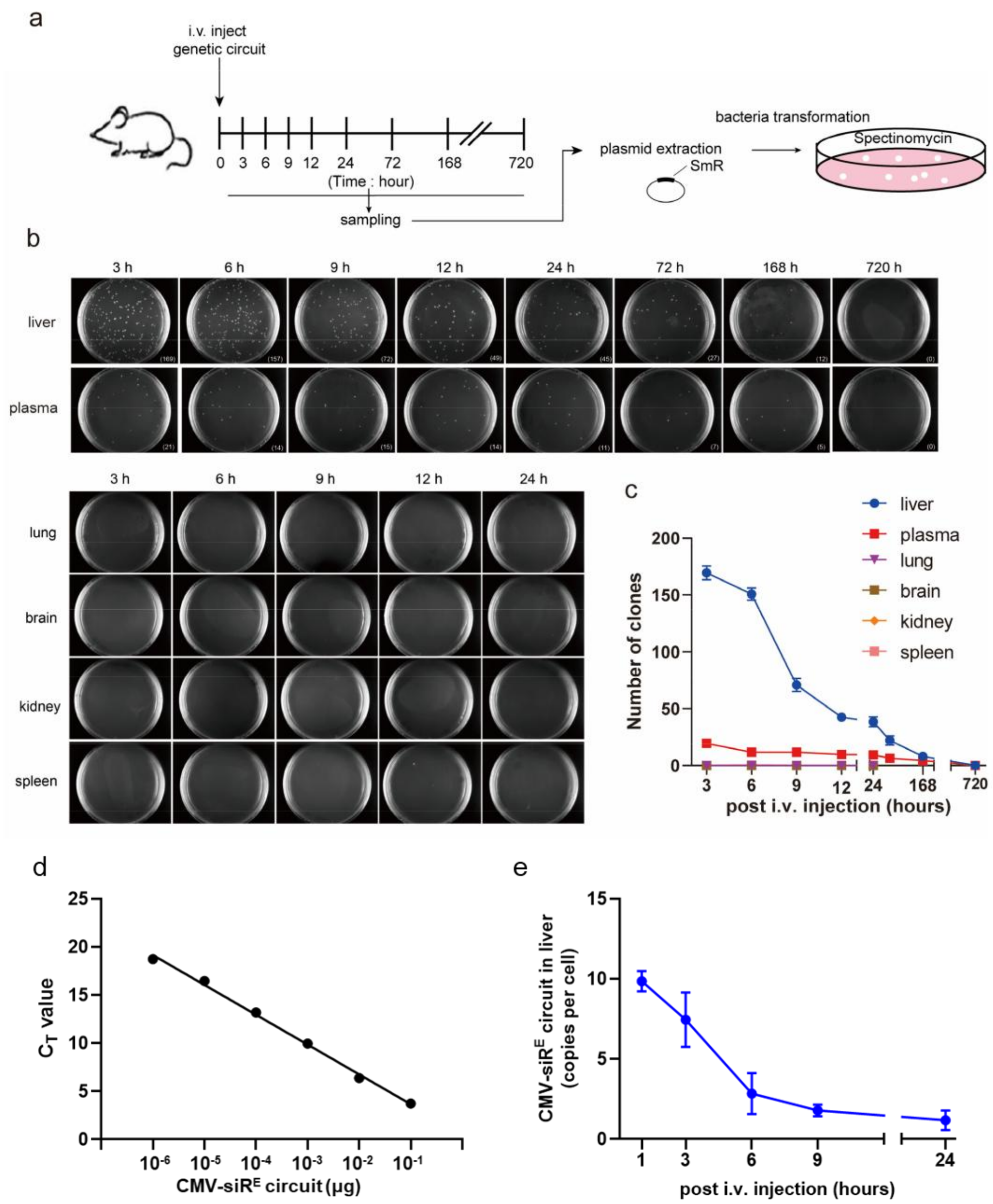
a
i.v. inject
genetic circuit
0 3 6 9 12 24 72 168 720
(Time : hour)
sampling
plasmid extraction
SmR
bacteria transformation
Spectinomycin
b
3 h 6 h 9 h 12 h 24 h 72 h 168 h 720 h
liver
plasma
3 h 6 h 9 h 12 h 24 h
lung
brain
kidney
spleen
c
liver
plasma
lung
brain
kidney
spleen
Number of clones
post i.v. injection (hours)
d
C_T value
CMV-siR^E circuit (μg)
e
CMV-siR^E circuit in liver (copies per cell)
post i.v. injection (hours)
