# Supplementary material for: In vivo self-assembled small RNAs as a new generation of RNAi therapeutics
Source: Cell Res. 2021 Mar 29;31(6):631–48. doi: 10.1038/s41422-021-00491-z (PMC8169669; doi:10.1038/s41422-021-00491-z)

Fig. S8. The 3-D digital image illustrating the kinetics of EGFR siRNA precursor in various mouse tissues following tail vein injection of 5 mg/kg CMV-siR^E circuit.

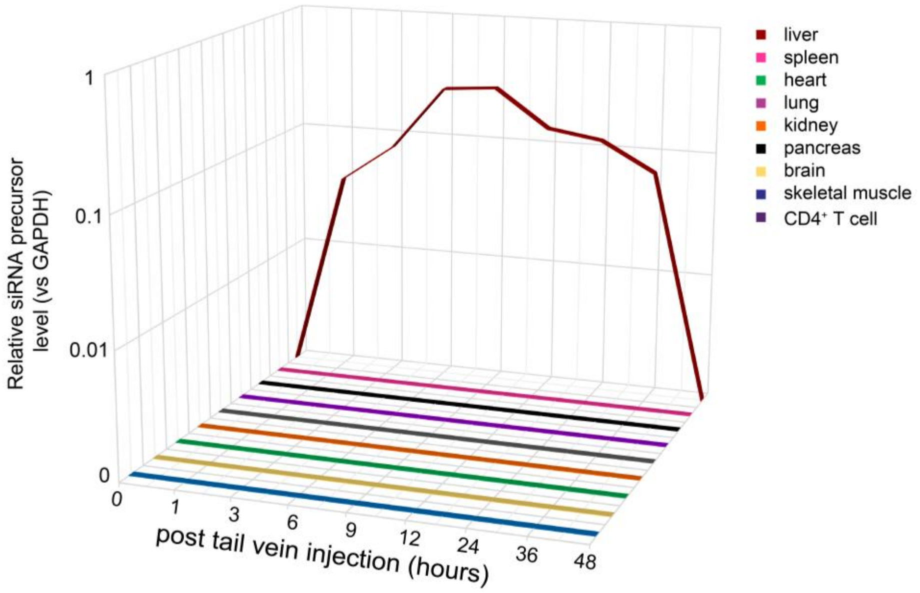

Supplement: Supplementary file 8 — Fig. S8 [file 41422_2021_491_MOESM8_ESM.pdf]
