# Supplementary material for: In vivo self-assembled small RNAs as a new generation of RNAi therapeutics
Source: Cell Res. 2021 Mar 29;31(6):631–48. doi: 10.1038/s41422-021-00491-z (PMC8169669; doi:10.1038/s41422-021-00491-z)

**Fig. S9. Measurement of eGFP levels in various mouse tissues following intravenous injection of a construct coexpressing an EGFR siRNA and an eGFP protein. (a)** Representative fluorescence microscopy images directly showing liver uptake and expression of the injected eGFP construct. Scale bar: 75 μm. **(b)** Representative fluorescence microscopy images of the lung, heart, kidney and spleen at 6 hours post-injection. Positive eGFP signals are shown in green, and DAPI-stained nuclei are shown in blue. Scale bar: 75 μm.

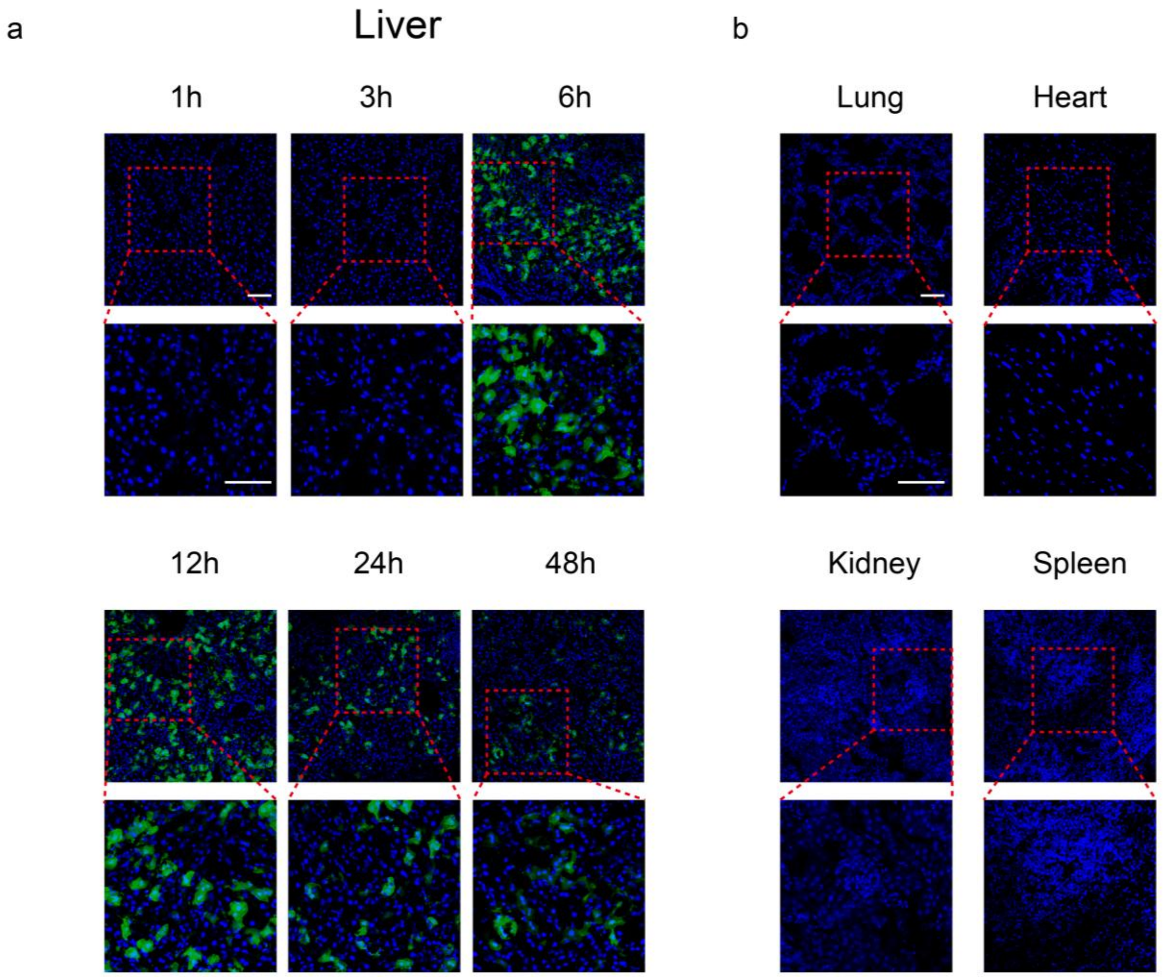

Supplement: Supplementary file 9 — Fig. S9 [file 41422_2021_491_MOESM9_ESM.pdf]
