# Supplementary material for: In vivo self-assembled small RNAs as a new generation of RNAi therapeutics
Source: Cell Res. 2021 Mar 29;31(6):631–48. doi: 10.1038/s41422-021-00491-z (PMC8169669; doi:10.1038/s41422-021-00491-z)

Fig. S10. Characterization of EGFR siRNA in exosomes derived from an *ex vivo* model. Primary mouse hepatocytes were isolated from mice following intravenous injection of 5 mg/kg CMV-scrR or CMV-siR^E circuit. **(a)** A quantitative RT-PCR assay was performed to assess EGFR siRNA levels in exosomes derived from the culture medium of primary hepatocytes (n = 3 in each group). **(b)** The association of EGFR siRNA with AGO2 in hepatocyte exosomes. Exosomal RNA was immunoprecipitated using IgG or anti-AGO2 beads before being subjected to western blotting with an anti-AGO2 antibody and quantitative RT-PCR analysis of EGFR siRNA (n = 3 in each group). Values are presented as the means ± SEM.

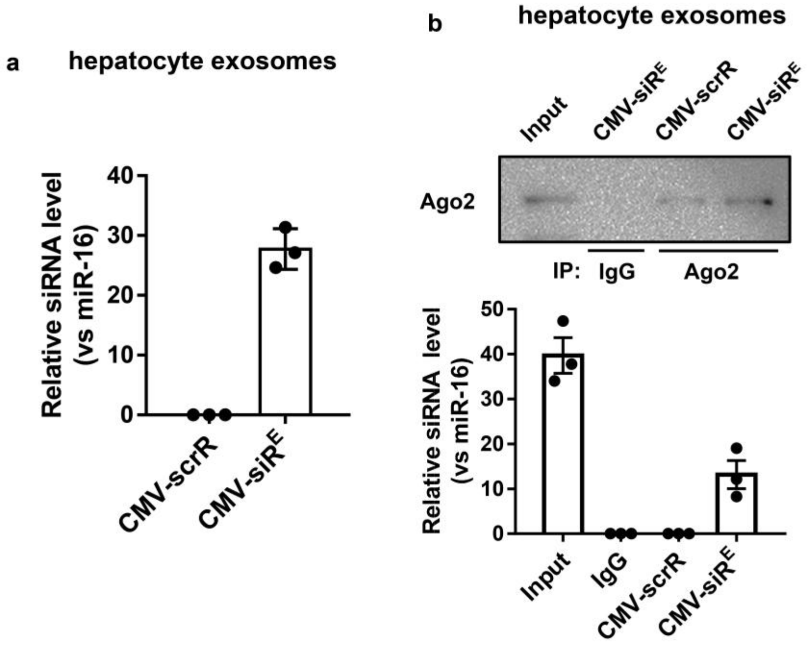

Supplement: Supplementary file 10 — Fig. S10 [file 41422_2021_491_MOESM10_ESM.pdf]
