# Supplementary material for: In vivo self-assembled small RNAs as a new generation of RNAi therapeutics
Source: Cell Res. 2021 Mar 29;31(6):631–48. doi: 10.1038/s41422-021-00491-z (PMC8169669; doi:10.1038/s41422-021-00491-z)

Fig. S11. Characterization of the physical properties of exosomes. C57BL/6J mice were intravenously injected with PBS or 5 mg/kg CMV-scrR, CMV-siR^{E} or CMV-RVG-siR^{E+T} circuit every 2 days for a total of 7 times, and then the exosomes were purified from mouse plasma and characterized by NTA, TEM and enrichment of exosomal markers. **(a-b)** Size distribution and concentration of purified exosomes determined by NTA. **(c)** Representative TEM images of purified exosomes. Scale bar: 75 nm. **(d)** Western blot analysis of specific exosomal markers (TSG101, CD63 and CD9) and plasma marker albumin in purified exosomes and whole plasma. An equal amount of total protein was loaded in each lane. Values are presented as the means ± SEM. Significance was determined using one-way ANOVA followed by Dunnett's multiple comparison for panel b. NS, not significant.

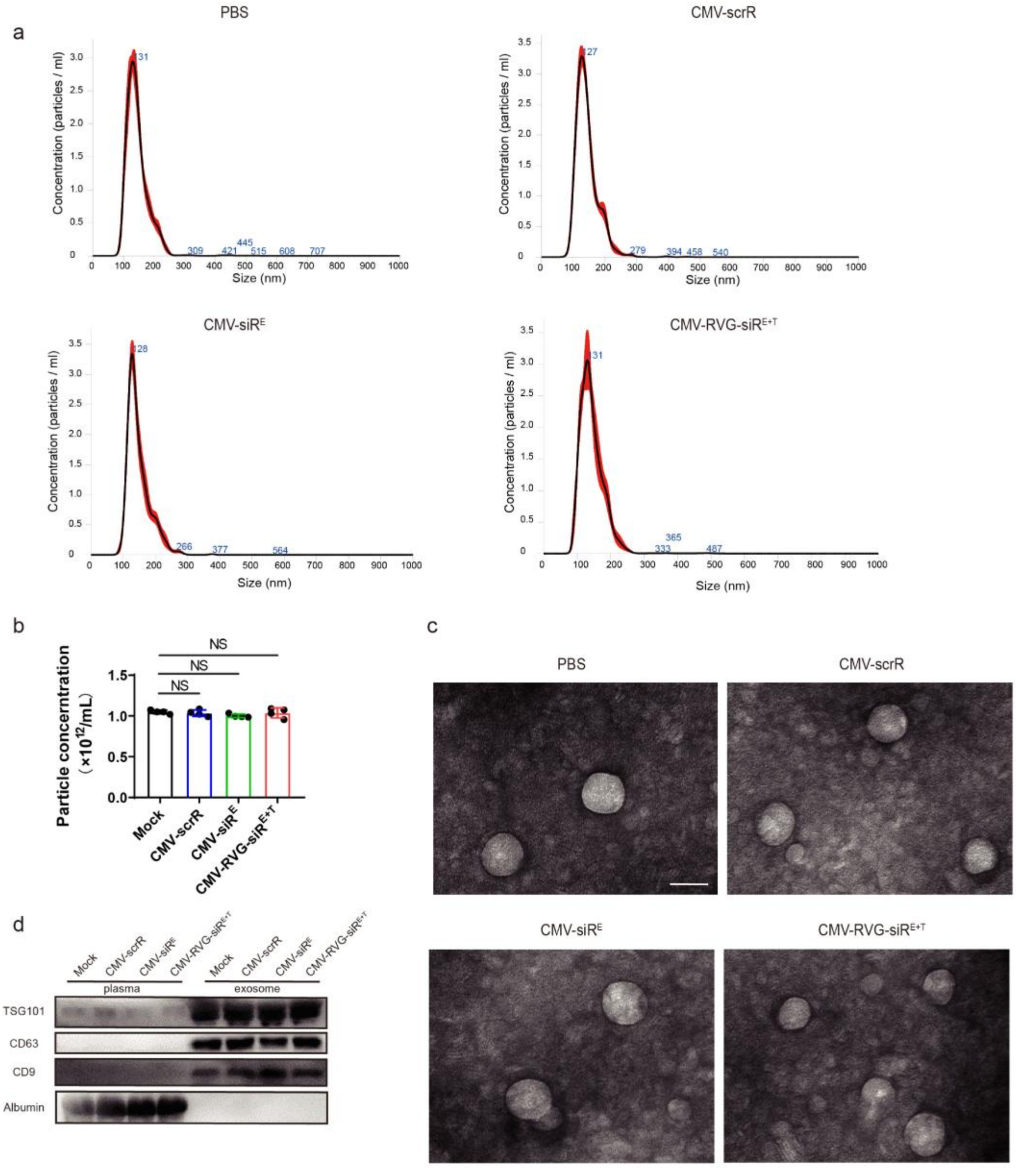

Supplement: Supplementary file 11 — Fig. S11 [file 41422_2021_491_MOESM11_ESM.pdf]
