# Supplementary material for: In vivo self-assembled small RNAs as a new generation of RNAi therapeutics
Source: Cell Res. 2021 Mar 29;31(6):631–48. doi: 10.1038/s41422-021-00491-z (PMC8169669; doi:10.1038/s41422-021-00491-z)

Fig. S12. Tissue distribution kinetics of the EGFR siRNA in various mouse tissues following tail vein injection of the genetic circuit. (a) The dynamic range and sensitivity of the quantitative RT-PCR assay for measuring EGFR siRNA. Synthetic single-stranded EGFR siRNA was serially diluted over several orders of magnitude, corresponding to levels ranging from 0.1 attomole to 1 femtomole and was assessed via quantitative RT-PCR. The resulting C_T values were plotted against the amount of input EGFR siRNA to generate a standard curve. Water was used in place of RNA as a no-template control (background) to determine the specificity of the primer set. Synthetic EGFR siRNA was consistently and efficiently amplified at C_T values ranging from 10.59 to 36.90, while the no-template control was not adequately amplified (C_T value > 40). According to the dynamic quantification range of the standard curve, the lower boundary of the detection spectrum for EGFR siRNA is 0.1 attomole (corresponding to the C_T value of 36.90). **(b)** The individual C_T values for EGFR siRNA in various mouse tissues following tail vein injection of 5 mg/kg CMV-siR^E circuit (n = 3 in each group). The C_T values of EGFR siRNA in the liver, lung, pancreas, spleen, colon, kidney, heart, brain and CD4$^+$ T cells were consistently within the linear range at all time points but were outside the linear range for skeletal muscle, as illustrated in the individual C_T -vs.-time curve. By referring to the standard curve, the concentration of EGFR siRNA in various tissues was calculated.

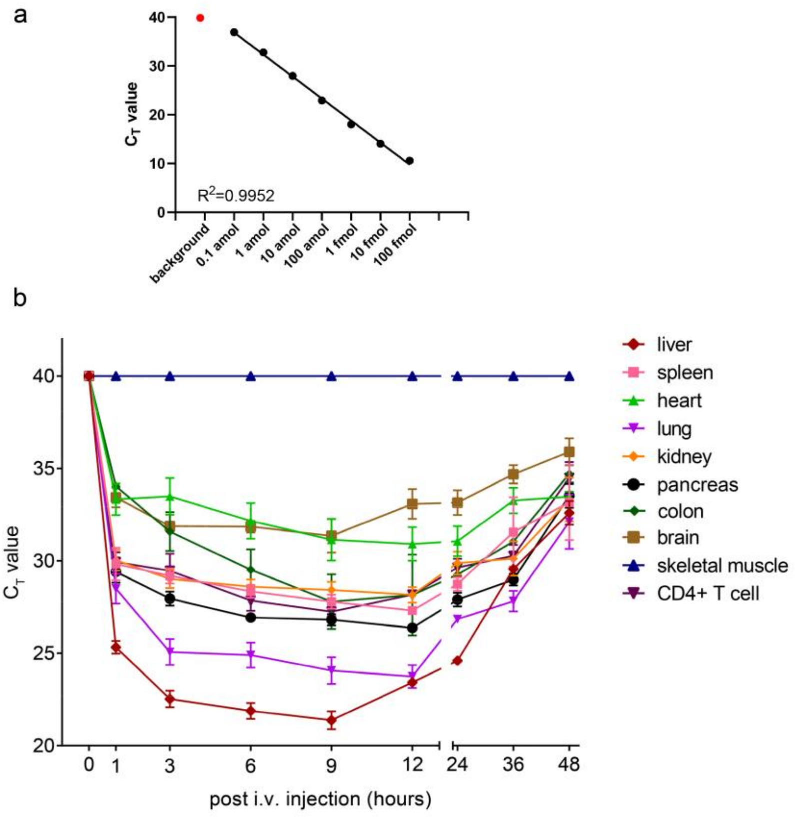

Supplement: Supplementary file 12 — Fig. S12 [file 41422_2021_491_MOESM12_ESM.pdf]
