# Supplementary material for: In vivo self-assembled small RNAs as a new generation of RNAi therapeutics
Source: Cell Res. 2021 Mar 29;31(6):631–48. doi: 10.1038/s41422-021-00491-z (PMC8169669; doi:10.1038/s41422-021-00491-z)

Fig. S13. Dose-dependent distribution of EGFR siRNA in various mouse tissues. Mice were intravenously injected with different doses (0.05, 0.5 or 5 mg/kg) of CMV-siR^{E} circuit (n = 3 in each group). At 9 hours after injection, a quantitative RT-PCR assay was performed to assess the level of EGFR siRNA in various mouse tissues. The CMV-scrR circuit was injected as a negative control. Values are presented as the means ± SEM.

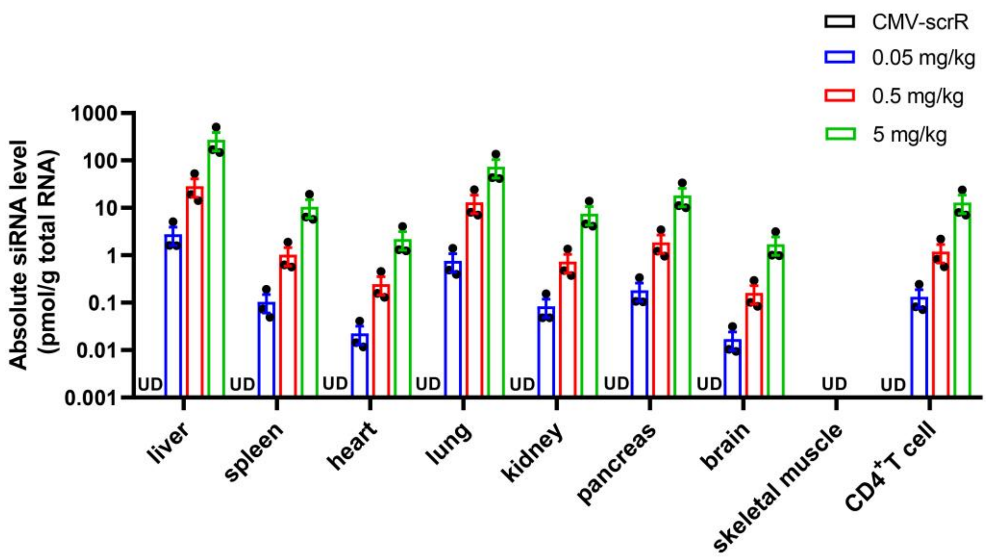

Supplement: Supplementary file 13 — Fig. S13 [file 41422_2021_491_MOESM13_ESM.pdf]
