# Supplementary material for: In vivo self-assembled small RNAs as a new generation of RNAi therapeutics
Source: Cell Res. 2021 Mar 29;31(6):631–48. doi: 10.1038/s41422-021-00491-z (PMC8169669; doi:10.1038/s41422-021-00491-z)

Fig. S14. *In situ* detection of EGFR siRNA in mouse liver and lung. Mice were intravenously injected with 5 mg/kg CMV-scrR or CMV-siR^{E} circuit for a total of 3 times. Positive *in situ* hybridization signals are shown in red, and DAPI-stained nuclei are shown in blue. Scale bar: 75 μm.

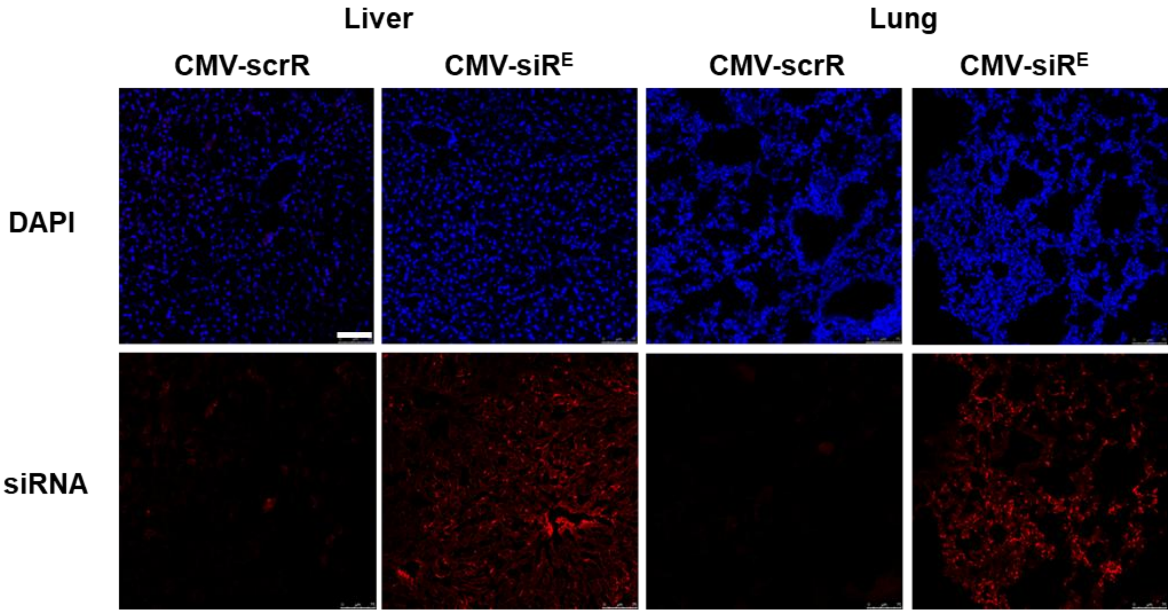

Supplement: Supplementary file 14 — Fig. S14 [file 41422_2021_491_MOESM14_ESM.pdf]
