# Supplementary material for: In vivo self-assembled small RNAs as a new generation of RNAi therapeutics
Source: Cell Res. 2021 Mar 29;31(6):631–48. doi: 10.1038/s41422-021-00491-z (PMC8169669; doi:10.1038/s41422-021-00491-z)

Fig. S15. Delivery of EGFR siRNA to various mouse tissues following injection of 5 mg/kg CMV-siR^{E} circuit through the common bile duct. (a) Kinetics of the precursor and mature form of EGFR siRNA in the mouse liver (n = 3 in each group). **(b)** Kinetics of the EGFR siRNA in mouse plasma, exosome pellet fraction and exosome-depleted supernatant fraction (n = 3 in each group). **(c)** Tissue distribution kinetics of EGFR siRNA in various mouse tissues (n = 3 in each group). Values are presented as the means ± SEM.

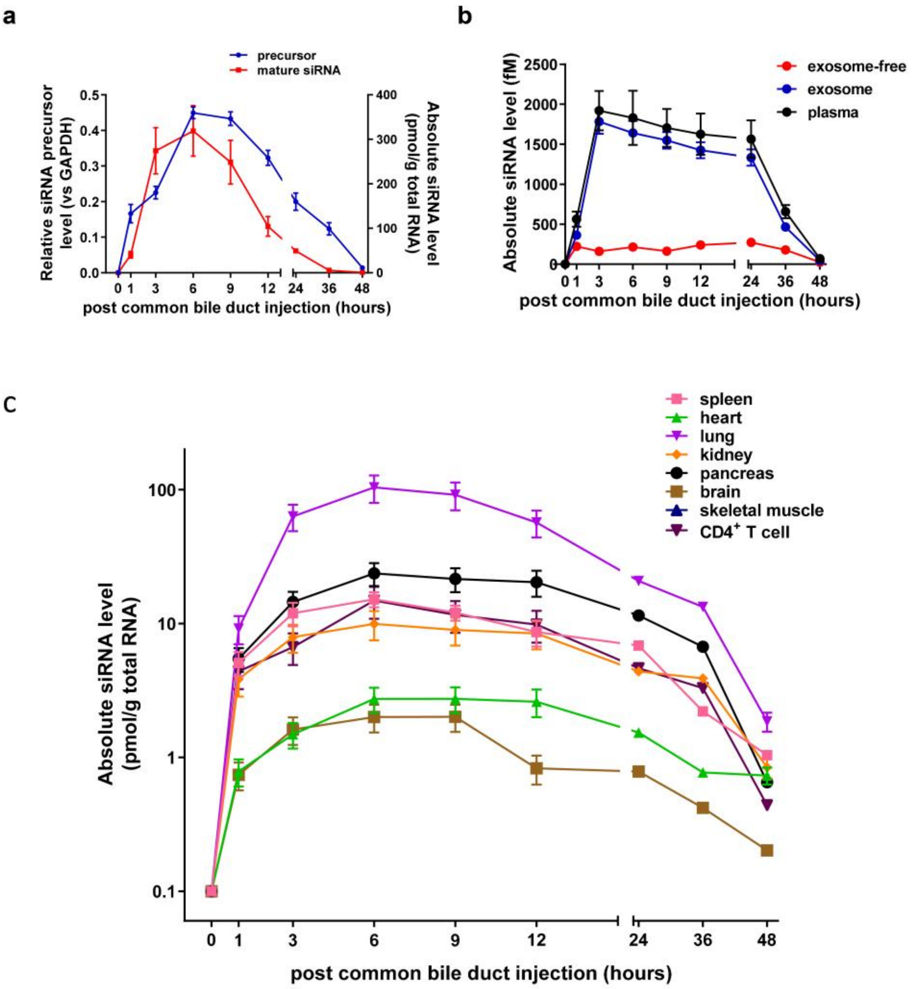

Supplement: Supplementary file 15 — Fig. S15 [file 41422_2021_491_MOESM15_ESM.pdf]
