# Supplementary material for: In vivo self-assembled small RNAs as a new generation of RNAi therapeutics
Source: Cell Res. 2021 Mar 29;31(6):631–48. doi: 10.1038/s41422-021-00491-z (PMC8169669; doi:10.1038/s41422-021-00491-z)

Fig. S16. A similar dynamics of siRNA distribution *in vivo* following tail vein injection of the CMV-siR^{E} or Alb-siR^{E} circuit. (a) The eGFP fluorescence intensity in Hepa 1-6 (a mouse hepatoma cell line) and NIH3T3 (a mouse embryonic fibroblast cell line) cells transfected with the CMV-eGFP or Alb-eGFP circuit. Scale bar: 75 μm. **(b)** Quantitative RT-PCR analysis of EGFR siRNA levels in Hepa 1-6 cells transfected with equal amounts of CMV-siR^{E} or Alb-siR^{E} circuit. **(c)** Kinetics of the EGFR siRNA in the mouse plasma following tail vein injection of 5 mg/kg CMV-siR^{E} or Alb-siR^{E} circuit (n = 3 in each group). Values are presented as the means ± SEM.

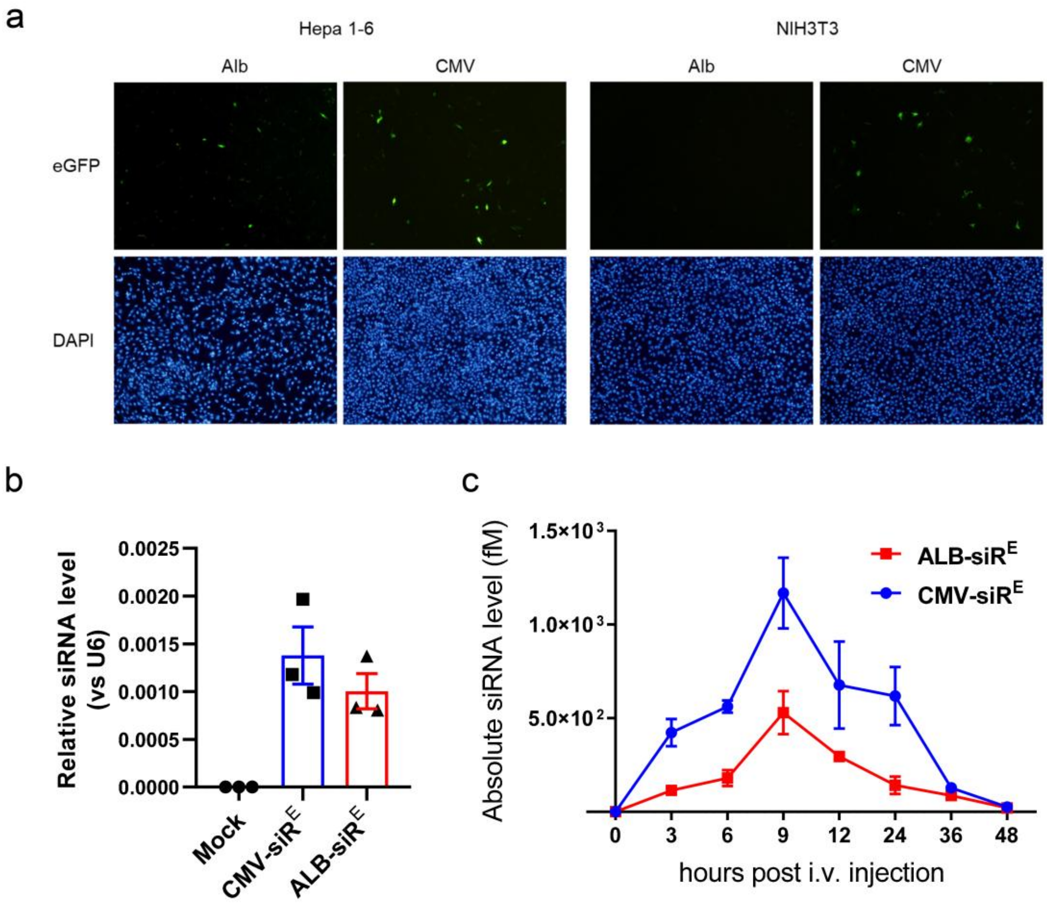

Supplement: Supplementary file 16 — Fig. S16 [file 41422_2021_491_MOESM16_ESM.pdf]
