# Supplementary material for: In vivo self-assembled small RNAs as a new generation of RNAi therapeutics
Source: Cell Res. 2021 Mar 29;31(6):631–48. doi: 10.1038/s41422-021-00491-z (PMC8169669; doi:10.1038/s41422-021-00491-z)

Fig. S17. Measurement of eGFP levels in eGFP-transgenic mice after intravenous injection of the CMV-siR^{G} circuit. The eGFP-transgenic mice were intravenously injected with 5 mg/kg CMV-scrR or CMV-siR^{G} circuit (n = 4 in each group). After 7 injections every 2 days, a quantitative analysis of the eGFP levels in the liver, spleen, heart, lung, kidney, pancreas, brain and skeletal muscle was performed by measuring the eGFP fluorescence emission in total protein from these tissues. Values are presented as the means ± SEM. Significance was determined using one-way ANOVA followed by Dunnett's multiple comparison. * $p < 0.05$; ** $p < 0.01$; NS, not significant.

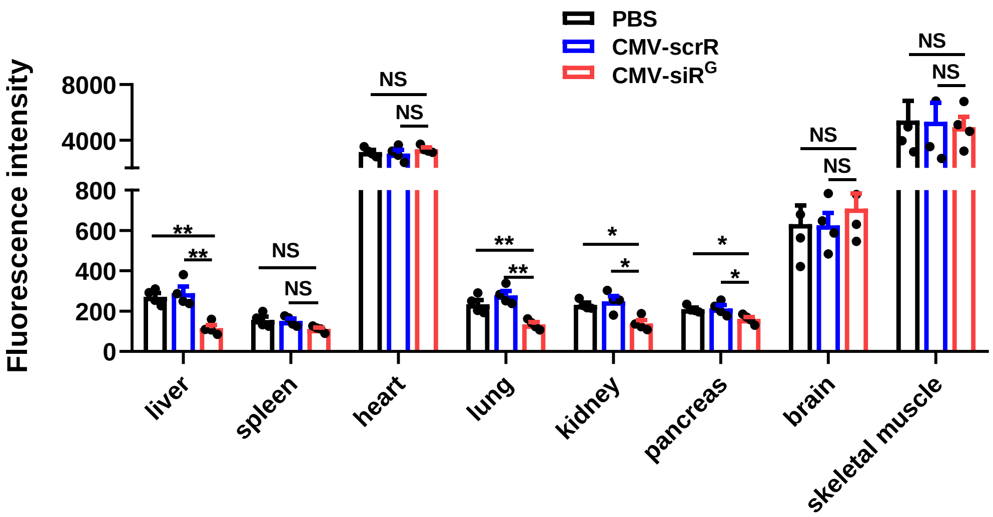

Supplement: Supplementary file 17 — Fig. S17 [file 41422_2021_491_MOESM17_ESM.pdf]
