# Supplementary material for: In vivo self-assembled small RNAs as a new generation of RNAi therapeutics
Source: Cell Res. 2021 Mar 29;31(6):631–48. doi: 10.1038/s41422-021-00491-z (PMC8169669; doi:10.1038/s41422-021-00491-z)

Fig. S18. Representative micro-CT images of mouse lungs pre- and post-treatment with the CMV-siR^E circuit in an orthotopic lung cancer model. Nude mice were intravenously injected with LLC cells and analyzed using micro-CT on day 30 post-inoculation to ensure the formation of lung tumours. Mice were then intravenously injected with PBS or 5 mg/kg CMV-scrR or CMV-siR^E circuit or intragastrically administered gefitinib every 2 days for a total of 7 times. Then, mice were monitored to determine tumour growth using micro-CT. Tumours have distinguishable X-ray densities and are defined by a yellow line in the individual CT images.

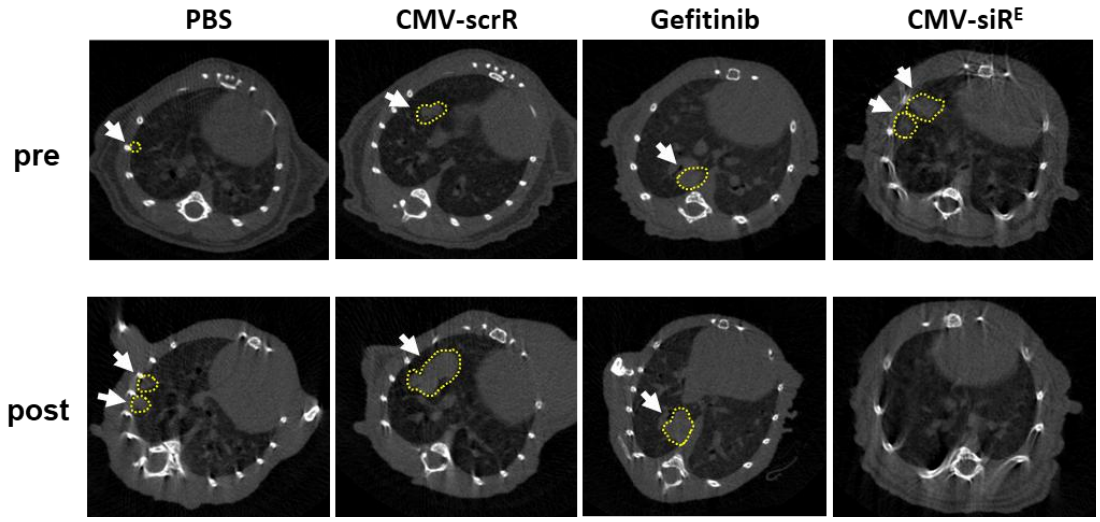

Supplement: Supplementary file 18 — Fig. S18 [file 41422_2021_491_MOESM18_ESM.pdf]
