# Supplementary material for: In vivo self-assembled small RNAs as a new generation of RNAi therapeutics
Source: Cell Res. 2021 Mar 29;31(6):631–48. doi: 10.1038/s41422-021-00491-z (PMC8169669; doi:10.1038/s41422-021-00491-z)
Supplement: Supplementary file 19 — Fig. S19 [file 41422_2021_491_MOESM19_ESM.pdf]

Fig. S19. The 3-D reconstructions of mouse lungs pre- and post-treatment with the CMV-siR^{E} circuit in an orthotopic lung cancer model. Nude mice were intravenously injected with LLC cells and analyzed using micro-CT on day 30 post-inoculation to ensure the formation of lung tumours. Mice were then intravenously injected with PBS or 5 mg/kg CMV-scrR or CMV-siR^{E} circuit or intragastrically administered gefitinib every 2 days for a total of 7 times. Then, mice were monitored using micro-CT to determine tumour growth, and raw micro-CT data were processed and reconstructed as 3-D pulmonary images. Tumours are shown in maroon to highlight their location in the 3-D reconstructions (PBS, CMV-scrR and gefitinib, n = 6; CMV-siR^{E}, n = 12).

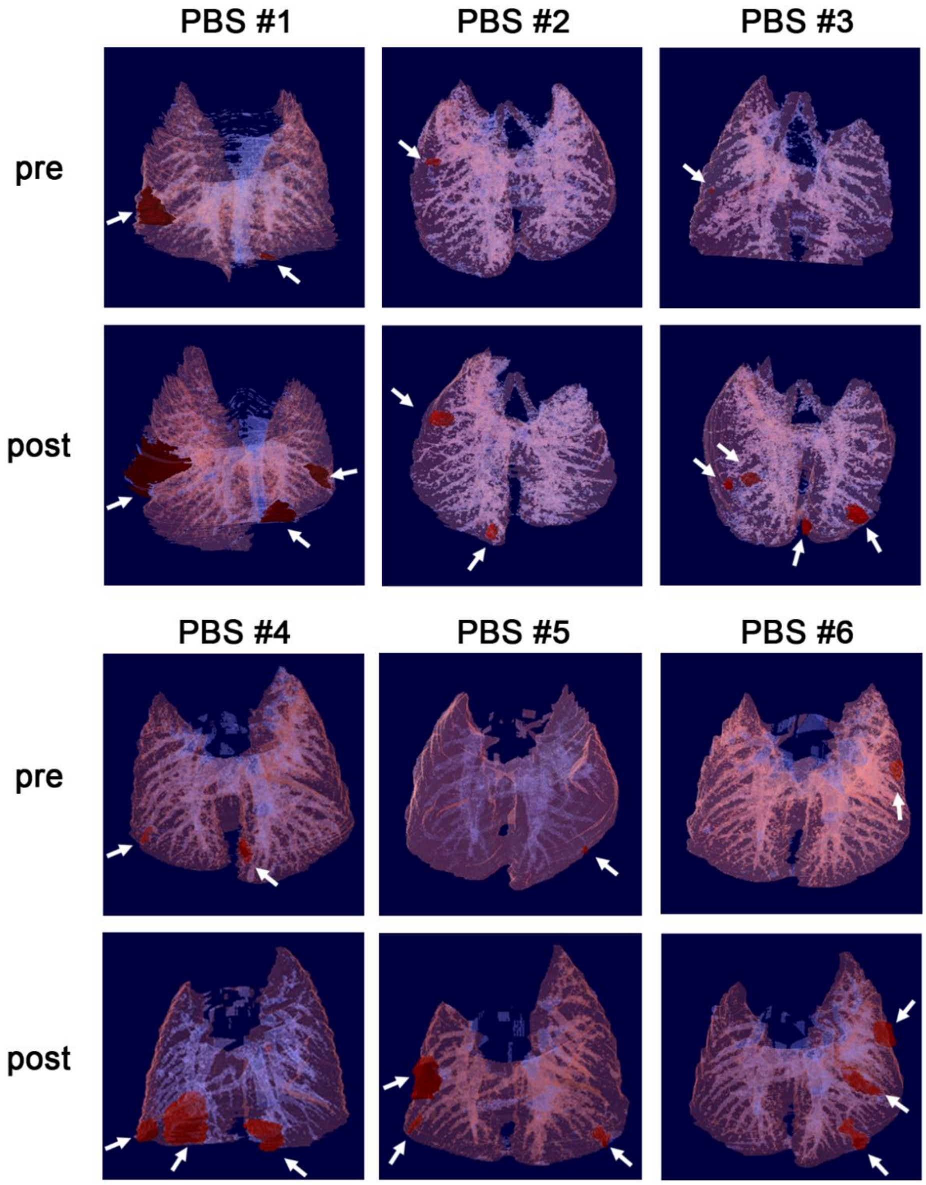

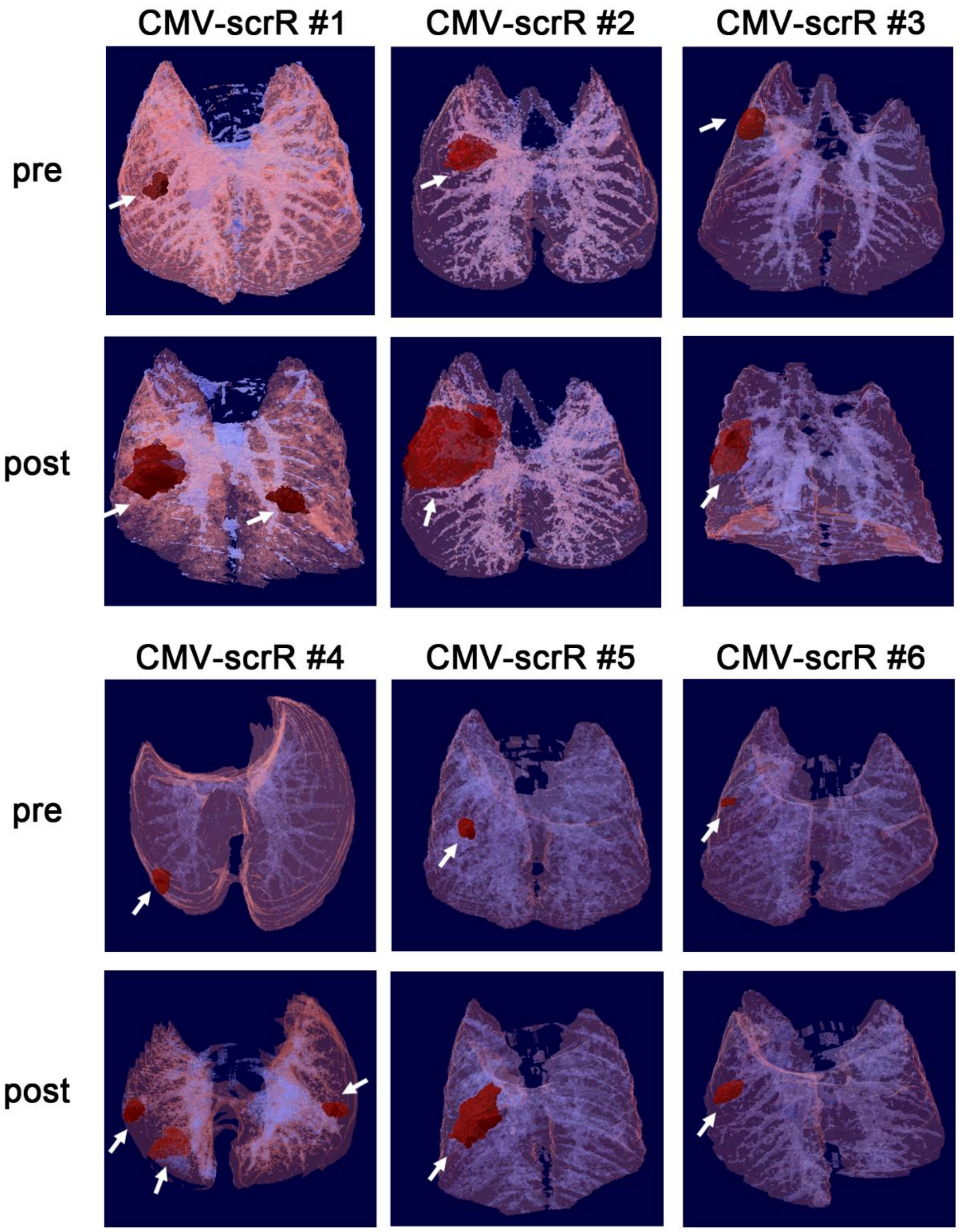

CMV-scrR #1
CMV-scrR #2
CMV-scrR #3
pre
post
CMV-scrR #4
CMV-scrR #5
CMV-scrR #6
pre
post

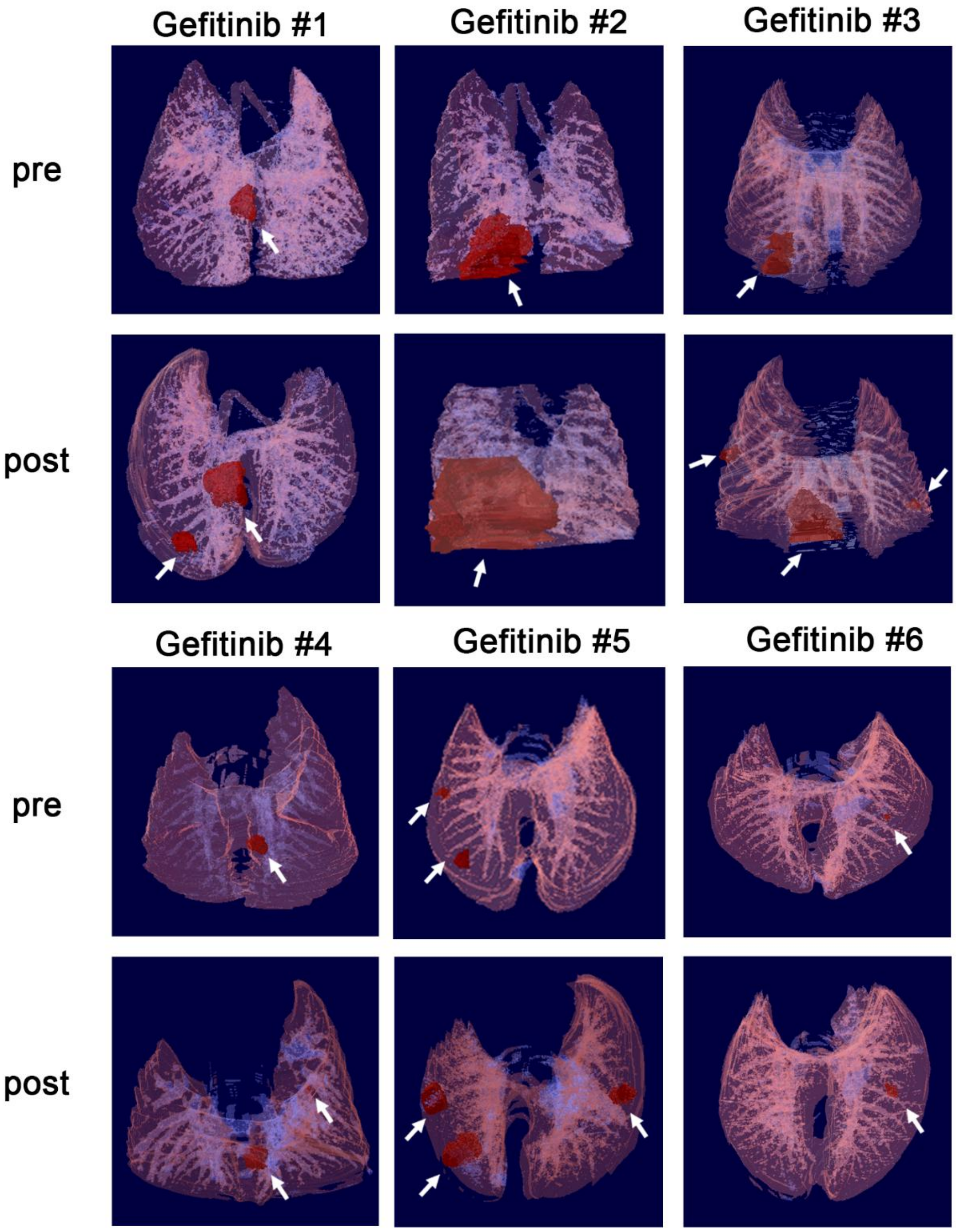
Gefitinib #1
Gefitinib #2
Gefitinib #3
pre
post
Gefitinib #4
Gefitinib #5
Gefitinib #6
pre
post

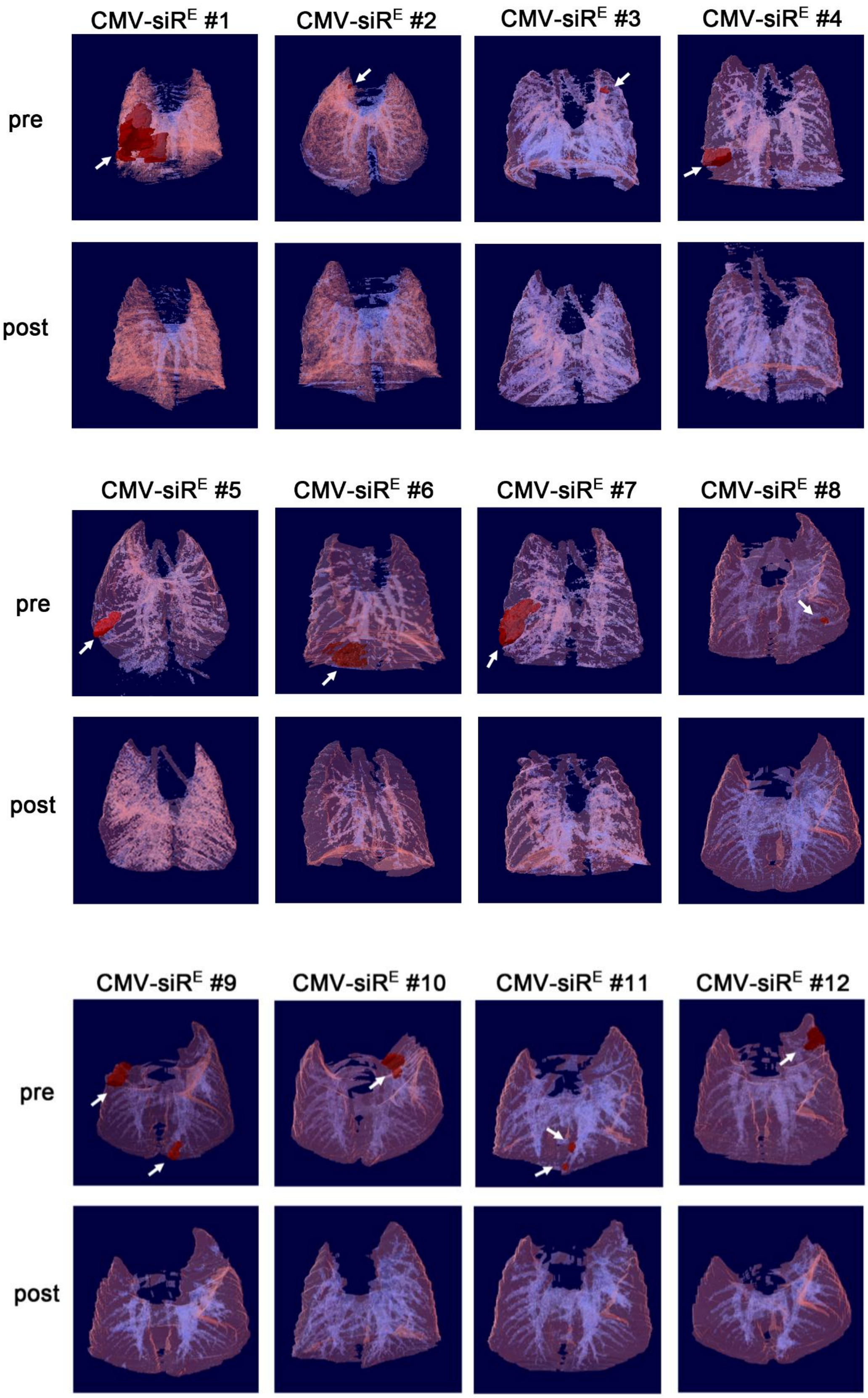
CMV-siR^E #1
CMV-siR^E #2
CMV-siR^E #3
CMV-siR^E #4
pre
post
CMV-siR^E #5
CMV-siR^E #6
CMV-siR^E #7
CMV-siR^E #8
pre
post
CMV-siR^E #9
CMV-siR^E #10
CMV-siR^E #11
CMV-siR^E #12
pre
post
