# Supplementary material for: In vivo self-assembled small RNAs as a new generation of RNAi therapeutics
Source: Cell Res. 2021 Mar 29;31(6):631–48. doi: 10.1038/s41422-021-00491-z (PMC8169669; doi:10.1038/s41422-021-00491-z)
Supplement: Supplementary file 20 — Fig. S20 [file 41422_2021_491_MOESM20_ESM.pdf]

Fig. S20. Evaluation of the toxic effects in mice following intravenous injection of genetic circuits. C57BL/6J mice were intravenously injected with PBS or 5 mg/kg CMV-scrR or CMV-siR^{E} circuit every 2 days for a total of 7 times. After treatment, mice were sacrificed, and blood and tissue samples were collected and analyzed for serum biochemical indicators, tissue damage, immunotoxicity and RNAi-associated immune activation. **(a)** Measurement of the representative serum biochemical indicators, including alanine aminotransferase (ALT), aspartate aminotransferase (AST), total bilirubin (TBIL), blood urea nitrogen (BUN), alkaline phosphatase (ALP) and creatinine (CREA), in mouse serum (n = 5 in each group). **(b)** Liver, lung, spleen and kidney tissues from treated mice were stained with H&E. No overt tissue damage was observed in circuit-treated mice. Scale bar: 75 μm. **(c)** Measurement of the weights of lymphoid organs (thymus and spleen). **(d)** Measurement of the counts of peripheral immune cells (T cell, B cell, NK cell, DC, monocyte and neutrophil). **(e)** Measurement of representative inflammatory cytokines (IL-6, TNF-α and IFN-α) in liver and plasma for RNAi-associated immune stimulation. Values are presented as the means ± SEM. Significance was determined using one-way ANOVA followed by Dunnett's multiple comparison. NS, not significant.

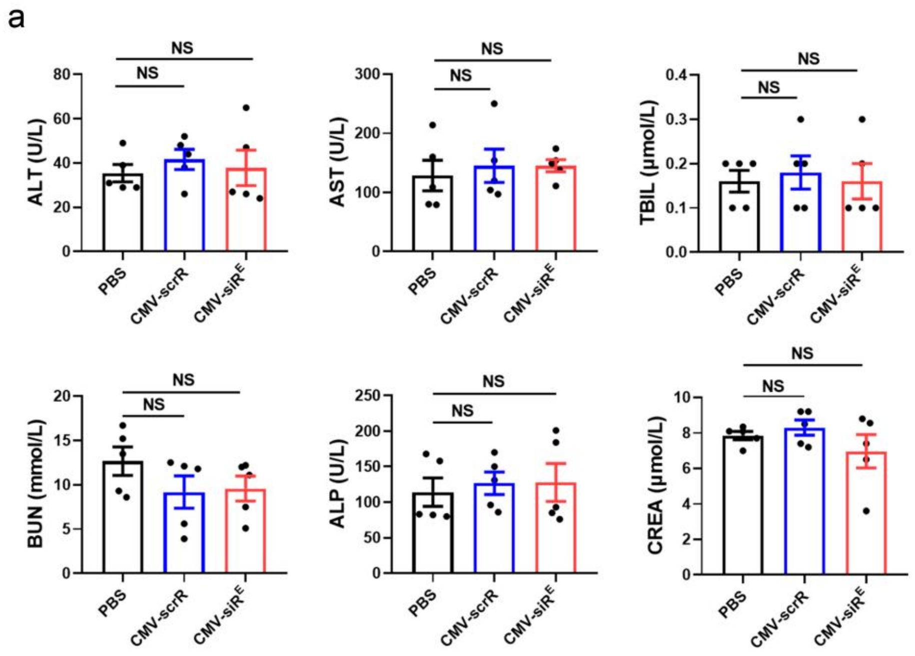

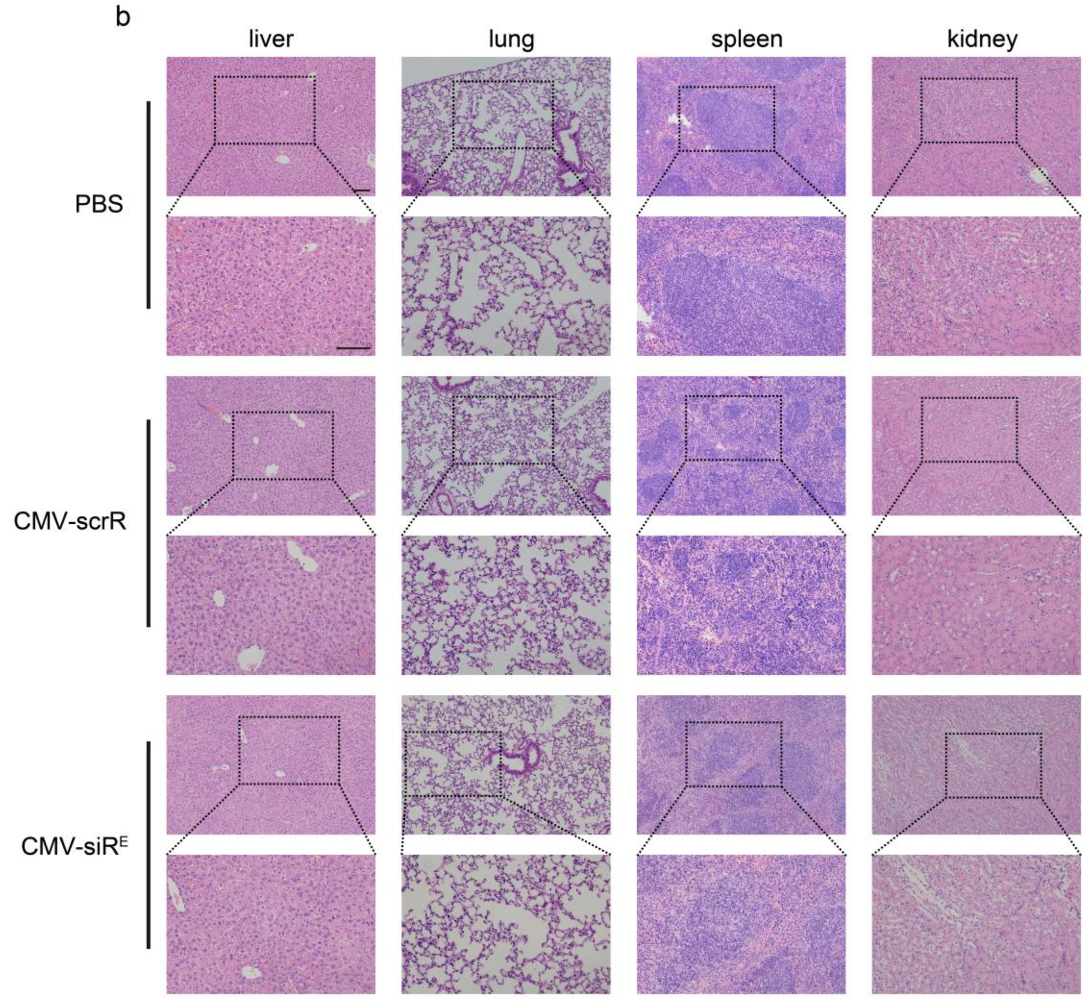
b
liver
lung
spleen
kidney
PBS
CMV-scrR
CMV-siR^E

c

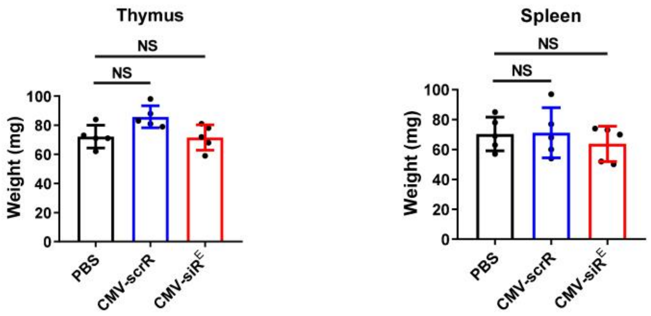
Thymus
Spleen
NS
Weight (mg)
PBS
CMV-scrR
CMV-siR^E

d

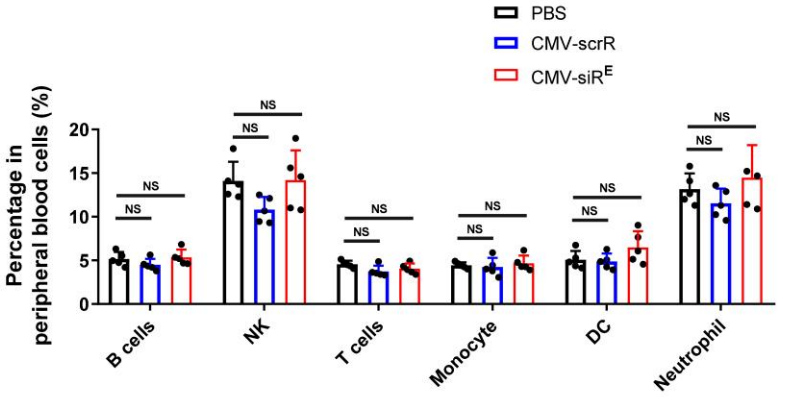
PBS
CMV-scrR
CMV-siR^E
NS
Percentage in peripheral blood cells (%)
B cells
NK
T cells
Monocyte
DC
Neutrophil

e

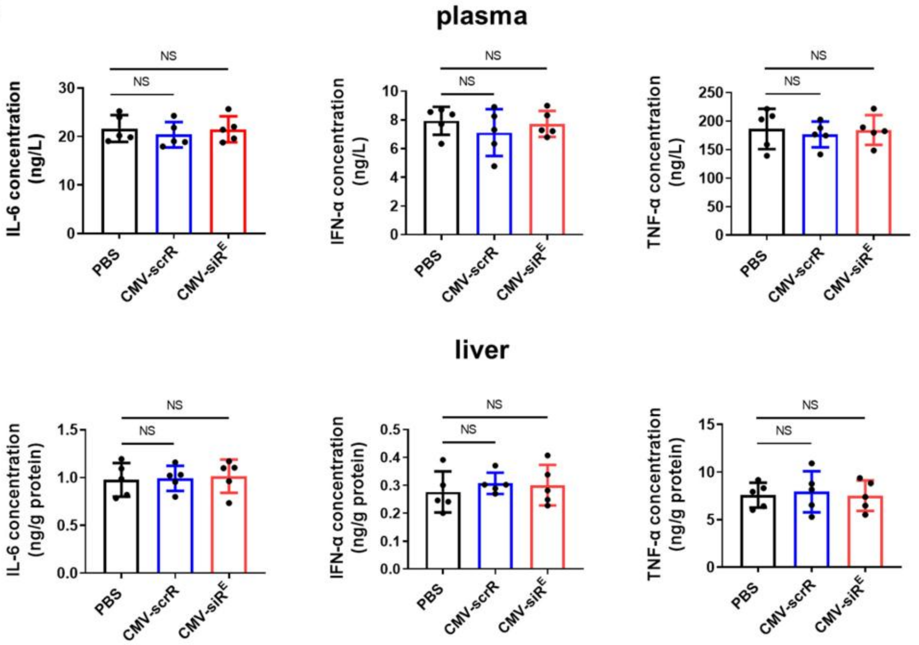
plasma
liver
NS
IL-6 concentration (ng/L)
IFN-α concentration (ng/L)
TNF-α concentration (ng/L)
IL-6 concentration (ng/g protein)
IFN-α concentration (ng/g protein)
TNF-α concentration (ng/g protein)
PBS
CMV-scrR
CMV-siR^E
