# Supplementary material for: In vivo self-assembled small RNAs as a new generation of RNAi therapeutics
Source: Cell Res. 2021 Mar 29;31(6):631–48. doi: 10.1038/s41422-021-00491-z (PMC8169669; doi:10.1038/s41422-021-00491-z)

Fig. S21. Representative micro-CT images of mouse lungs pre- and post-treatment with the CMV-siR^K circuit in a spontaneous lung cancer model. The *KRAS*$^{LSL\text{-}G12D}$; *p53*$^{fl/fl}$ mice were administered Adeno-Cre and analyzed using micro-CT on day 50 post-inhalation to ensure spontaneous tumour formation in the lungs. Mice were then intravenously injected with 5 mg/kg CMV-scrR or CMV-siR^K circuit every 2 days for a total of 7 injections. Then, mice were monitored to determine tumour growth using micro-CT. Tumours have distinguishable X-ray densities and are defined by a yellow line in the individual CT images shown here.

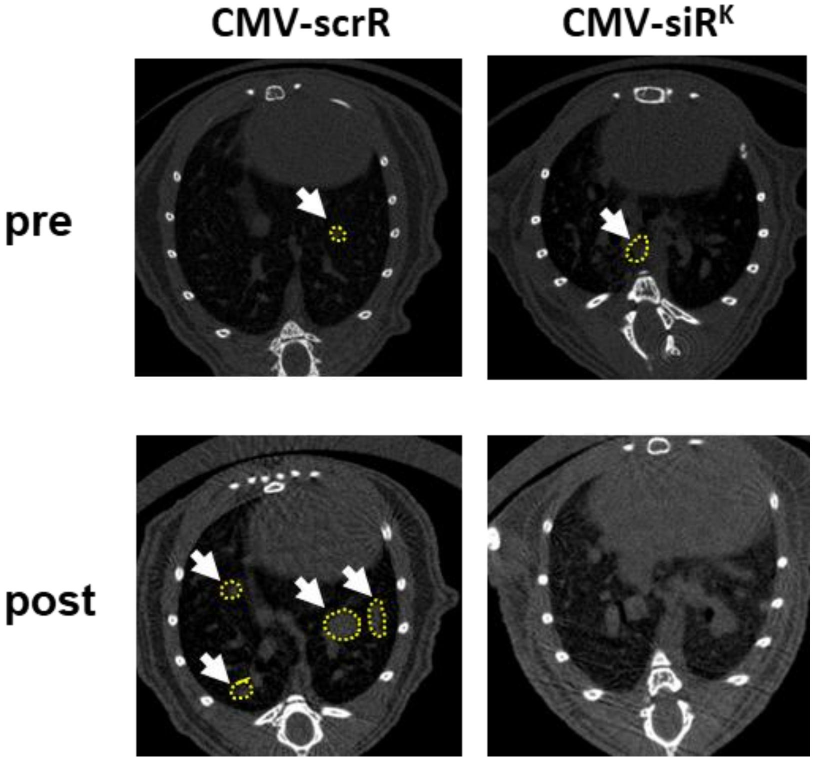

Supplement: Supplementary file 21 — Fig. S21 [file 41422_2021_491_MOESM21_ESM.pdf]
