# Supplementary material for: In vivo self-assembled small RNAs as a new generation of RNAi therapeutics
Source: Cell Res. 2021 Mar 29;31(6):631–48. doi: 10.1038/s41422-021-00491-z (PMC8169669; doi:10.1038/s41422-021-00491-z)
Supplement: Supplementary file 22 — Fig. S22 [file 41422_2021_491_MOESM22_ESM.pdf]

Fig. S22. The 3-D reconstructions of mouse lungs pre- and post-treatment with the CMV-siR^K circuit in a spontaneous lung cancer model. The *KRAS*$^{LSL-G12D}$*; p53*$^{fl/fl}$ mice were administered Adeno-Cre and analyzed using micro-CT on day 50 post-inhalation to ensure spontaneous tumour formation in the lungs. Mice were then intravenously injected with 5 mg/kg CMV-scrR or CMV-siR^K circuit every 2 days for a total of 7 injections. Then, mice were monitored using micro-CT to determine tumour growth, and raw micro-CT data were processed and reconstructed as 3-D pulmonary images. Tumours are shown in maroon to highlight their location in the 3-D reconstructions (CMV-scrR, n =7; CMV-siR^K, n = 8).

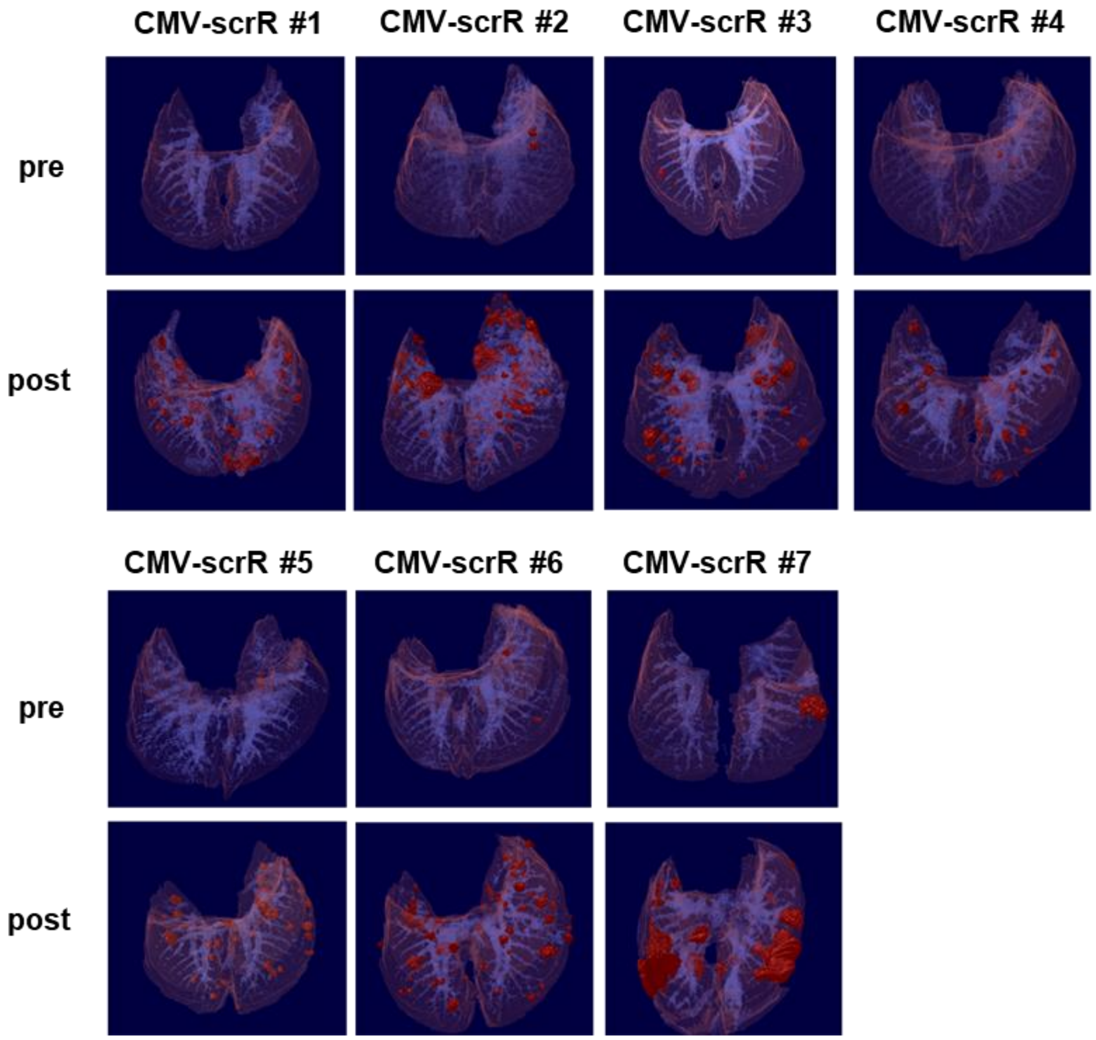

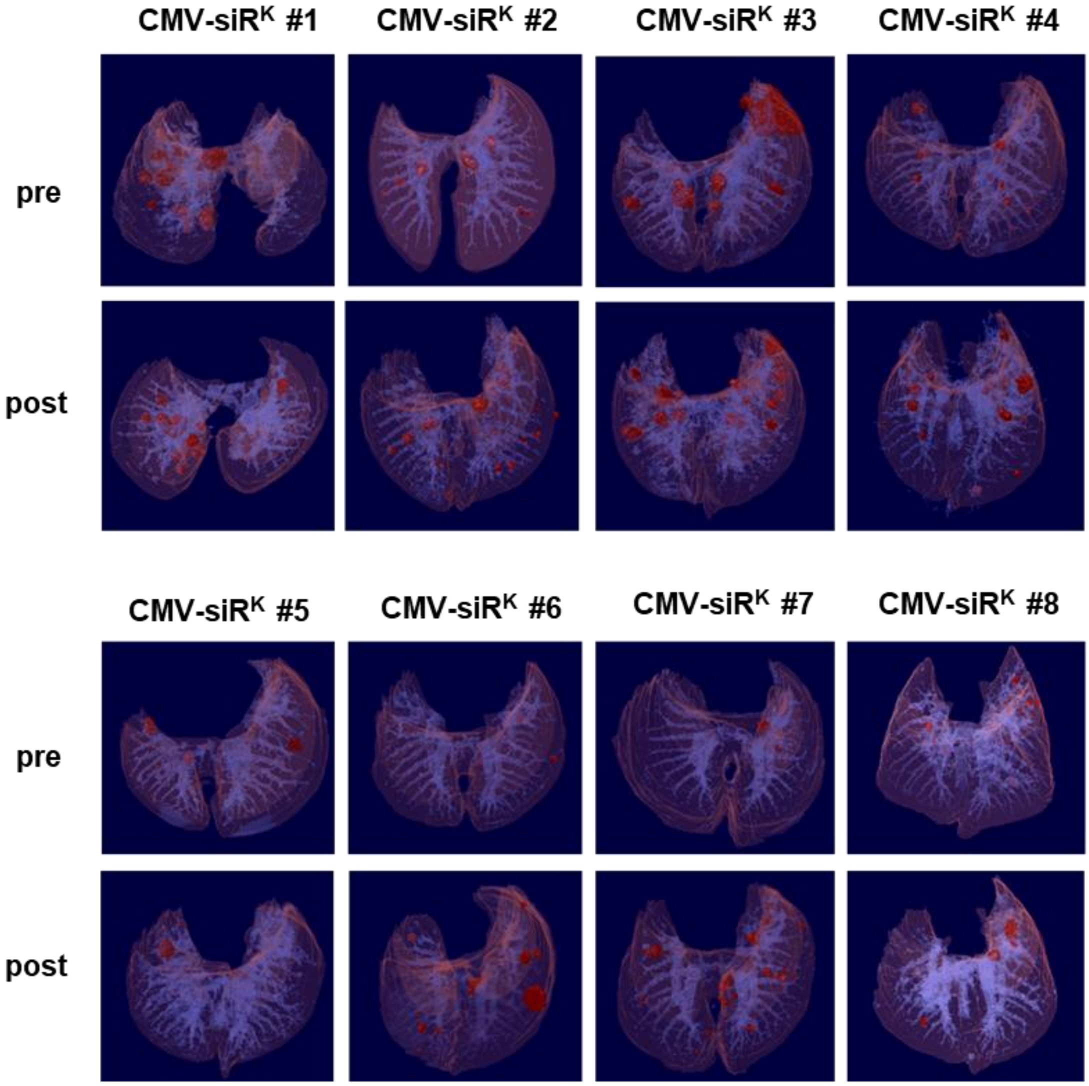

CMV-siRK #1
CMV-siRK #2
CMV-siRK #3
CMV-siRK #4
pre
post
CMV-siRK #5
CMV-siRK #6
CMV-siRK #7
CMV-siRK #8
pre
post
