# Supplementary material for: In vivo self-assembled small RNAs as a new generation of RNAi therapeutics
Source: Cell Res. 2021 Mar 29;31(6):631–48. doi: 10.1038/s41422-021-00491-z (PMC8169669; doi:10.1038/s41422-021-00491-z)

Fig. S23. Histopathological and immunohistochemical examination of mouse lungs posttreatment with the CMV-siR^{K} circuit in a spontaneous lung cancer model. The *KRAS*$^{LSL\text{-}G12D}$*; p53*$^{fl/fl}$ mice were administered Adeno-Cre and analyzed using micro-CT 50 days postinhalation to ensure spontaneous tumor formation in the lungs. Mice were then intravenously injected with 5 mg/kg CMV-scrR or CMV-siR^{K} circuit every 2 days for a total of 7 injections. After the determination of tumor growth using micro-CT, mice were sacrificed, and lung tumors were analyzed for siRNA level, proliferation and apoptosis. **(a)** KRAS siRNA levels in the plasma and plasma exosome pellets 9 hours after the final injection (n = 3 in each group). **(b)** Immunohistochemical staining of PCNA and TUNEL in lung sections. The tumor cell proliferation rate is indicated by the percentage of PCNA-positive cells, and the cell apoptosis rate is indicated by the percentage of TUNEL-positive cells. Left panel: representative images. Right panel: quantitative analysis (CMV-scrR, n = 3; CMV-siR^{K}, n = 4). Values are presented as the means ± SEM. Significance was determined using one-way ANOVA followed by Dunnett's multiple comparison. ** $p < 0.01$.

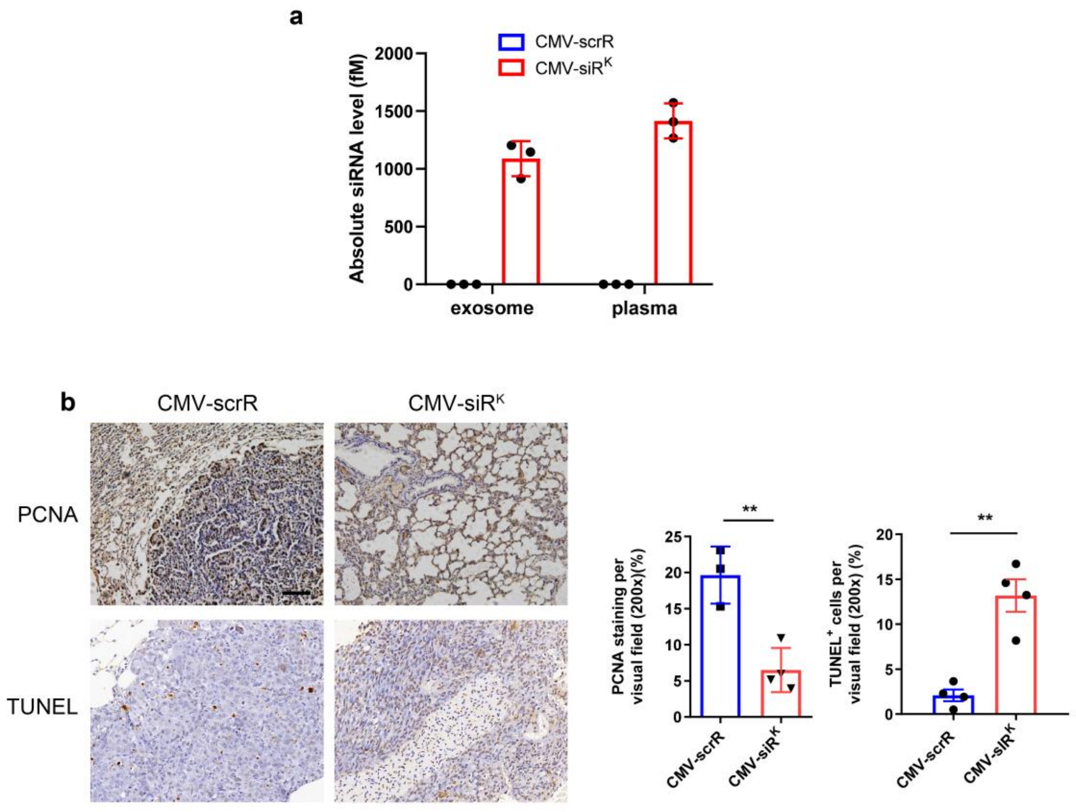

Supplement: Supplementary file 23 — Fig. S23 [file 41422_2021_491_MOESM23_ESM.pdf]
