# Supplementary material for: In vivo self-assembled small RNAs as a new generation of RNAi therapeutics
Source: Cell Res. 2021 Mar 29;31(6):631–48. doi: 10.1038/s41422-021-00491-z (PMC8169669; doi:10.1038/s41422-021-00491-z)
Supplement: Supplementary file 24 — Fig. S24 [file 41422_2021_491_MOESM24_ESM.pdf]

Fig. S24. Direct visualization of the suppression of eGFP fluorescence levels *in vivo* by self-assembled eGFP siRNA. The eGFP-transgenic mice were intravenously injected with PBS or 5 mg/kg CMV-siR^{G} or CMV-RVG-siR^{G} circuit. Twenty-four hours after treatment, mice were sacrificed, and eGFP fluorescence levels were assessed in frozen sections of brain. Shown are representative fluorescence microscopy images. Positive eGFP signals are shown in green, and DAPI-stained nuclei are shown in blue. Scale bar: 75 μm.

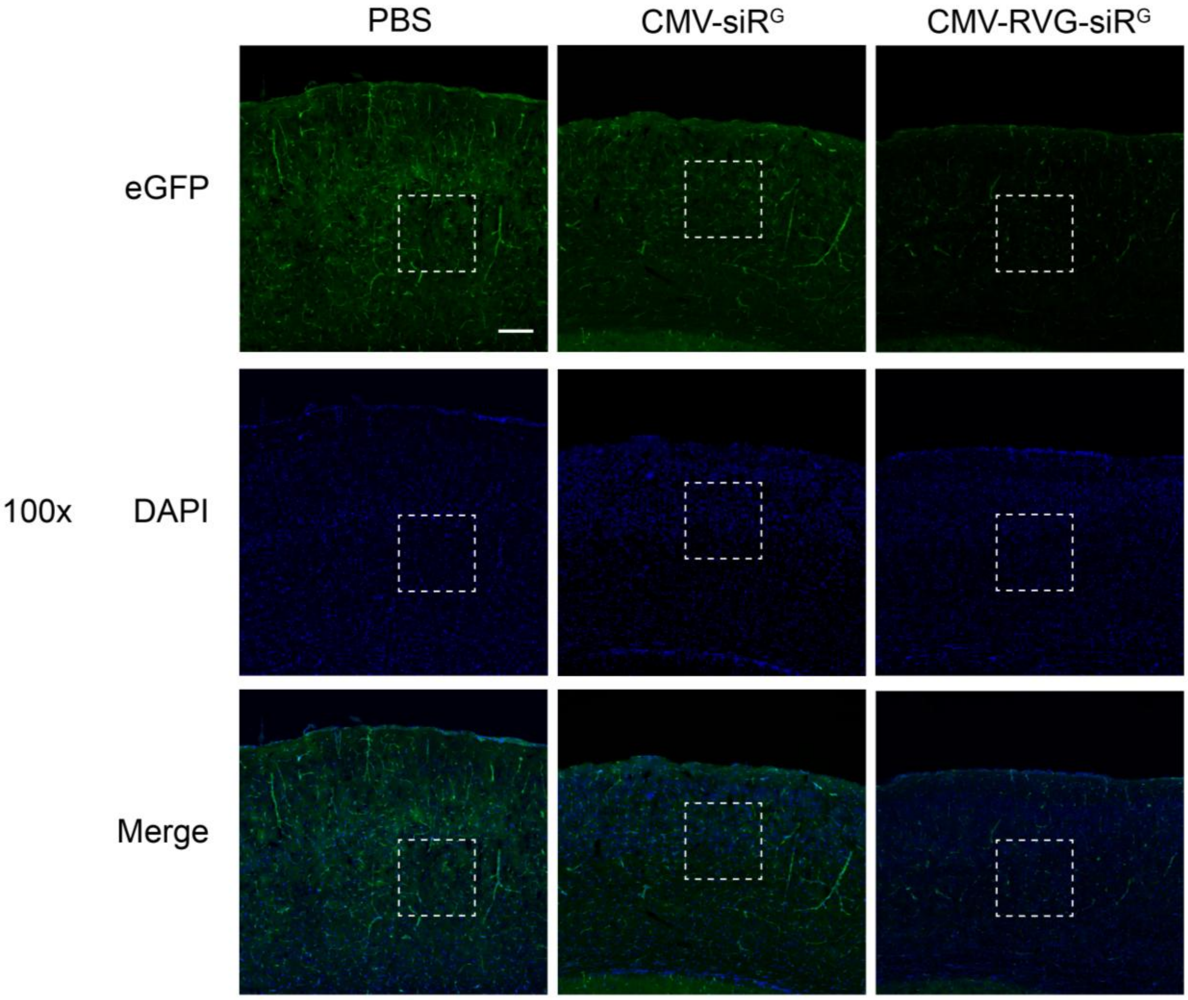

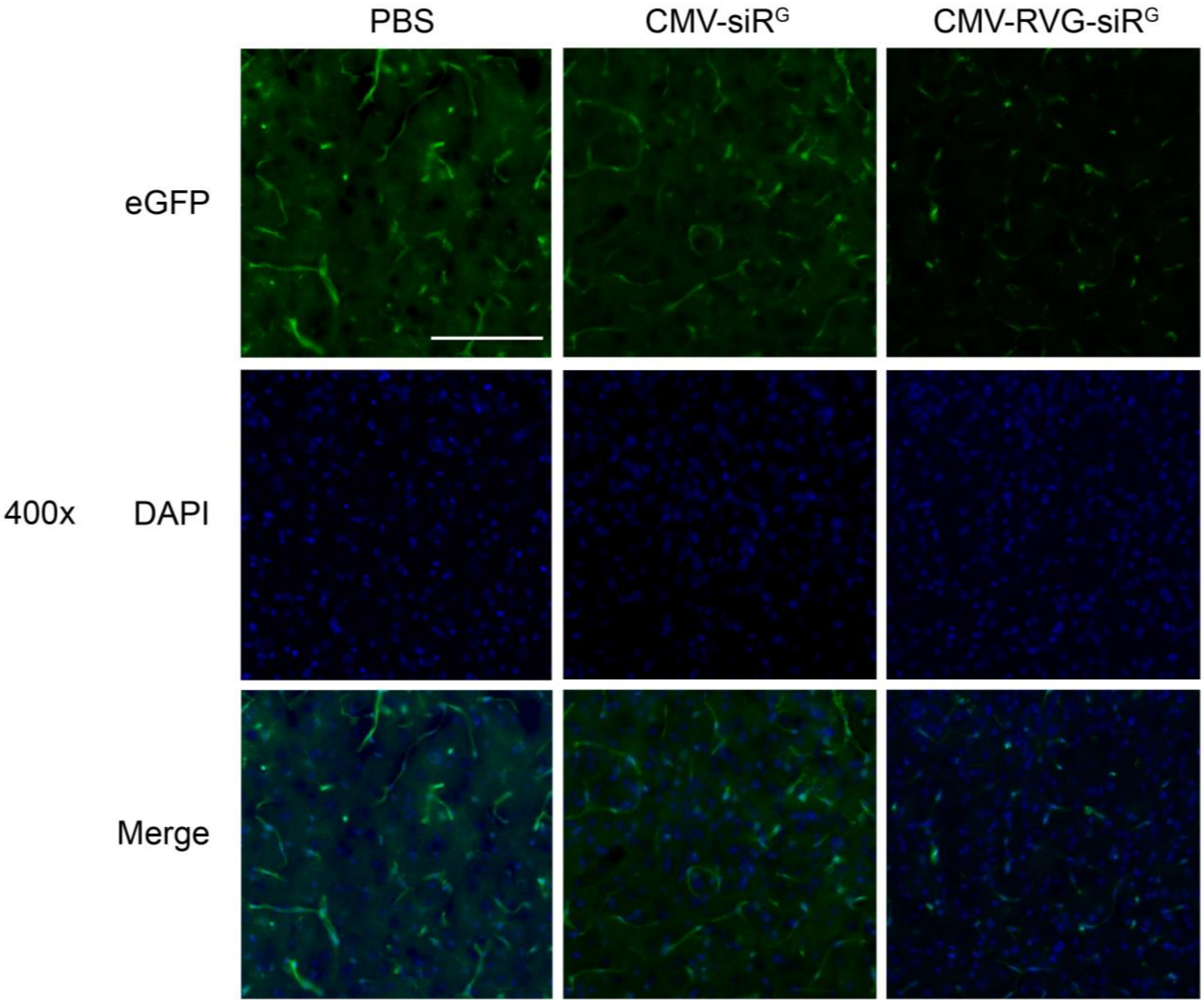
PBS
CMV-siR^{G}
CMV-RVG-siR^{G}
eGFP
400x
DAPI
Merge
