# Supplementary material for: In vivo self-assembled small RNAs as a new generation of RNAi therapeutics
Source: Cell Res. 2021 Mar 29;31(6):631–48. doi: 10.1038/s41422-021-00491-z (PMC8169669; doi:10.1038/s41422-021-00491-z)

Fig. S25. Representative fluorescence microscopy images showing the eGFP levels in mouse brain after intravenous injection with a circuit expressing a Lamp2b-eGFP dual fusion protein or an RVG-Lamp2b-eGFP triple-fusion protein. Positive eGFP signals are visualized in green, and DAPI-stained nuclei are shown in blue. Scale bar: 100 μm.

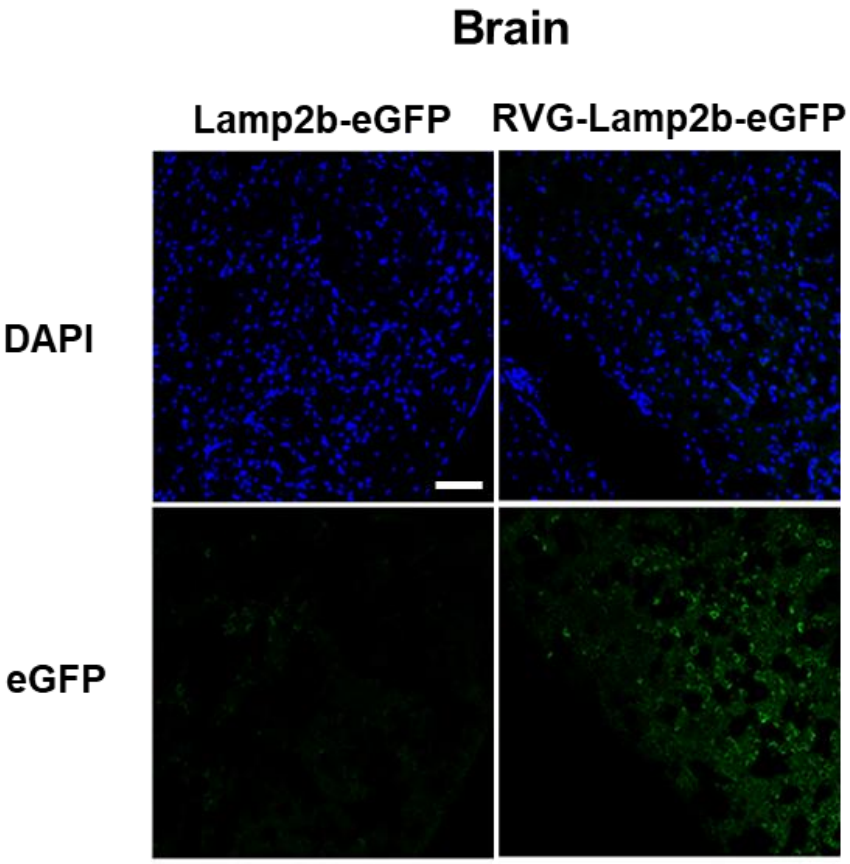

Supplement: Supplementary file 25 — Fig. S25 [file 41422_2021_491_MOESM25_ESM.pdf]
