# Supplementary material for: In vivo self-assembled small RNAs as a new generation of RNAi therapeutics
Source: Cell Res. 2021 Mar 29;31(6):631–48. doi: 10.1038/s41422-021-00491-z (PMC8169669; doi:10.1038/s41422-021-00491-z)

Fig. S26. Immunohistochemical examination of mouse glioblastomas post-treatment with the CMV-RVG-siR^{E+T} circuit in a glioblastoma mouse model. Nude mice were intracranially implanted with bioluminescent U87MG-Luc cells and analyzed using BLI on day 7 post-implantation to ensure glioblastoma formation in the brain. Mice were then intravenously injected with PBS or 5 mg/kg CMV-scrR, CMV-RVG-siR^{E} or CMV-RVG-siR^{E+T} circuit for a total of 7 times over 2 weeks. After determination of tumour growth using BLI, the mice were sacrificed, and the glioblastomas were analyzed using immunohistochemical staining. **(a)** Representative EGFR-, TNC- and PCNA-stained glioblastoma sections. Scale bar: 75 μm. **(b)** Quantitative analysis of EGFR, TNC and PCNA levels in glioblastoma sections (n = 3 in each group). The tumour cell proliferation rate is indicated by the percentage of PCNA-positive cells. Values are presented as the means ± SEM. Significance was determined using one-way ANOVA followed by Dunnett's multiple comparison. * $p < 0.05$; ** $p < 0.01$; *** $p < 0.005$; NS, not significant.

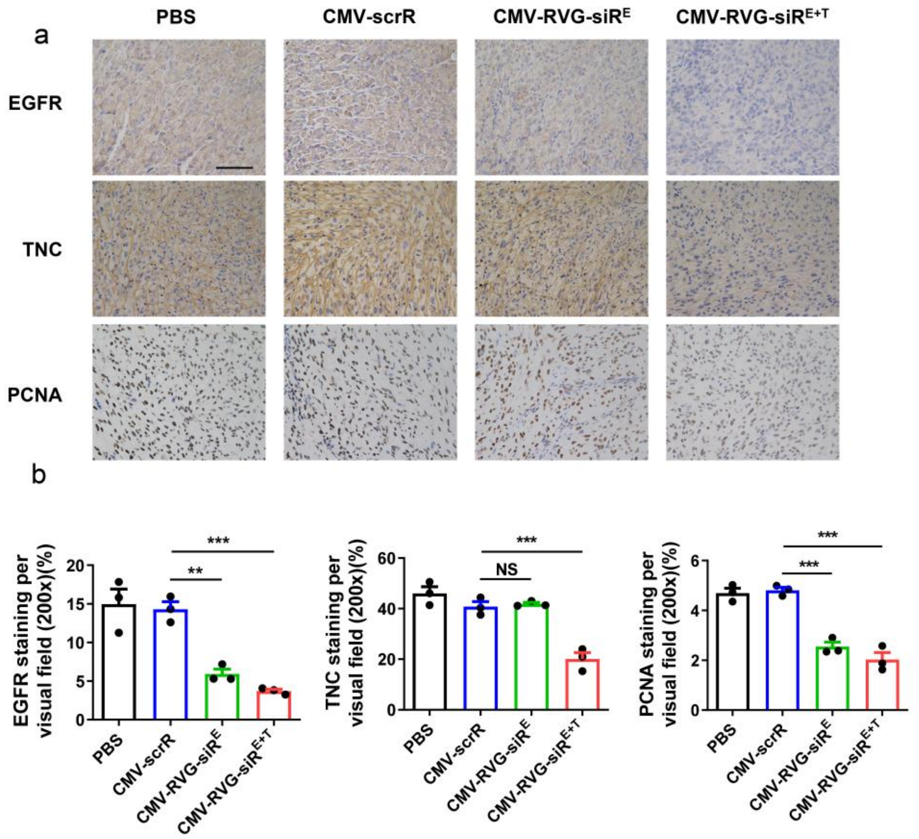

Supplement: Supplementary file 26 — Fig. S26 [file 41422_2021_491_MOESM26_ESM.pdf]
