# Supplementary material for: In vivo self-assembled small RNAs as a new generation of RNAi therapeutics
Source: Cell Res. 2021 Mar 29;31(6):631–48. doi: 10.1038/s41422-021-00491-z (PMC8169669; doi:10.1038/s41422-021-00491-z)
Supplement: Supplementary file 27 — Fig. S27 [file 41422_2021_491_MOESM27_ESM.pdf]

Fig. S27. Delivery of PTP1B siRNA to the hypothalamus by intravenous injection of the CMV-RVG-siR^P circuit. (a-b) *In situ* signals of PTP1B siRNA in the mouse liver and hypothalamus. Male mice at 3 months of age were intravenously injected with 5 mg/kg CMV-scrR, CMV-siR^P or CMV-RVG-siR^P circuit 3 times over 3 days. Positive *in situ* hybridization signals are shown in green, and DAPI-stained nuclei are shown in blue. Scale bar: 50 μm. **(c)** Immunofluorescence staining of PTP1B and in mouse hypothalamus. Male C57BL/6J mice at 3 months of age were intravenously injected with 5 mg/kg CMV-scrR, CMV-siR^P or CMV-RVG-siR^P circuit for a total of 5 times over 10 days. Shown are equivalent hypothalamic sections stained for PTP1B (green) and DAPI (blue). Scale bar: 100 μm.

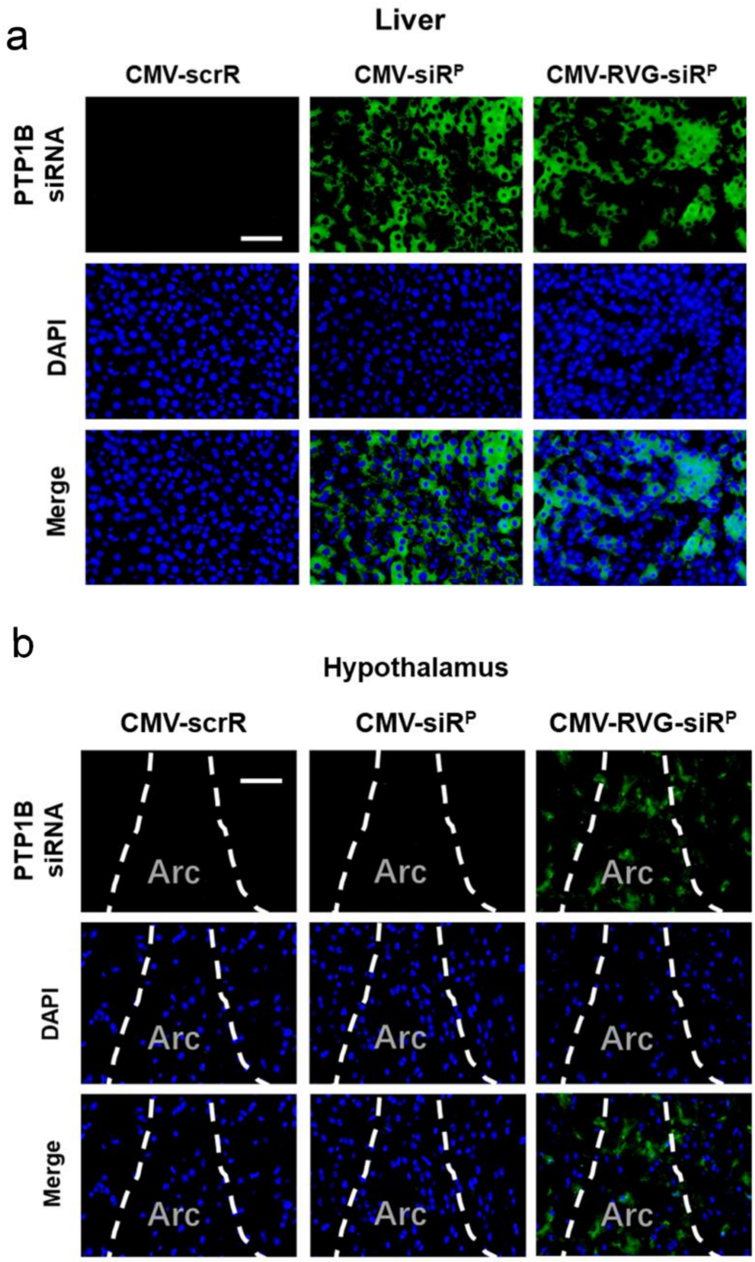

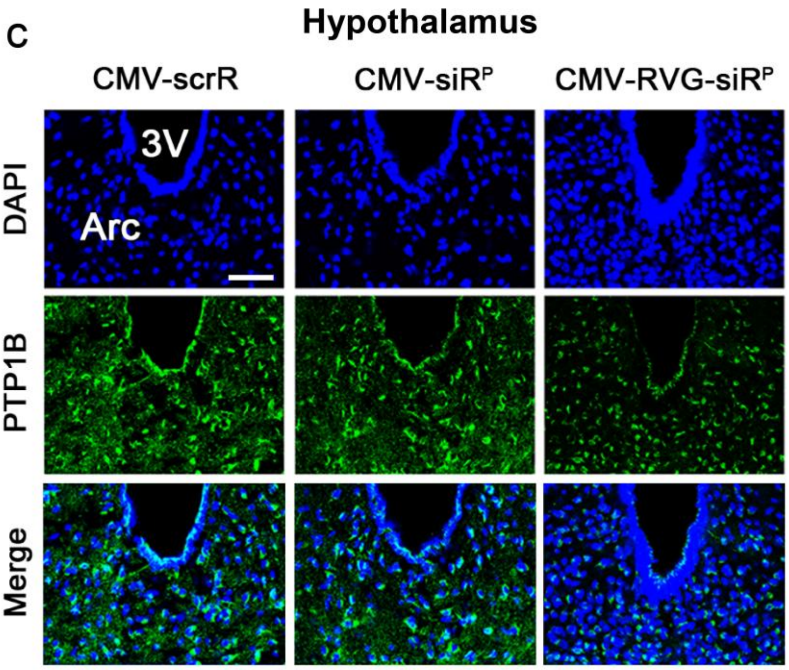
C
Hypothalamus
CMV-scrR
CMV-siR^P
CMV-RVG-siR^P
DAPI
PTP1B
Merge
3V
Arc
