# Supplementary material for: In vivo self-assembled small RNAs as a new generation of RNAi therapeutics
Source: Cell Res. 2021 Mar 29;31(6):631–48. doi: 10.1038/s41422-021-00491-z (PMC8169669; doi:10.1038/s41422-021-00491-z)

Fig. S28. Intravenous injection of the CMV-RVG-siR^P circuit decreases adiposity and increases energy expenditure in HFD model of obesity. Male C57BL/6J mice at 3 weeks of age were placed on a HFD for 12 weeks. Mice rapidly gained weight and became obese. Mice were then maintained on a HFD and treated with PBS or 5 mg/kg CMV-scrR, CMV-siR^P or CMV-RVG-siR^P circuit through tail vein injection for a total of 12 times over 24 days. After treatment, metabolic parameters and energy expenditure were evaluated. **(a-c)** Determination of triglyceride (TG), total cholesterol (TC) and low-density lipoprotein (LDL) levels in serum assessed by an enzymatic colorimetric method (n = 13-14 in each group). **(d)** Body length (n = 14 in each group). **(e)** Food intake (n = 14 in each group). **(f)** Western blot analysis of PTP1B in the hypothalamus and liver. Mice were injected with leptin and collected for hypothalamic or injected with insulin and collected for liver. Tissues were processed for immunoblot analysis of PTP1B. Shown is the quantitative analysis (n = 3 in each group). Significance was determined using one-way ANOVA followed by Dunnett's multiple comparison. * $p < 0.05$; ** $p < 0.01$; *** $p < 0.005$; NS, not significant.

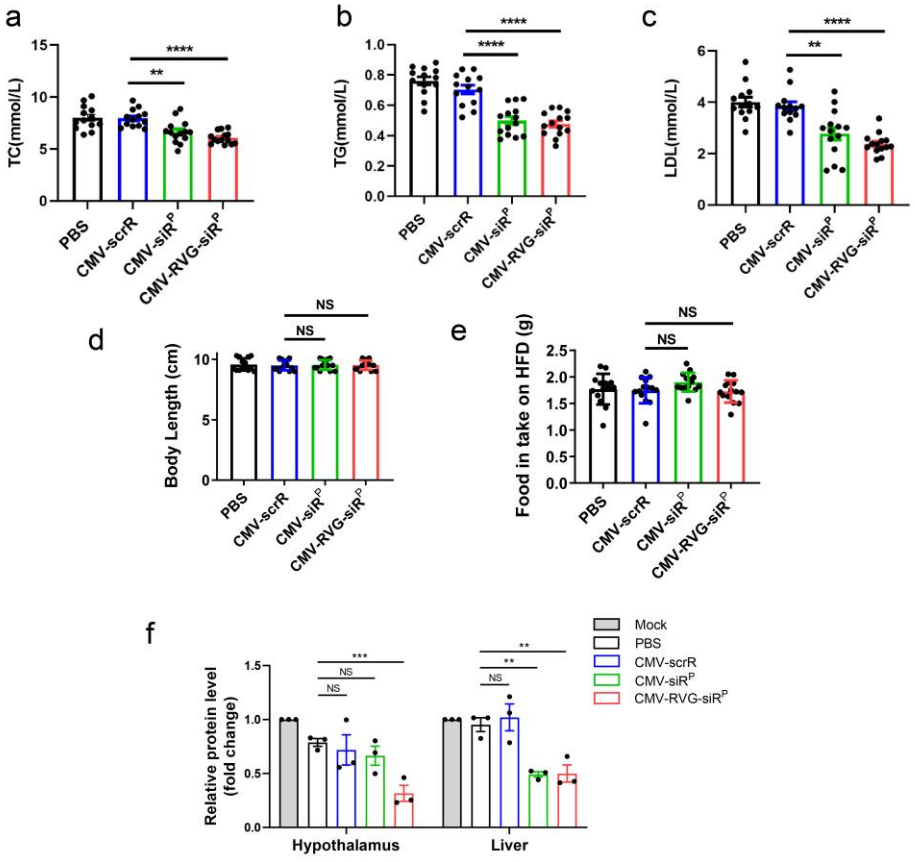

Supplement: Supplementary file 28 — Fig. S28 [file 41422_2021_491_MOESM28_ESM.pdf]
