# Supplementary material for: In vivo self-assembled small RNAs as a new generation of RNAi therapeutics
Source: Cell Res. 2021 Mar 29;31(6):631–48. doi: 10.1038/s41422-021-00491-z (PMC8169669; doi:10.1038/s41422-021-00491-z)

Fig. S29. Intravenous injection of the CMV-RVG-siR^P circuit increases insulin sensitivity and attenuates fatty liver in a mouse model of obesity. Male C57BL/6J mice at 3 weeks of age were placed on a HFD for 12 weeks. Mice rapidly gained weight and became obese. Mice were then maintained on a HFD and treated with PBS or 5 mg/kg CMV-scrR, CMV-siR^P or CMV-RVG-siR^P circuit through tail vein injection for a total of 12 times over 24 days. After treatment, insulin sensitivity and fatty liver degree were evaluated. **(a)** Basal insulin levels in serum (n = 14 in each group). **(b)** Quantitative RT-PCR analysis of glucose synthesis gene (G6Pase) and lipid synthesis genes (FAS, HMGSC1, SREBP-1A, SREBP-1C and SREBP2) in mouse livers. **(c)** Histopathological examination of mouse livers. Scale bar: 100 µm. Values are presented as the means ± SEM. Significance was determined using one-way ANOVA followed by Dunnett's multiple comparison. * $p < 0.05$; ** $p < 0.01$; *** $p < 0.005$; NS, not significant.

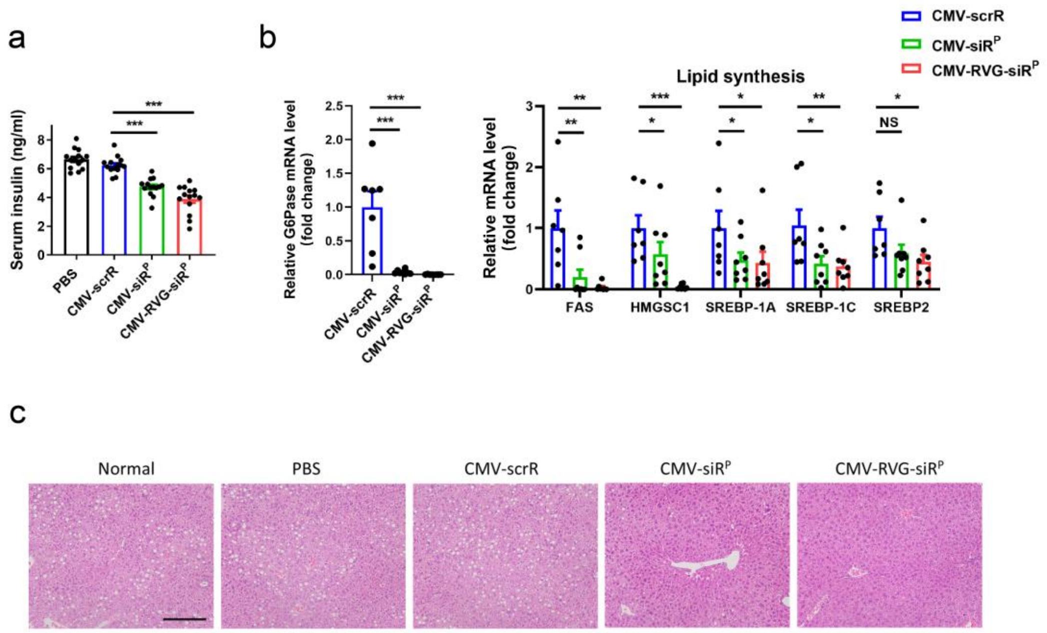

Supplement: Supplementary file 29 — Fig. S29 [file 41422_2021_491_MOESM29_ESM.pdf]
