# Supplementary material for: In vivo self-assembled small RNAs as a new generation of RNAi therapeutics
Source: Cell Res. 2021 Mar 29;31(6):631–48. doi: 10.1038/s41422-021-00491-z (PMC8169669; doi:10.1038/s41422-021-00491-z)

Fig. S30. Evaluation of the effect of CMV-RVG-siR^P circuit in *ob/ob* mice. The *ob/ob* mice were intravenously injected with PBS or 5 mg/kg CMV-scrR, CMV-siR^P or CMV-RVG-siR^P circuit for a total of 12 times over 24 days. Body weights were monitored during treatment. After treatment, fat mass and glucose homeostasis were evaluated. **(a)** Body weight curves (n = 14 in each group). **(b)** Weights of epididymal fat pads (n = 14 in each group). **(c)** Mouse GTT results (n = 8 in each group). **(d)** Mouse ITT results (n = 8 in each group). **(e)** Histopathological examination of mouse livers. Scale bar: 100 μm. Values are presented as the means ± SEM. Significance was determined using one-way ANOVA followed by Dunnett's multiple comparison. * $p < 0.05$; ** $p < 0.01$; NS, not significant.

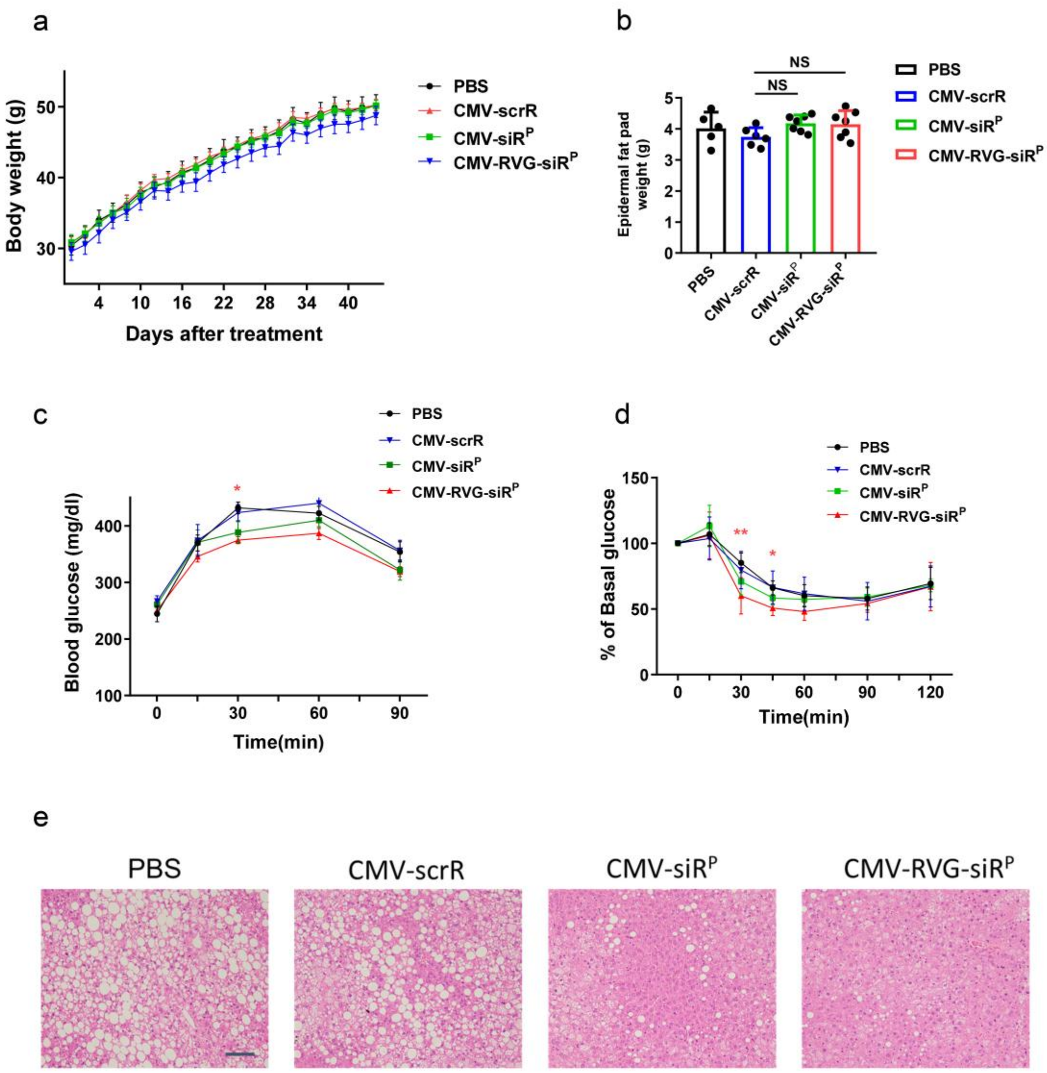

Supplement: Supplementary file 30 — Fig. S30 [file 41422_2021_491_MOESM30_ESM.pdf]
