# Supplementary material for: In vivo self-assembled small RNAs as a new generation of RNAi therapeutics
Source: Cell Res. 2021 Mar 29;31(6):631–48. doi: 10.1038/s41422-021-00491-z (PMC8169669; doi:10.1038/s41422-021-00491-z)

Fig. S31. Comparison of EGFR silencing efficiency in wild-type and nude mice after intravenous injection of the genetic circuit. Wild-type C57BL/6J mice and nude mice were intravenously injected with 5 mg/kg CMV-scrR, CMV-siR^{E} or CMV-RVG-siR^{E+T} circuit every 2 days for a total of 7 times. After treatment, mice were sacrificed, and the EGFR silencing efficiency was compared between wild-type and nude mice. **(a)** Western blot analysis of EGFR protein levels in the lung and brain of wild-type and nude mice. Shown are representative western blots. **(b-c)** Quantitation of EGFR protein levels in the lung and brain of wild-type and nude mice (n = 4 in each group). Values are presented as the means ± SEM. Significance was determined using one-way ANOVA followed by Dunnett's multiple comparison. * $p < 0.05$; ** $p < 0.01$; NS, not significant.

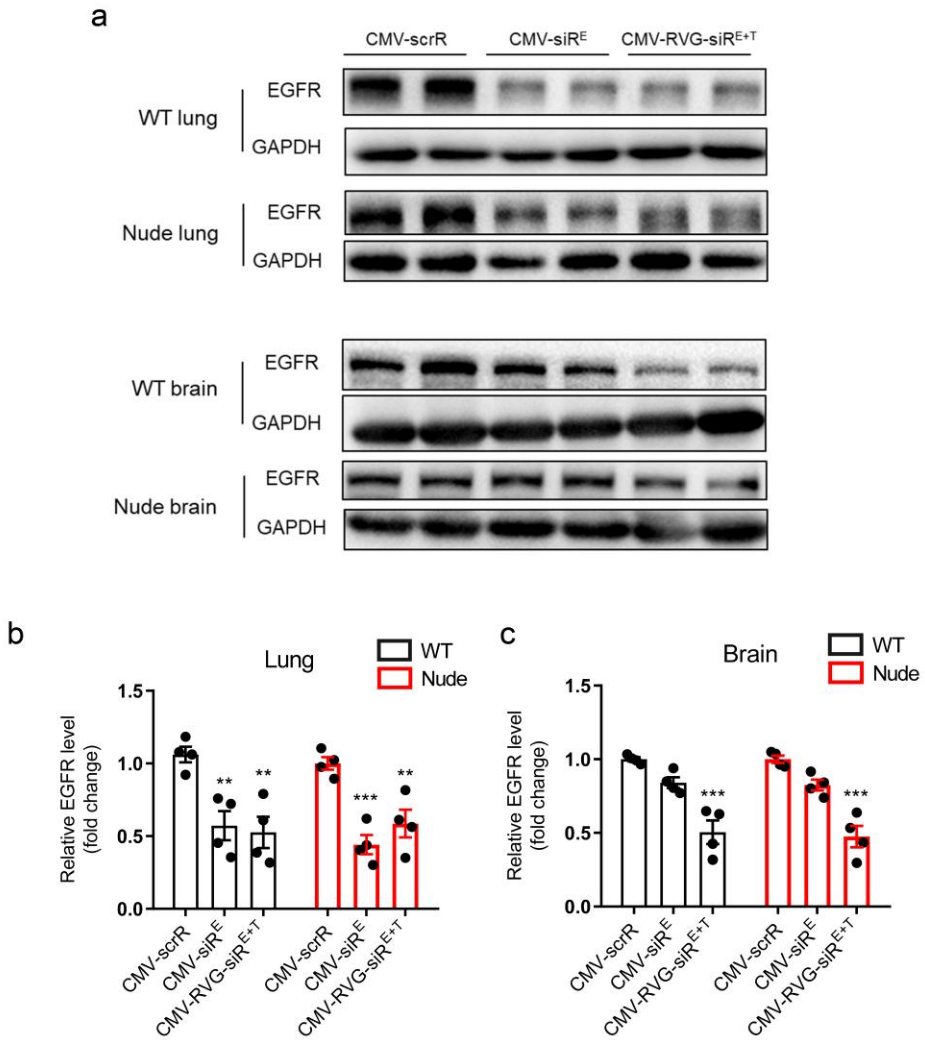

Supplement: Supplementary file 31 — Fig. S31 [file 41422_2021_491_MOESM31_ESM.pdf]
