# Supplementary material for: In vivo self-assembled small RNAs as a new generation of RNAi therapeutics
Source: Cell Res. 2021 Mar 29;31(6):631–48. doi: 10.1038/s41422-021-00491-z (PMC8169669; doi:10.1038/s41422-021-00491-z)

Fig. S32. Measurement of EGFR mRNA and protein levels in various tissues after intravenous injection of the genetic circuits. C57BL/6J mice were intravenously injected with PBS or 5 mg/kg CMV-scrR or CMV-siR^E circuit every 2 days for a total of 7 times. After treatment, mice were sacrificed and tissue samples were collected. **(a)** Quantitative RT-PCR analysis of EGFR mRNA levels in liver, lung, kidney, pancreas and spleen (n = 3 in each group). **(b)** Representative western blot analysis of EGFR protein levels in liver, lung, kidney, pancreas and spleen. Values are presented as the means ± SEM. Significance was determined using one-way ANOVA followed by Dunnett's multiple comparison. * $p < 0.05$; ** $p < 0.01$; NS, not significant.

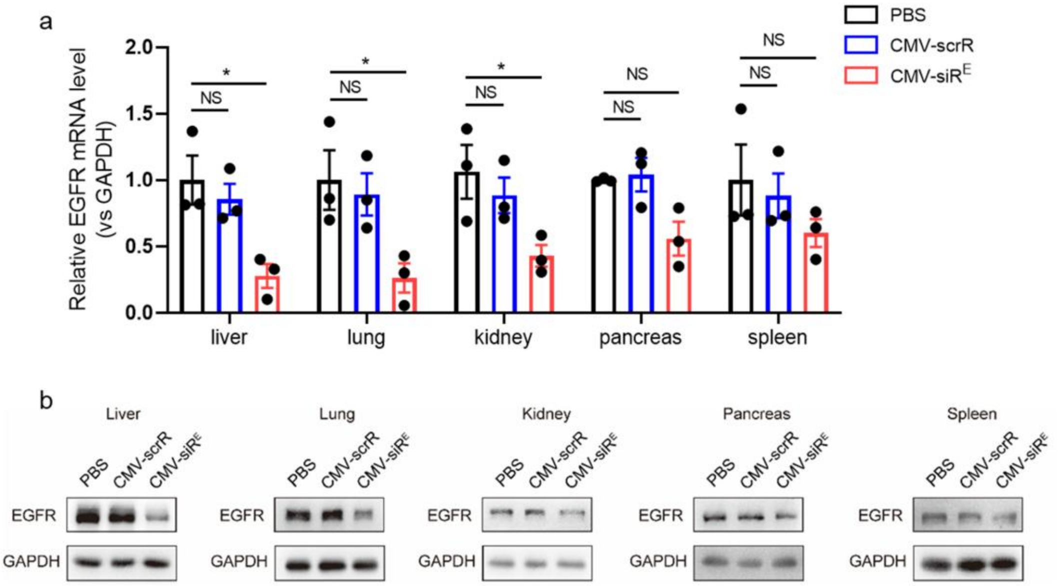

Supplement: Supplementary file 32 — Fig. S32 [file 41422_2021_491_MOESM32_ESM.pdf]
