# Supplementary figures and images for: In vivo self-assembled small RNAs as a new generation of RNAi therapeutics
Source: Cell Res. 2021 Mar 29;31(6):631–48. doi: 10.1038/s41422-021-00491-z (PMC8169669; doi:10.1038/s41422-021-00491-z)

**Fig. S33. Maps of the plasmids used to express genetic circuits.**

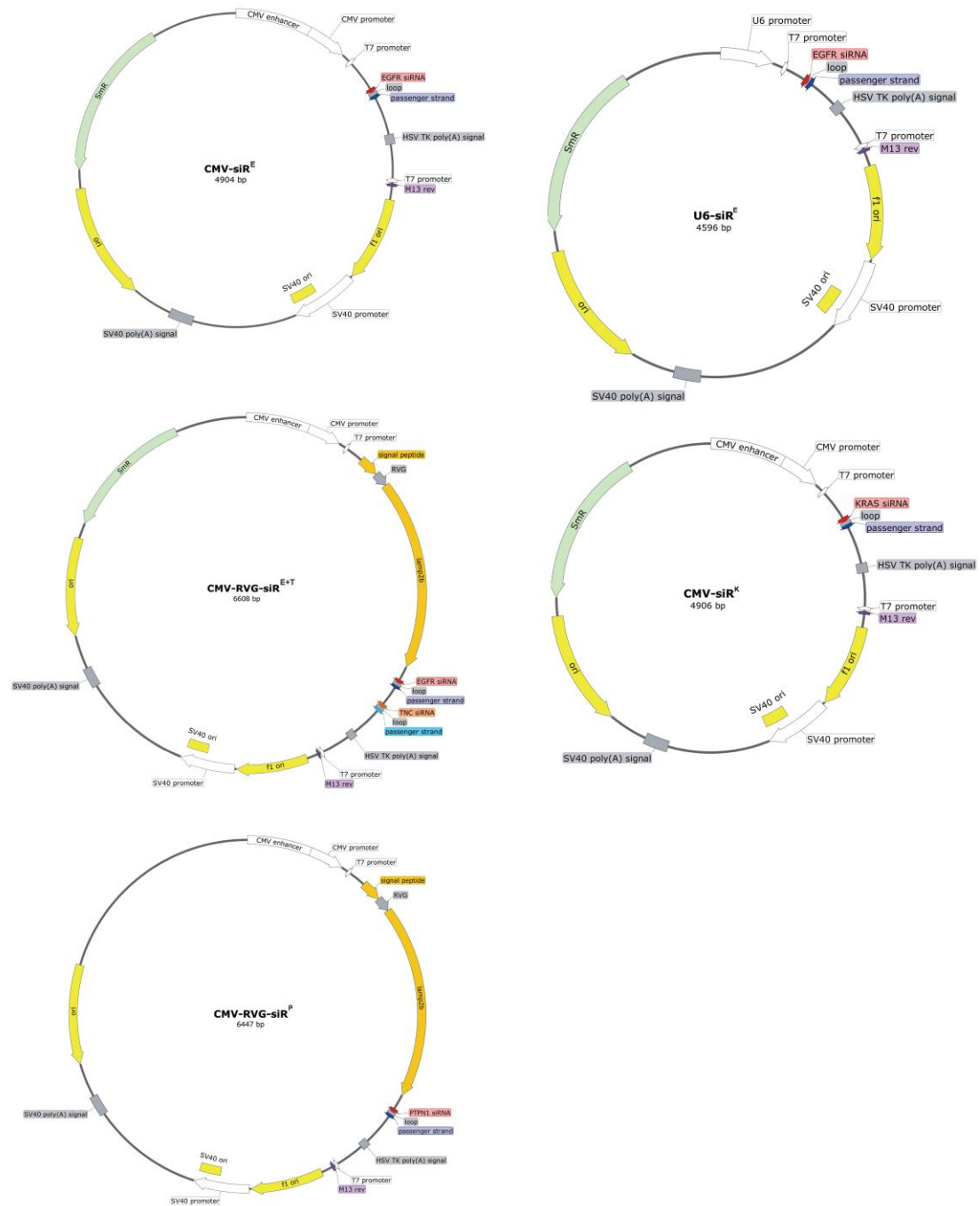

Supplement: Supplementary file 33 — Fig. S33 [file 41422_2021_491_MOESM33_ESM.pdf]
