# Supplementary material for: In vivo self-assembled small RNAs as a new generation of RNAi therapeutics
Source: Cell Res. 2021 Mar 29;31(6):631–48. doi: 10.1038/s41422-021-00491-z (PMC8169669; doi:10.1038/s41422-021-00491-z)

Fig. S3. Characterization of the physical properties of exosomes. HEK293T cells were transfected with equal amounts of CMV-scrR, CMV-siR^{E} or CMV-RVG-siR^{E+T} circuit. Twenty-four hours after treatment, exosomes were purified from culture medium of HEK293T cells and characterized by NTA, TEM and enrichment of exosomal markers. **(a)** Size distribution of purified exosomes determined by NTA. **(b)** Representative TEM images of purified exosomes. Scale bar: 75 nm. **(c)** Western blot analysis of specific exosomal markers (TSG101, CD63 and CD9) in purified exosomes and cell culture medium. An equal amount of total protein was loaded in each lane.

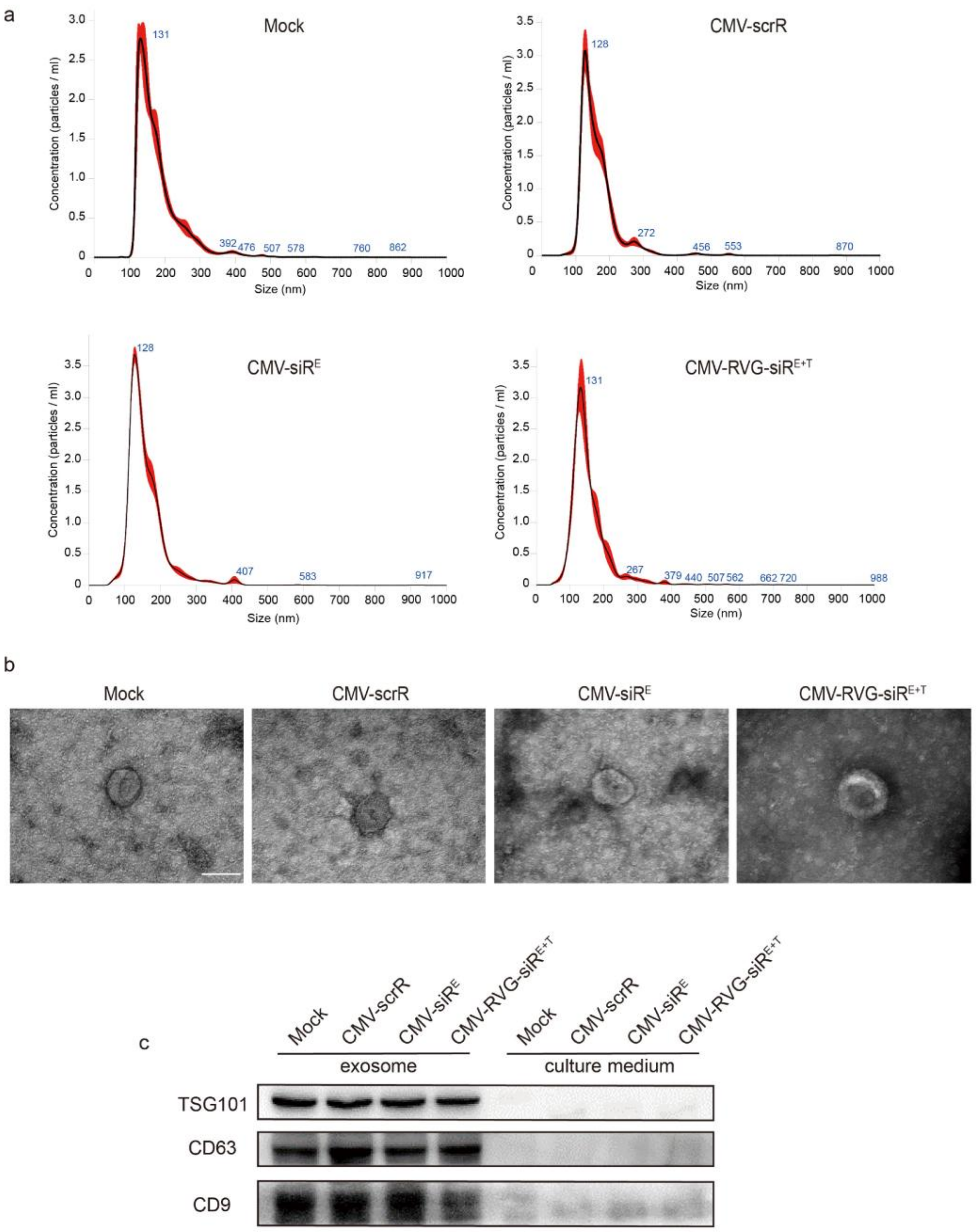

Supplement: Supplementary file 3 — Fig. S3 [file 41422_2021_491_MOESM3_ESM.pdf]
